# Supplementary material for: Sensor NLR immune proteins activate oligomerization of their NRC helpers in response to plant pathogens
Source: EMBO J. 2022 Dec 29;42(5):e111519. doi: 10.15252/embj.2022111519 (PMC9975940; doi:10.15252/embj.2022111519)
Supplement: Supplementary file 1 — Appendix S1 [file EMBJ-42-e111519-s011.pdf]

Sensor NLR immune proteins activate oligomerization of their NRC helpers

Mauricio P. Contreras[1], Hsuan Pai[1], Yasin Tumtas[2], Cian Duggan[2], Enoch Lok Him Yuen[2], Angel Vergara Cruces[1,‡], Jiorgos Kourelis[1], Hee-Kyung Ahn[1], Kim-Teng Lee[3], Chih-Hang Wu[3], Tolga O. Bozkurt[2], Lida Derevnina[1,†,*] and Sophien Kamoun[1*]

1: The Sainsbury Laboratory, University of East Anglia, Norwich, United Kingdom.
2: Department of Life Sciences, Imperial College, London, United Kingdom.
3: Institute of Plant and Microbial Biology, Academia Sinica, Taipei, Taiwan.

‡Current address: John Innes Centre, University of East Anglia, Norwich, United Kingdom.

†Current address: Crop Science Centre, Department of Plant Sciences, University of Cambridge, Cambridge, United Kingdom.

*Correspondence to: ld645@cam.ac.uk (LD); sophien.kamoun@tsl.ac.uk (SK)

Appendix

Table of contents

Appendix Figure S1: MADA motif mutants of NRC2 are unable to trigger cell death…………………………………………………………………………2

Appendix Figure S2: C-terminally tagged sensor and helper NLRs retain the capacity to trigger hypersensitive cell death……………………………………....3

Appendix Figure S3: C-terminally 6xHA tagged sensor NLRs retain the capacity to mediate cell death……………………………………………………………..4

Appendix Figure S4: Fluorescent protein-tagged Rx and NRC2 retain cell death-mediating capacity and can oligomerize upon activation………………………....5

Appendix Table S1: List of primers and constructs used in this study…………6

Appendix Figure S1:

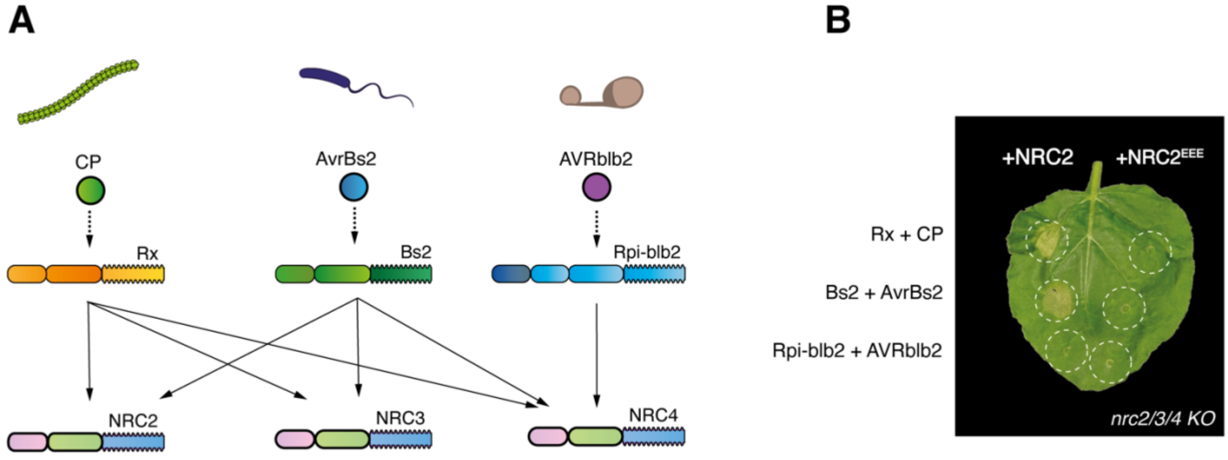

Appendix Figure S1: MADA motif mutants of NRC2 are unable to trigger cell death
(**A**) The Solanaceous NRC immune receptor network. Sensor NLRs such as Rx, Bs2 and Rpi-blb2 have specialized in detecting effectors from pathogens as diverse as viruses, bacteria and oomycetes. Sensors in the NRC network signal, sometimes redundantly, through their downstream helper NLRs, the NRCs. Different sensors exhibit different helper specificities. For example, Rx and Bs2 which recognize CP and AvrBs2 respectively, can signal through NRC2, NRC3, and NRC4. Rpi-blb2, which recognizes AVRblb2, can only signal through NRC4. Some sensor NLRs exhibit N-terminal extensions, represented in dark blue. (**B**) Unlike NRC2, NRC2^{EEE} does not complement Rx/CP and Bs2/AvrBs2-triggered hypersensitive cell death in leaves of *nrc2/3/4 N. benthamiana* CRISPR mutant lines. Representative leaves infiltrated with the appropriate constructs were photographed 5-7 days after infiltration. NRC2 and NRC2^{EEE} constructs are C-terminally 4xMyc-tagged. All effectors used are C-terminally GFP-tagged. All sensors used are C-terminally 6xHA tagged. One representative leaf is shown.

Appendix Figure S2:

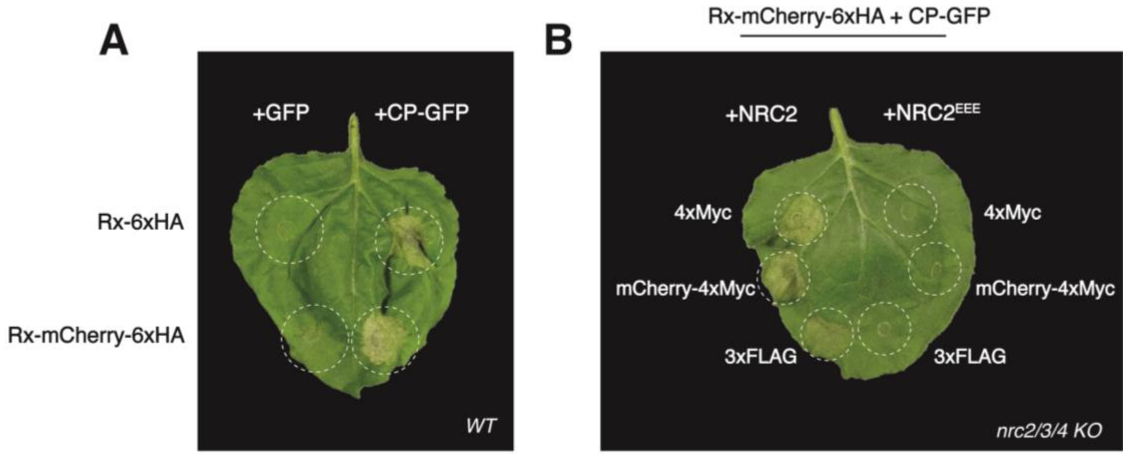

Appendix Figure S2: C-terminally tagged sensor and helper NLRs retain the capacity to trigger hypersensitive cell death.

(**A**) Much like C-terminally 6xHA tagged Rx, C-terminally mCherry-6xHA tagged Rx can mediate hypersensitive cell death when activated by CP. Representative leaves of *WT N. benthamiana* were infiltrated with the appropriate constructs and photographed 5-7 days after infiltration. C-terminal tags are indicated. Free GFP (+GFP) was used as a negative control for C-terminally GFP-tagged CP (+CP-GFP). One representative leaf is shown. (**B**) Rx-mCherry-6xHA is compatible with all C-terminally tagged versions of NRC2 tested. Rx/CP-triggered hypersensitive cell death was complemented by C-terminally 4xMyc, mCherry-4xMyc and 3xFLAG variants of NRC2 respectively in leaves of *nrc2/3/4 N. benthamiana* CRISPR mutant lines when Rx was C-terminally tagged with mCherry-6xHA. The corresponding $NRC2^{EEE}$ variants with the same C-terminal tag were no longer able to complement hypersensitive cell death. Representative leaves were infiltrated with the appropriate constructs and photographed 5-7 days after infiltration. One representative leaf is shown.

Appendix Figure S3:

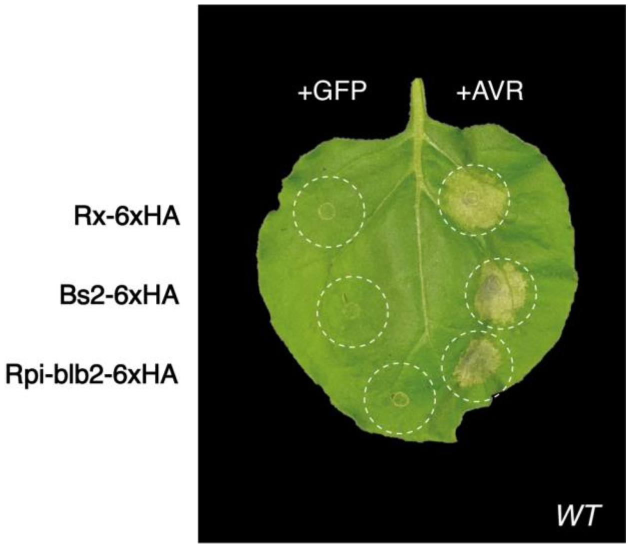

Appendix Figure S3: C-terminally 6xHA tagged sensor NLRs retain the capacity to mediate cell death.
C-terminally 6xHA tagged Rx, Bs2 and Rpi-blb2 can mediate hypersensitive cell death when activated by CP, AvrBs2 and AVRblb2, respectively. Representative leaves of *WT N. benthamiana* were infiltrated with the appropriate constructs and photographed 5-7 days after infiltration. Free GFP was used as a negative control for C-terminally GFP-tagged effectors (AVR). One representative leaf is shown.

Appendix Figure S4:

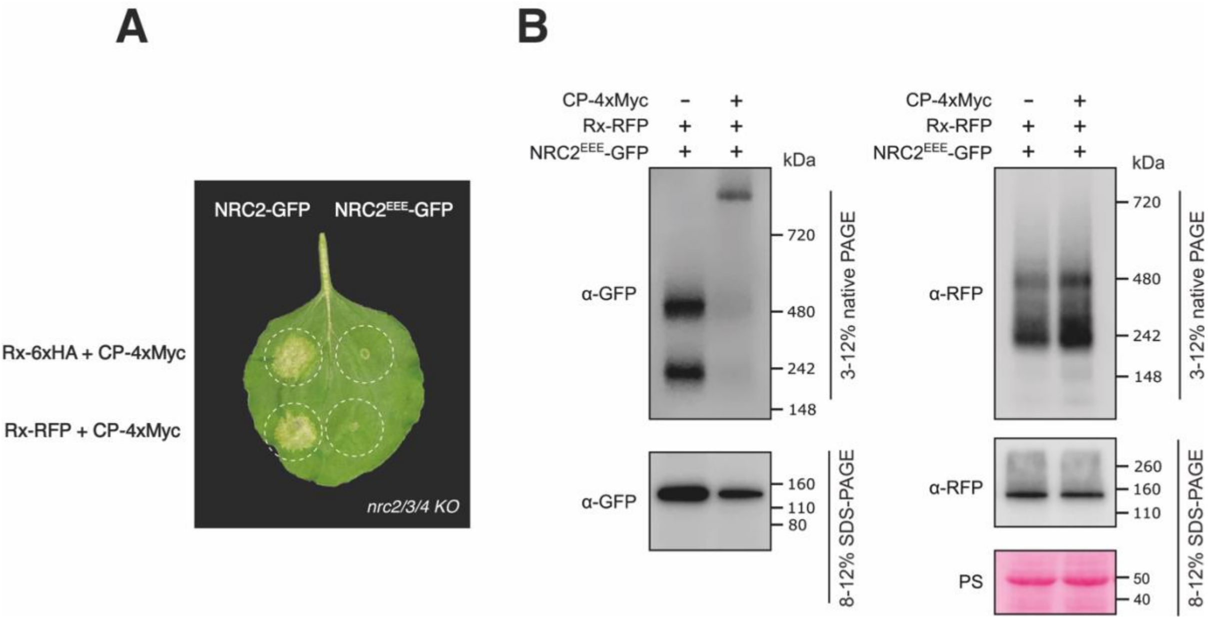

Appendix Figure S4: Fluorescent protein-tagged Rx and NRC2 retain cell death-mediating capacity and can oligomerize upon activation.

(**A**) C-terminally GFP-tagged NRC2 complements Rx/CP cell death in leaves of *nrc2/3/4 N. benthamiana* CRISPR mutant lines when Rx is C-terminally tagged with 6xHA or RFP. This cell death is not complemented with C-terminally GFP-tagged $NRC2^{EEE}$. Representative leaves were infiltrated with the appropriate constructs and photographed 5-7 days after infiltration. One representative leaf is shown. (**B**) BN-PAGE and SDS-PAGE assays performed in parallel on protein extracts used for membrane enrichment assays with inactive and activated C-terminally RFP-tagged Rx and C-terminally GFP-tagged $NRC2^{EEE}$. Total protein was extracted with a Tris-HCl-based buffer, as described in materials and methods. Extracts were run on native and denaturing PAGE assays in parallel and immunoblotted with the appropriate antisera labelled on the left. Approximate molecular weights (kDa) of the proteins are shown on the right. Rubisco loading control was carried out using Ponceau stain (PS). The experiment was repeated 2 times.

Appendix Table S1:

Appendix Table S1: List of primers and constructs used in this study.

Product	Antibiotic	Construct
pL2M-P19-2x35S::NbNRC2a-4xMyc	Kan	pJK-B2-090p-Myc
pL2M-P19-2x35S::Rx-6xHA	Kan	pJK-B2-178p-HA
pL2M-P19-2x35S::Bs2-6xHA	Kan	pJK-B2-219p-HA
pL2M-P19-2x35S::NbNRC2a^L9E/L13E/L17E-3xFLAG	Kan	pJK-B2-252p-FLAG
pL2M-P19-2x35S::NbNRC2a^L9E/L13E/L17E-4xMyc	Kan	pJK-B2-252p-Myc
pL2M-P19-2x35S::NbNRC2a-mEGFP	Kan	pMC-B2-203p-mEGFP
pL2M-P19-2x35S::NbNRC4^L9A/V10A/L14A-4xMyc	Kan	pMC-B2-179-4xMyc
pL2M-P19-2x35S::NbNRC2a^L9E/L13E/L17E-mEGFP	Kan	pMC-B2-204p-mEGFP
pL2M-P19-2x35S::Rpiblb2-6xHA	Kan	pMC-B2-164p-HA
pL2M-P19-2x35S::EGFP	Kan	pJK-B2-022p
pL2M-P19-2x35S::NbNRC2a-mCherry-4xMYC	Kan	pJK-B2-618p
pL2M-P19-2x35S::NbNRC2a^L9E/L13E/L17E-mCherry-4xMYC	Kan	pJK-B2-619p
pL2M-P19-2x35S::Rx-mCherry-6xHA	Kan	pJK-B2-620p
pL2M-P19-2x35S::AvrBs2-mEGFP	Kan	pJK-B2-800p
pL0M-CDS1ns-AvrBs2	Spec	pJK-0-364
pL2M-P19-2x35S::PVX^CP4_CP_30-142-4xMyc	Kan	pJK-B2-259p
pL2M-P19-2x35S::PVX^CP4_CP_30-142-EGFP	Kan	pMC-B2-142p-mEGFP
pL0M-CDS1ns-NbNRC2a	Spec	pJK-0-116
pL0M-CDS1ns-NbNRC2a^L9E/L13E/L17E	Spec	pJK-0-117
pL0M-CT-EGFP	Spec	pICSL50044
pL0M-CDS1ns-mCherry	Spec	pJK-0-071
pL0M-CDS1-RpiBlb2	Spec	pICSL80031
pCR8-CDS1ns-Rx	Spec	pCR8-Rx
pL0M-CT-3xFLAG	Spec	pICSL50007
pL0M-CT-6xHA	Spec	pICSL50009A
pL0M-CT-4xMyc	Spec	pICSL50010
pL1V2-P19/MCS	Kan/mRFP1	pJK268c_mRFP1
pL0M-P5U-2x35S-TMV	Spec	pICH51288
pL0M-3UT-t35S	Spec	pICH41414
pICH41155-DraIII	Spec	pICH41155

Product	Purpose	RE	Plasmids
pL2M-P19-2x35S::NbNRC2a-4xMyc	Binary Agro L2M	BsaI	pJK268c_mRFP1, pICH51288, pICH41414, pICSL50010, pJK-0-116
pL2M-P19-2x35S::Rx-6xHA	Binary Agro L2M	BsaI	pJK268c_mRFP1, pICH51288, pICH41414, pICSL50009A, pCR8-Rx
pL2M-P19-2x35S::Bs2-6xHA	Binary Agro L2M	BsaI	pJK268c_mRFP1, pICH51288, pICH41414, pICSL50009A
pL2M-P19-2x35S::NbNRC2a^L9E/L13E/L17E-3xFLAG	Binary Agro L2M	BsaI	pJK268c_mRFP1, pICH51288, pICH41414, pICSL50007, pJK-0-117
pL2M-P19-2x35S::NbNRC2a^L9E/L13E/L17E-4xMyc	Binary Agro L2M	BsaI	pJK268c_mRFP1, pICH51288, pICH41414, pICSL50010, pJK-0-117
pL2M-P19-2x35S::NbNRC2a-mEGFP	Binary Agro L2M	BsaI	pJK268c_mRFP1, pICH51288, pICH41414, pICSL50034, pJK-0-116
pL2M-P19-2x35S::NbNRC4^L9A/V10A/L14A-4xMyc	Binary Agro L2M	BsaI	pICH86988, pICSL50010, pCR8-NRC4AAA
pL2M-P19-2x35S::NbNRC2a^L9E/L13E/L17E-mEGFP	Binary Agro L2M	BsaI	pJK268c_mRFP1, pICH51288, pICH41414, pICSL50010, pJK-0-117
pL2M-P19-2x35S::Rpiblb2-6xHA	Binary Agro L2M	BsaI	pJK268c_mRFP1, pICH51288, pICH41414, pICSL50009A, pICSL80031
pL2M-P19-2x35S::EGFP	Binary Agro L2M	BsaI	pJK268c_mRFP1, pICH51288, pICH41414, pJK635
pL2M-P19-2x35S::NbNRC2a-mCherry-4xMYC	Binary Agro L2M	BsaI	pJK268c_mRFP1, pICH51288, pICH41414, pJK-0-071, pJK-0-116
pL2M-P19-2x35S::NbNRC2a^L9E/L13E/L17E-mCherry-4xMYC	Binary Agro L2M	BsaI	pJK-0-071, pICSL50010
pL2M-P19-2x35S::Rx-mCherry-6xHA	Binary Agro L2M	BsaI	pJK-0-071, pICSL50009A
pL2M-P19-2x35S::AvrBs2-mEGFP	Binary Agro L2M	BsaI	pJK268c_mRFP1, pICH51288, pICH41414, pICSL50044, pJK-0-364
pL0M-CDS1ns-AvrBs2	General L0M	DraIII	pICH41155
pL2M-P19-2x35S::PVX^CP4_CP_30-142-4xMyc	Binary Agro L2M	BsaI	pJK268c_mRFP1, pICH51288, pICH41414, pICSL50010
pL2M-P19-2x35S::PVX^CP4_CP_30-142-EGFP	Binary Agro L2M	BsaI	pJK268c_mRFP1, pICH51288, pICH41414, pICSL50044
pL0M-CDS1ns-NbNRC2a	General L0M	-	-
pL0M-CDS1ns-NbNRC2a^L9E/L13E/L17E	General L0M	BsaI	pICSL01005
pL0M-CT-EGFP	General L0M	-	-
pL0M-CDS1ns-mCherry	General L0M	-	-
pL0M-CDS1-RpiBlb2	General L0M	-	-
pCR8-CDS1ns-Rx	General L0M	-	-
pL0M-CT-3xFLAG	General L0M	-	-
pL0M-CT-6xHA	General L0M	-	-
pL0M-CT-4xMyc	General L0M	-	-
pL1V2-P19/MCS	Cloning	-	pICSL4723, pJK263, pICH47742_mRFP1, pICH41744
pL0M-P5U-2x35S-TMV	General L0M	-	-
pL0M-3UT-t35S	General L0M	-	-
pICH41155-DraIII	Cloning	-	-

Product	Template	Primers/Synthesis
pL2M-P19-2x35S::NbNRC2a-4xMyc	-	-
pL2M-P19-2x35S::Rx-6xHA	-	-
pL2M-P19-2x35S::Bs2-6xHA	pMD1-Bs2-HA	
pL2M-P19-2x35S::NbNRC2a^L9E/L13E/L17E-3xFLAG	-	-
pL2M-P19-2x35S::NbNRC2a^L9E/L13E/L17E-4xMyc		
pL2M-P19-2x35S::NbNRC2a-mEGFP		
pL2M-P19-2x35S::NbNRC4^L9A/V10A/L14A-4xMyc		
pL2M-P19-2x35S::NbNRC2a^L9E/L13E/L17E-mEGFP	-	-
pL2M-P19-2x35S::Rpiblb2-6xHA	-	-
pL2M-P19-2x35S::EGFP	-	-
pL2M-P19-2x35S::NbNRC2a-mCherry-4xMYC		
pL2M-P19-2x35S::NbNRC2a^L9E/L13E/L17E-mCherry-4xMYC	pJK-B2-252p	TTGGTCTCAGCTTCTCTAGCTAGAGTCGATC/TTGGTCTCACATTCCACCGAGATCGGGAGGGAATATAGAG
pL2M-P19-2x35S::Rx-mCherry-6xHA	pJK-B2-178p-HA	TTGGTCTCAGCTTCTCTAGCTAGAGTCGATC/TTGGTCTCACCATTCCACCCTCGACATTATTGCG
pL2M-P19-2x35S::AvrBs2-mEGFP	-	-
pL0M-CDS1ns-AvrBs2	-	
pL2M-P19-2x35S::PVX^CP4_CP_30-142-4xMyc	-	
pL2M-P19-2x35S::PVX^CP4_CP_30-142-EGFP	-	
pL0M-CDS1ns-NbNRC2a	-	-
pL0M-CDS1ns-NbNRC2a^L9E/L13E/L17E	pJK-0-116	
pL0M-CT-EGFP	-	-
pL0M-CDS1ns-mCherry	pJK636	TTGAAGACAATTCGTGAGACCACGAAGTGGCTC/TTGAAGACAACGAACCCTTGTACAGCTCGTCCATGC
pL0M-CDS1-RpiBlb2	-	-
pCR8-CDS1ns-Rx	-	-
pL0M-CT-3xFLAG	-	-
pL0M-CT-6xHA	-	-
pL0M-CT-4xMyc	-	-
pL1V2-P19/MCS	-	-
pL0M-P5U-2x35S-TMV	-	-
pL0M-3UT-t35S	-	-
pICH41155-DraIII	-	-

Product	Comment	Publication	OD
pL2M-P19-2x35S::NbNRC2a-4xMyc	NRC2-Myc	This work	0.3
pL2M-P19-2x35S::Rx-6xHA	Rx-HA	This work	0.3
pL2M-P19-2x35S::Bs2-6xHA	Bs2-HA	This work	0.3
pL2M-P19-2x35S::NbNRC2a^L9E/L13E/L17E-3xFLAG	NRC2EEE-FLAG	This work	0.3
pL2M-P19-2x35S::NbNRC2a^L9E/L13E/L17E-4xMyc	NRC2EEE-Myc	This work	0.3
pL2M-P19-2x35S::NbNRC2a-mEGFP	NRC2-mEGFP	This work	0.3
pL2M-P19-2x35S::NbNRC4^L9A/V10A/L14A-4xMyc	NRC4AAA-4xMyc	This work	0.3
pL2M-P19-2x35S::NbNRC2a^L9E/L13E/L17E-mEGFP	NRC2EEE-GFP	This work	0.3
pL2M-P19-2x35S::Rpiblb2-6xHA	Rpiblb2-HA	This work	0.3
pL2M-P19-2x35S::EGFP	EGFP	Kourelis, Marchal et al., 2021	0.2
pL2M-P19-2x35S::NbNRC2a-mCherry-4xMYC	NRC2-mCherry-Myc	This work	0.3
pL2M-P19-2x35S::NbNRC2a^L9E/L13E/L17E-mCherry-4xMYC	NRC2EEE-mCherry-Myc	This work	0.3
pL2M-P19-2x35S::Rx-mCherry-6xHA	Rx-mCherry-HA	This work	0.3
pL2M-P19-2x35S::AvrBs2-mEGFP	AvrBs2-EGFP; GeneArt codon-optimized for *Nicotiana benthamiana*	This work	0.2
pL0M-CDS1ns-AvrBs2	GeneArt codon-optimized for *Nicotiana benthamiana*	This work	-
pL2M-P19-2x35S::PVX^CP4_CP_30-142-4xMyc	CP-Myc; GeneArt codon-optimized for *Nicotiana benthamiana*	This work	0.2
pL2M-P19-2x35S::PVX^CP4_CP_30-142-EGFP	CP-EGFP; GeneArt codon-optimized for *Nicotiana benthamiana*	This work	0.2
pL0M-CDS1ns-NbNRC2a	-	Kourelis, Contreras et al., 2021	-
pL0M-CDS1ns-NbNRC2a^L9E/L13E/L17E	-	This work	-
pL0M-CT-EGFP	-	TSL SynBio	-
pL0M-CDS1ns-mCherry	-	This work	-
pL0M-CDS1-RpiBlb2	-	TSL SynBio	-
pCR8-CDS1ns-Rx	-	Adachi et al., 2019	-
pL0M-CT-3xFLAG	-	Engler et a., 2014; Addgene #50308	-
pL0M-CT-6xHA	-	TSL SynBio	-
pL0M-CT-4xMyc	-	Engler et a., 2014; Addgene #50310	-
pL1V2-P19/MCS	pICSL4723 acceptor backbone with p19 module fixed in R1 position, clone into F2 using Bsa	Kourelis et al., 2020	-
pL0M-P5U-2x35S-TMV	-	Engler et a., 2014; Addgene #50269	-
pL0M-3UT-t35S	-	Engler et a., 2014; Addgene #50337	-
pICH41155-DraIII	General L0M acceptor, DraIII	TSL SynBio	-
