# Supplementary material for: Sensor NLR immune proteins activate oligomerization of their NRC helpers in response to plant pathogens
Source: EMBO J. 2022 Dec 29;42(5):e111519. doi: 10.15252/embj.2022111519 (PMC9975940; doi:10.15252/embj.2022111519)
Supplement: Supplementary file 2 — Expanded View Figures PDF [file EMBJ-42-e111519-s004.pdf]

Expanded View Figures

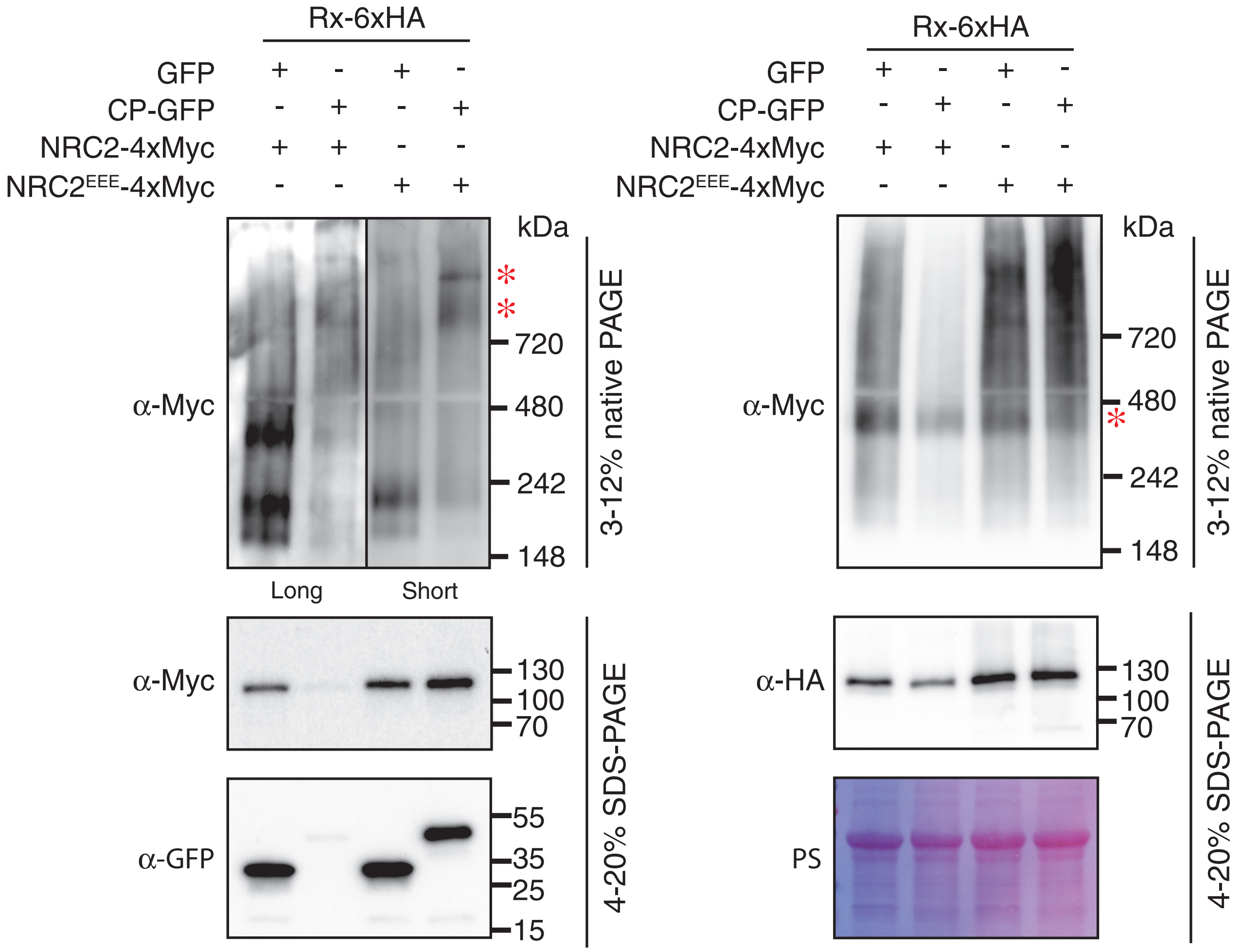

Figure EV1. NRC2 with an intact N-terminal MADA motif also oligomerizes upon Rx-mediated activation.

BN-PAGE and SDS–PAGE assays with inactive and activated Rx-NRC2. C-terminally 6xHA tagged Rx and C-terminally 4xMyc-tagged NRC2 or NRC2^{EEE} were co-expressed with either free GFP or C-terminally GFP-tagged CP. Total protein was extracted with a Tris–HCl-based buffer, as described in materials and methods. Extracts were run on native and denaturing PAGE assays in parallel and immunoblotted with the appropriate antisera labeled on the left. Approximate molecular weights (kDa) of the proteins are shown on the right. Red asterisks indicate bands corresponding to the activated NRC2 complex (α-Myc blot) and Rx (α-HA blot). Given that the ongoing cell death triggered by NRC2 activation resulted in lower protein accumulation, we showed different exposures (long and short) as indicated by the black line. Rubisco loading control was carried out using Ponceau stain (PS). The experiment was repeated 2 times.

Source data are available online for this figure.

© 2022 The Authors

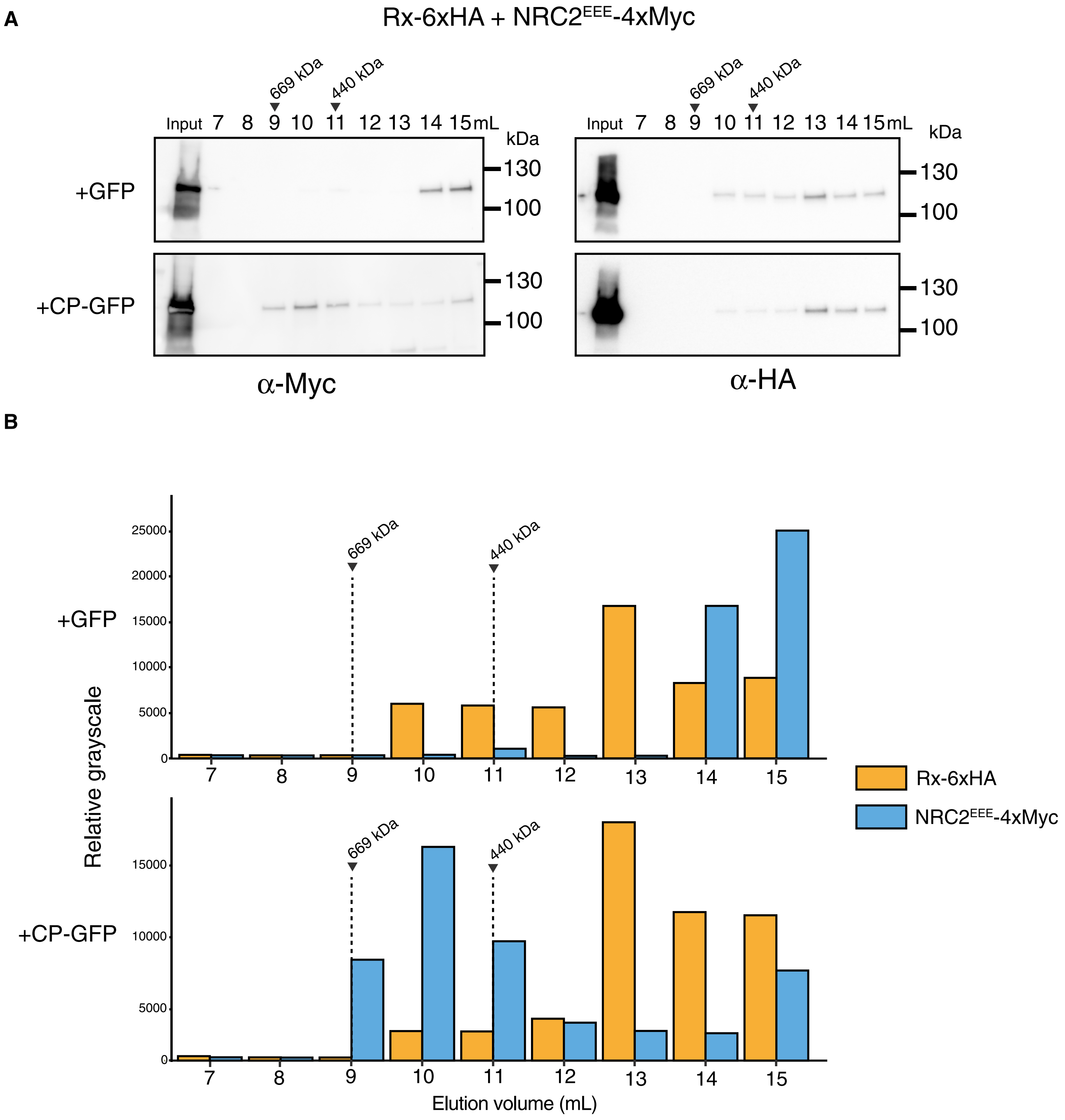

Figure EV2. Rx-mediated NRC2 oligomerization can be visualized using gel filtration assays.

A Gel filtration assays with inactive and activated Rx-NRC2^{EEE}. C-terminally 6xHA tagged Rx and C-terminally 4xMyc-tagged NRC2^{EEE} were co-expressed with either free GFP or C-terminally GFP-tagged CP. Total protein extracts were run on an S200 10/300 analytical column. A range of fractions were ran on SDS–PAGE and immunoblotted with the appropriate antisera labeled below. Approximate molecular weights (kDa) of the proteins are shown on the right.

B Relative gray scales indicate the arbitrary densitometry units of different proteins as visualized by immunoblotting. Elution volume of standard molecular masses (669 kDa and 440 kDa) is shown above. The experiment was repeated 2 times and a representative image is shown.

Source data are available online for this figure.

 © 2022 The Authors
