# Supplementary material for: Sensor NLR immune proteins activate oligomerization of their NRC helpers in response to plant pathogens
Source: EMBO J. 2022 Dec 29;42(5):e111519. doi: 10.15252/embj.2022111519 (PMC9975940; doi:10.15252/embj.2022111519)
Supplement: Supplementary file 3 — Movie EV1 [file EMBJ-42-e111519-s003.zip › MovieEV1_FigureLegend.docx]

**Movie EV1:**

**Movie EV1: NRC2 forms PM-associated puncta upon activation by *Potato virus X* coat protein and Rx.**

3-D movies of inactive (left) or CP-activated (right) NRC2^EEE^-GFP (shown in yellow) co-expressed with Rx-RFP (not shown) in leaves of *nrc2/3/4 N. benthamiana* CRISPR mutant lines. CP was C-terminally 4xMyc-tagged. Free 4xMyc tag was used for the inactive negative control.
