# Supplementary material for: Sensor NLR immune proteins activate oligomerization of their NRC helpers in response to plant pathogens
Source: EMBO J. 2022 Dec 29;42(5):e111519. doi: 10.15252/embj.2022111519 (PMC9975940; doi:10.15252/embj.2022111519)
Supplement: Supplementary file 4 — Source Data for Expanded View and Appendix [file EMBJ-42-e111519-s009.zip › SD-FigEV2.pdf]

Figure EV2 Source Data

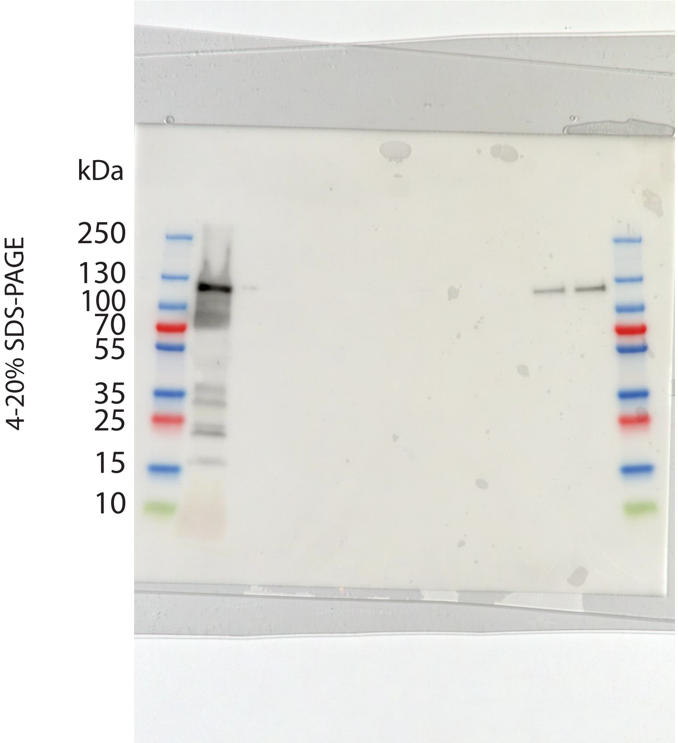

Inactive Myc detection + brightfield merge uncropped

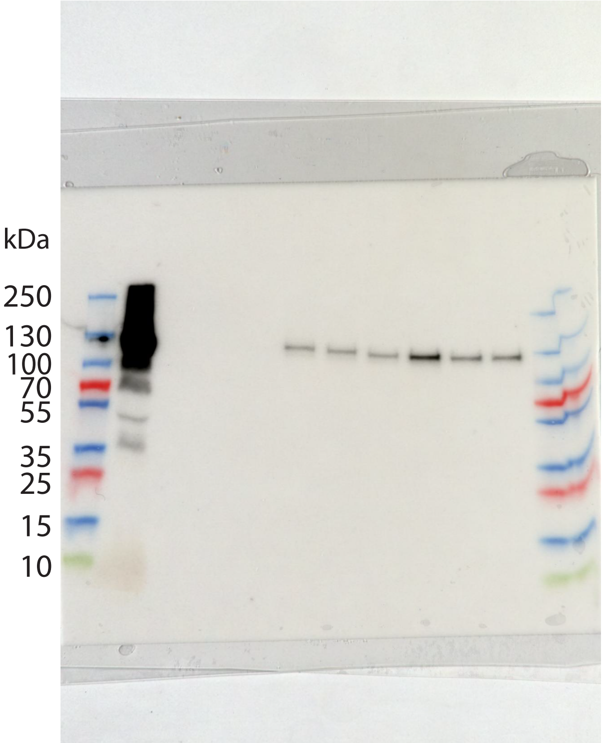

Inactive HA detection + brightfield merge uncropped

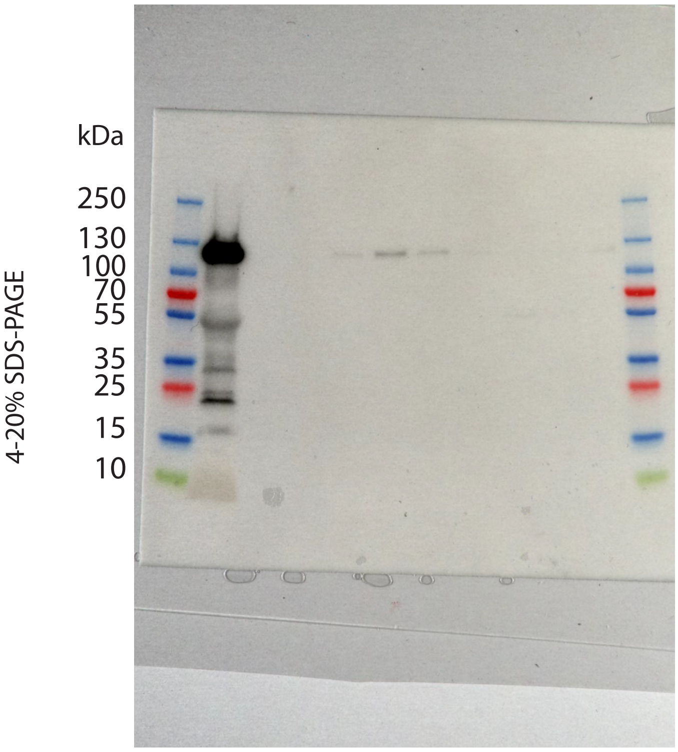

Activated Myc detection + brightfield merge uncropped

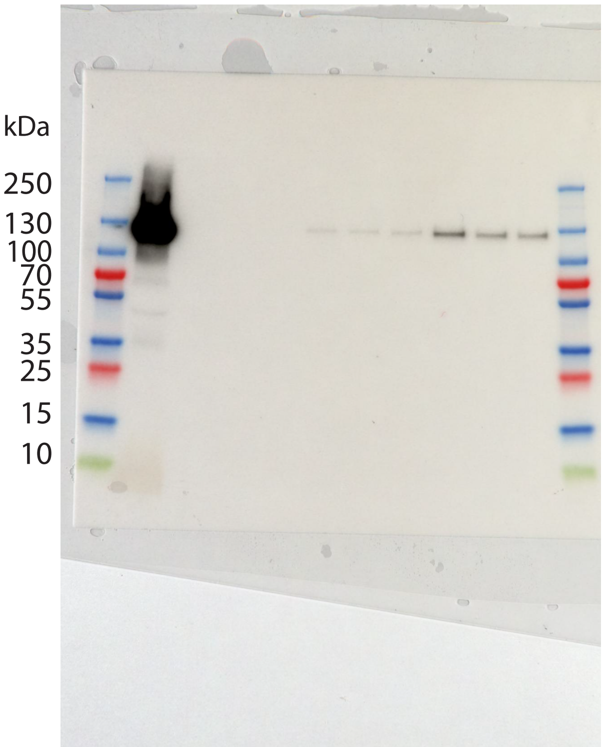

Activated HA detection + brightfield merge uncropped
