# Supplementary material for: Sensor NLR immune proteins activate oligomerization of their NRC helpers in response to plant pathogens
Source: EMBO J. 2022 Dec 29;42(5):e111519. doi: 10.15252/embj.2022111519 (PMC9975940; doi:10.15252/embj.2022111519)
Supplement: Supplementary file 5 — PDF+ [file EMBJ-42-e111519-s008.pdf]

Article

SOURCE DATA | TRANSPARENT PROCESS | OPEN ACCESS

THE EMBO JOURNAL

Sensor NLR immune proteins activate oligomerization of their NRC helpers in response to plant pathogens

Mauricio P Contreras[1], Hsuan Pai[1], Yasin Tumtas[2], Cian Duggan[2], Enoch Lok Him Yuen[2], Angel Vergara Cruces[1,†], Jiorgos Kourelis[1], Hee-Kyung Ahn[1], Kim-Teng Lee[3], Chih-Hang Wu[3], Tolga O Bozkurt[2], Lida Derevnina[1,*,‡] & Sophien Kamoun[1,**]

Abstract

Nucleotide-binding domain leucine-rich repeat (NLR) immune receptors are important components of plant and metazoan innate immunity that can function as individual units or as pairs or networks. Upon activation, NLRs form multiprotein complexes termed resistosomes or inflammasomes. Although metazoan paired NLRs, such as NAIP/NLRC4, form hetero-complexes upon activation, the molecular mechanisms underpinning activation of plant paired NLRs, especially whether they associate in resistosome hetero-complexes, is unknown. In asterid plant species, the NLR required for cell death (NRC) immune receptor network is composed of multiple resistance protein sensors and downstream helpers that confer immunity against diverse plant pathogens. Here, we show that pathogen effector-activation of the NLR proteins Rx (confers virus resistance), and Bs2 (confers bacterial resistance) leads to oligomerization of their helper NLR, NRC2. Activated Rx does not oligomerize or enter into a stable complex with the NRC2 oligomer and remains cytoplasmic. In contrast, activated NRC2 oligomers accumulate in membrane-associated puncta. We propose an activation-and-release model for NLRs in the NRC immune receptor network. This points to a distinct activation model compared with mammalian paired NLRs.

Keywords immune receptor; inflammasome; NB-LRR; NOD-like receptor; resistosome
Subject Category Plant Biology
DOI 10.15252/embj.2022111519 | Received 26 April 2022 | Revised 25 November 2022 | Accepted 2 December 2022 | Published online 29 December 2022
The EMBO Journal (2023) 42: e111519

Introduction

A class of intracellular immune receptors, known as NLRs (nucleotide-binding domain and leucine-rich repeat receptors), are key components of the innate immune systems of plants and metazoan. NLRs play an important role in mediating pathogen recognition and subsequent immune responses (Jones *et al*, 2016; Duxbury *et al*, 2021). In plants, NLRs can activate host defense by recognizing pathogen secreted virulence proteins, termed effectors. This recognition leads to immune signaling, often culminating in a form of programmed cell death known as the hypersensitive response (Jones & Dangl, 2006; Kourelis & Van Der Hoorn, 2018; Ngou *et al*, 2022). Similarly, metazoan NLRs are capable of sensing pathogen effectors and other classes of pathogen derived molecules, ultimately leading to a form of programmed cell death known as pyroptosis (Maekawa *et al*, 2011; Duxbury *et al*, 2016). Some plant and metazoan NLRs can function as single units, with one NLR protein mediating both effector/elicitor perception and subsequent downstream signaling. These are referred to as functional singleton NLRs (Adachi *et al*, 2019a, 2019b). However, NLRs can also function as genetically linked receptor pairs or in higher order configurations that can include genetically unlinked receptor networks (Wu *et al*, 2017, 2018). In these cases, the sensing and signaling functions are uncoupled in two distinct proteins. One NLR acts as the pathogen sensor, requiring a second NLR which acts as a helper (or executor) to mediate immune activation and disease resistance (Wu *et al*, 2018; Adachi *et al*, 2019b; Feehan *et al*, 2020). Although much progress has been made in recent years regarding the biochemical mechanisms of how singleton NLRs activate and signal (Wang *et al*, 2019a; Ma *et al*, 2020; Martin *et al*, 2020; preprint: Förderer *et al*, 2022), our understanding of how paired and networked NLRs operate remains limited.

1 The Sainsbury Laboratory, University of East Anglia, Norwich, UK
2 Department of Life Sciences, Imperial College, London, UK
3 Institute of Plant and Microbial Biology, Academia Sinica, Taipei, Taiwan
*Corresponding author. Tel: +44 0 1223 342200; E-mail: ld645@cam.ac.uk
**Corresponding author. Tel: +44 0 1603 450410; E-mail: sophien.kamoun@tsl.ac.uk
†Present Address: John Innes Centre, University of East Anglia, Norwich, UK
‡Present Address: Department of Plant Sciences, Crop Science Centre, University of Cambridge, Cambridge, UK

© 2022 The Authors. Published under the terms of the CC BY 4.0 license.

Plant and metazoan NLRs belong to the signal transduction ATPases with numerous domains (STAND) superfamily. They usually exhibit a modular, tri-partite structure with an N-terminal signaling domain, a central nucleotide binding domain and a C-terminal domain with superstructure forming repeats (Kim *et al*, 2016; Duxbury *et al*, 2021; Kourelis *et al*, 2021b). The N-terminal domains of NLRs can broadly be used to classify these receptors into distinct groups which, in plants, tend to also cluster together in phylogenetic analyses. Plant NLR N-terminal domains can be either coiled-coil-type (CC) NLRs, G10-type CC (CC_{G10}) NLRs, RPW8-type CC (CC_R) NLRs or toll/interleukin-1 receptor-type (TIR) NLRs, whereas metazoan NLRs usually exhibit either N-terminal PYRIN or caspase recruitment domains (CARD) (Lechtenberg *et al*, 2014; Kim *et al*, 2016; Lee *et al*, 2021; Kourelis *et al*, 2021b). The central nucleotide binding domain is the defining feature of NLRs and is typically a nucleotide-binding adaptor shared by APAF-1, plant R proteins and CED-4 (NB-ARC) domain in plants, while metazoan NLRs can have either an NB-ARC or a NAIP, C2TA, HET-E and TP1 (NACHT) domain. As for their superstructure forming repeats, these can be either leucine-rich repeats (LRR) or tetratricopeptide repeats (TPR) (Duxbury *et al*, 2021; Kourelis *et al*, 2021b).

While the general principles of how NLRs sense their ligands are well-understood (Kourelis & Van Der Hoorn, 2018; Ngou *et al*, 2022), our knowledge of the molecular mechanisms that underpin NLR activation and signaling are more limited. In the case of mammalian NLRs, activation leads to oligomerization and formation of higher order wheel-like complexes, termed inflammasomes. Inflammasomes ultimately recruit caspases which act as the final executors of programmed cell-death (Lechtenberg *et al*, 2014; Kim *et al*, 2016). In contrast, the mechanisms of plant NLR activation were not well understood until the recent elucidation of the structure of inactive and activated ZAR1, a conserved singleton CC-NLR from Arabidopsis (Wang *et al*, 2019a, 2019b; preprint: Adachi *et al*, 2022). The activation of ZAR1 upon recognition of its cognate effectors both *in vitro* and *in vivo* leads to its oligomerization and formation of a higher-order pentameric homo-complex analogous to the inflammasome and coined as the resistosome (Wang *et al*, 2019a, 2019b; Hu *et al*, 2020). Oligomerization based activation mechanisms have also been observed *in vivo* for the plant singleton CC-NLR RPP7 and the CC_R-NLR helper NRG1.1 (Li *et al*, 2020; preprint: Jacob *et al*, 2021). More recently, the structure of the activated wheat CC-NLR Sr35 pentameric resistosome suggests that this activation strategy is likely evolutionarily conserved across plant CC-NLRs (preprint: Förderer *et al*, 2022). The activated complexes of CC-NLRs and CC_R-NLRs act as Ca^{2+}-permeable membrane-associated pores upon complex formation, an activity that is required for the hypersensitive cell death (Bi *et al*, 2021; Duggan *et al*, 2021; preprint: Jacob *et al*, 2021; Ngou *et al*, 2021; preprint: Förderer *et al*, 2022). Despite these advances, the molecular mechanisms of paired and networked plant CC-NLR activation are poorly understood. In the case of Pia (RGA4 and RGA5), immune signaling is activated through release of negative regulation (Césari *et al*, 2014). In contrast, the Pik-1 and Pik-2 pair is activated via receptor cooperation by forming a tri-partite complex with the pathogen effector (Zdrzałek *et al*, 2020; De la Concepcion *et al*, 2021). However, whether sensor and helper NLRs engage in heteromeric resistosome complexes is unknown.

Networked NLR immune signaling architectures present many advantages to plant and metazoan immune systems, likely contributing to their robustness and enhancing their evolvability in the face of highly adaptable pathogens (Wu *et al*, 2018). In mammals, multiple different NAIP sensor NLRs can perceive distinct immune elicitors and switch to an active conformation, contributing to immunity. Following activation, NAIPs require the helper NLR NLRC4 to mediate downstream signaling (Kofoed & Vance, 2011; Zhao *et al*, 2011; Vance, 2015). NAIP2 is one of these sensors. Upon perception of its cognate effector, NAIP2 initiates sensor-helper signaling via the formation of a heterocomplex with NLRC4 (Qu *et al*, 2012). This NAIP2/NLRC4 heterocomplex acts as a nucleation point for multiple NLRC4 monomers that leads to the formation of a NAIP/NLRC4 inflammasome with multiple additional NLRC4 units (Hu *et al*, 2015; Zhang *et al*, 2015). Similar networked signaling architectures have also been described in plants. In asterid flowering plants, a major phylogenetic cluster of CC-NLRs known as the NRC superclade comprises an immune receptor network with multiple sensor NLRs and downstream helper NLRs (Wu *et al*, 2017). This network mediates immunity against a wide array of plant pathogens including oomycetes, nematodes, aphids, bacteria and viruses and includes many well characterized and agronomically important sensor NLRs (Wu *et al*, 2017; Derevnina *et al*, 2021). All sensors in the NRC network signal, often redundantly, through a downstream hub of helper NRCs to mediate cell death and disease resistance. NRC helpers contain a key signature in the α-1 helix of their N-termini known as the MADA motif, which is crucial for mediating cell death. This motif is conserved in around 20% of angiosperm CC-NLRs and is functionally conserved between ZAR1, Sr35 and the NRCs, which suggests that the “death switch mechanism” characterized for the ZAR1 resistosome may apply to NRCs as well (Adachi *et al*, 2019a; Kourelis *et al*, 2021a; preprint: Förderer *et al*, 2022). Considering how widespread and vital this immune network is for several crop species, developing a better mechanistic understanding of how it functions is critical. However, how sensor and helper NLR pairs communicate and initiate immune responses is not understood.

In this study, we explore the mechanism of activation of a sensor/helper CC-NLR pair. We selected the NRC-dependent sensor CC-NLR Rx as a model experimental system. Rx is an agronomically important sensor NLR from potato (*Solanum tuberosum)* that confers resistance to *Potato virus X* (PVX), a single-stranded RNA filamentous plant virus, by recognizing its coat protein (CP) (Bendahmane *et al*, 1995, 1999; Grinzato *et al*, 2020). Prior to activation, Rx is held in an inactive state by intramolecular autoinhibitory interactions between its LRR domain and its CC and NB-ARC domains (Moffett *et al*, 2002; Rairdan & Moffett, 2006). Upon CP-triggered activation, Rx undergoes intramolecular rearrangements that include the release of LRR autoinhibition and the exposure of its NB-ARC domain, leading to its activation (Moffett *et al*, 2002). We previously showed that in order to mediate hypersensitive cell death and disease resistance, Rx and other sensors in the NRC network genetically require their downstream NRC helpers, with different sensors exhibiting different NRC helper specificities (Wu *et al*, 2017; Derevnina *et al*, 2021). Rx and the wild pepper (*Capsicum chacoense*) NLR Bs2, for example, can signal interchangeably via NRC2, NRC3 or NRC4. In contrast, the *Solanum bulbocastanum* NLR Rpi-blb2 which confers resistance to *Phytophthora*

 © 2022 The Authors

infestans strains carrying AVRblb2, can only signal through NRC4 (Appendix Fig S1A). However, the mechanisms by which sensor NLRs signal through NRCs are still not understood. Similar to the mammalian NAIP/NLRC4 system, Rx could be forming distinct Rx/NRC higher order hetero-resistosomes with each of its three NRC helpers, reminiscent of the NAIP/NLRC4 inflammasomes (Adachi *et al*, 2019b). Alternatively, plants may feature distinct activation mechanisms for paired and networked NLRs than those previously shown in mammalian paired systems. How activation of sensor NLRs translates into helper activation, immune signaling and disease resistance remains an important question in plant immunology.

To dissect the biochemical mechanisms that underpin Rx and NRC activation, we established a resistosome formation assay using Blue Native polyacrylamide gel electrophoresis (BN-PAGE) by taking advantage of NRC proteins mutated in the MADA motif that are activated without triggering plant cell death. We demonstrate Rx-mediates oligomerization of its NRC2 helper in *Nicotiana benthamiana* following virus perception. Our data suggest that the activated NRC2 complex is an NRC2 resistosome that does not include Rx. Confocal live cell imaging and membrane fractionation assays reveal a sub-cellular shift in localization for NRC2 upon resistosome formation, moving from the cell cytoplasm to the plasma membrane to form membrane-associated punctate structures. This points to an activation-and-release model for sensor-helper signaling in the NRC network, whereby Rx can trigger NRC oligomerization without stably forming part of the activated helper complex. Notably, this model is distinct from the hetero-complexes shown for mammalian NLR paired systems, such as NAIP/NLRC4, implying that plant and metazoan NLR pairs exhibit different activation strategies.

Results

Activation of Rx with *Potato virus X* coat protein leads to oligomerization of its helper NRC2

Prior biochemical *in vivo* studies of activated NLRs have been hampered by the cell death response initiated upon receptor activation. We hypothesized that we could leverage MADA motif mutations in the very N-terminal α1 helix which abolish cell death induction without compromising resistosome formation (Adachi *et al*, 2019a; Hu *et al*, 2020; Duggan *et al*, 2021; preprint: Förderer *et al*, 2022) (Fig 1A). We previously showed that NRC2 is a MADA-type NLR with a conserved motif in its N-terminus, which is required for the execution of cell death and immune signaling. Making mutations in the N-terminal MADA motif of NRC4 completely abolishes NRC4 mediated cell death without affecting protein stability (Adachi *et al*, 2019a; Kourelis *et al*, 2021a). To this end, we created MADA mutations in NRC2 analogous to those previously made in NRC4, to generate an $NRC2^{L9/13/17\ E}$ MADA motif mutant ($NRC2^{EEE}$) (Fig 1A). Like the previously characterized NRC4 mutants, $NRC2^{EEE}$ is unable to functionally complement hypersensitive cell death upon co-expression of effector-activated NRC2-dependent sensors in *nrc2/3/4 N. benthamiana* CRISPR lines (Appendix Fig S1B).

We subsequently leveraged Rx and $NRC2^{EEE}$ to determine the oligomeric state of the sensor and helper upon effector-triggered activation *in vivo*. To achieve this, we transiently expressed these proteins by agroinfiltration in the absence or presence of PVX CP in leaves of *nrc2/3/4 N. benthamiana* CRISPR lines and subjected total protein extracts to BN-PAGE assays (Fig 1B). In its inactive state $NRC2^{EEE}$ appears as a fast-migrating band of ~ 200 kDa, regardless of the presence or absence of Rx (Fig 1C). Upon activating the system by co-expressing Rx and $NRC2^{EEE}$ together with C-terminally tagged CP-GFP, but not upon co-expression of free GFP as a negative control, NRC2 shifts to a slower-migrating, high-molecular-weight complex with two bands in the 720 to 1,048 kDa range (Fig 1C). While we consistently observe this two-band pattern, with a lower molecular weight band of ~ 750 kDa and a higher molecular weight band of ~ 900 kDa, the ~ 900 kDa band is more abundant. This shift to a higher molecular weight complex upon activation of the system is reminiscent of the ZAR1 resistosome formation documented *in vivo* (Hu *et al*, 2020), suggesting that NRC2 may exhibit a similar mechanism of oligomerization and resistosome formation upon activation. The formation of this higher order NRC2 complex is dependent on the presence of Rx, as in the absence of the upstream sensor, co-expression with CP does not result in oligomerization of $NRC2^{EEE}$ or any other apparent change in molecular weight when compared with in the absence of CP (Fig 1C). We conclude that Rx is able and required to mediate NRC2 oligomerization upon CP-triggered activation.

We reproduced the BN-PAGE assays as described above with the wild-type NRC2 carrying an intact N-terminal α1 helix. Because of

Figure 1. Activation of Rx with *Potato virus X* coat protein leads to NRC2 oligomerization.

A Structure of the AtZAR1 resistosome, highlighting the N-terminal α1 helix. Much like AtZAR1 and NRC4, the N-terminal α1 helix encodes a MADA motif. Alignment of NRC2, NRC4 and AtZAR1 N-terminal MADA motifs along with the consensus sequence pattern for the motif and the HMM score for MADA prediction of each sequence. Residues mutated in $NRC2^{EEE}$ mutant are highlighted with red asterisks (positions 9, 13 and 17, respectively).

B Schematic representation of the experimental pipeline used. *Agrobacterium tumefaciens* strains were used to transiently express proteins of interest in leaves of *nrc/2/3/4 Nicotiana benthamiana* CRISPR mutant lines by agroinfiltration. Leaf tissue was harvested 3 days post-infiltration and total protein extracts were used for BN and SDS–PAGE assays.

C BN-PAGE and SDS–PAGE assays with inactive and activated Rx-NRC2. C-terminally 6xHA tagged Rx and C-terminally 4xMyc-tagged $NRC2^{EEE}$ were co-expressed with either free GFP or C-terminally GFP-tagged CP. Free mCherry-4xMyc and mCherry-6xHA fusions were used as controls in the treatments without NRC2 and Rx, respectively. Total protein was extracted with a Tris–HCl-based buffer, as described in materials and methods. Extracts were run on native and denaturing PAGE assays in parallel and immunoblotted with the appropriate antisera labeled on the left. Approximate molecular weights (kDa) of the proteins are shown on the right. Red asterisks indicate bands corresponding to the activated NRC2 complex. Rubisco loading control was carried out using Ponceau stain (PS). The experiment was repeated three times.

Source data are available online for this figure.

© 2022 The Authors

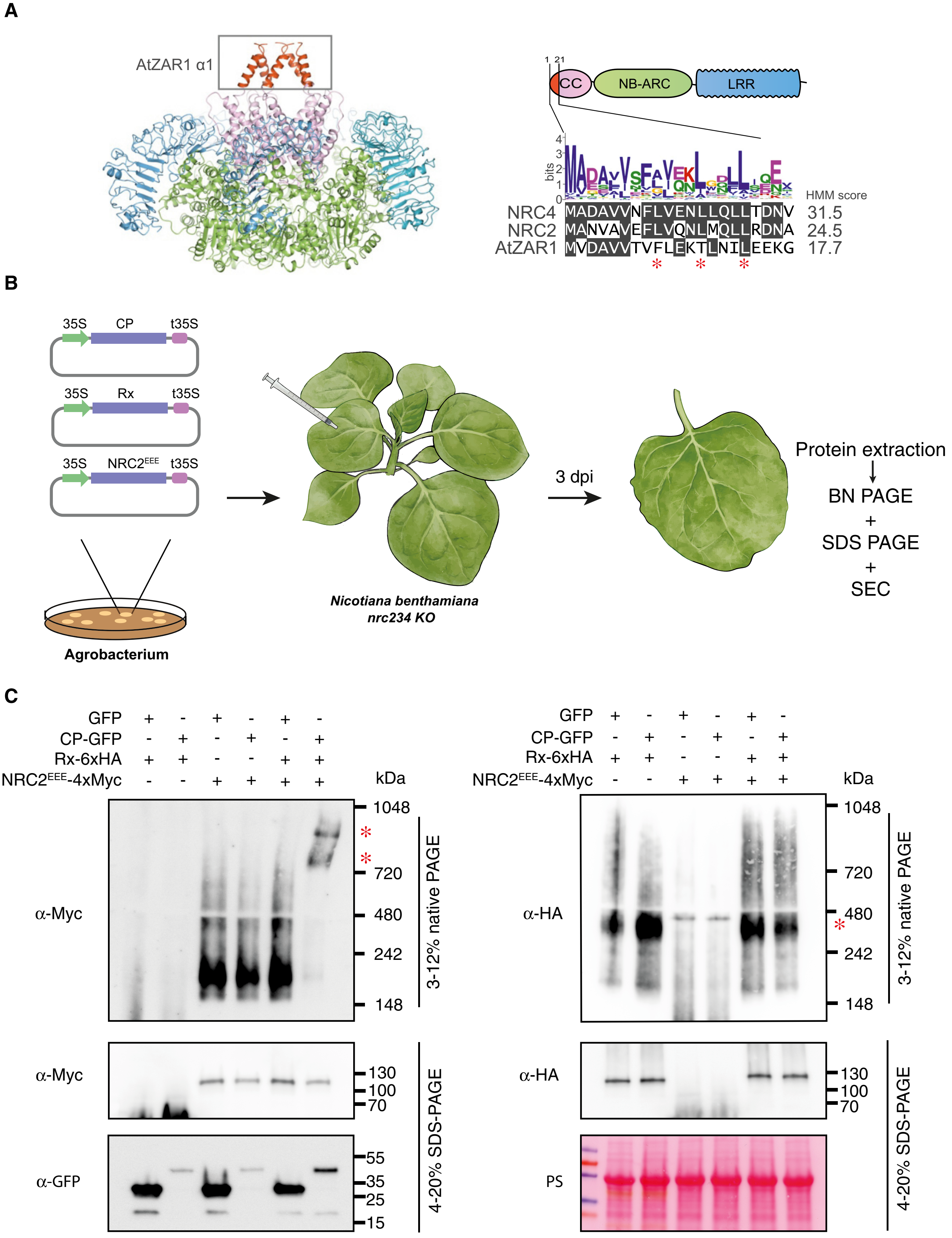

Figure 1.

 © 2022 The Authors

the early onset of cell death triggered upon NRC2 activation, we harvested tissue at the latest timepoint possible before confluent necrosis is observed (38 h post infiltration). Probing for NRC2 in these assays revealed a similar oligomerization pattern to what we observed for NRC2^{EEE} (Fig EV1). However, likely due to the ongoing cell death, protein accumulation of NRC2, Rx and CP was reduced compared with the NRC2^{EEE} treatments. Therefore, we decided to move forward with NRC2^{EEE} for subsequent experiments to mitigate the effects of cell death.

Rx does not oligomerize following activation by CP

In the BN-PAGE assays performed above, we observed that the sensor Rx constitutively migrates as a band of ~ 400 kDa, regardless of its activation state (Figs 1B and EV1). Interestingly, the presence of this ~ 400 kDa band does not depend on the presence of NRC2^{EEE} in *N. benthamiana nrc2/3/4* CRISPR lines, suggesting that it is unlikely to be a preformed complex between Rx or any endogenous NRC2, NRC3 or NRC4. Considering that the size of a single Rx-6xHA monomer is expected to be ~ 115 kDa, we hypothesized that the ~ 400 kDa band observed for Rx in BN-PAGE assays could correspond to a preformed Rx complex with additional host proteins, such as RanGAP2, as has been previously reported (Tameling & Baulcombe, 2007). Rx could also potentially constitutively self-associate, forming a larger complex of multiple sensor units. We did not observe any shifts in size for Rx upon activation of the system with CP (Fig 1C). This is in line with previous reports that CP and Rx do not form a stable complex (Tameling & Baulcombe, 2007). Furthermore, we were unable to detect any signal for Rx at a size matching the activated higher molecular weight NRC2^{EEE} complex described above, suggesting that Rx does not form a stable part of this activated NRC2 complex (Fig 1C). This implies an activation mechanism that differs from the hetero-oligomeric inflammasome previously reported for mammalian paired NLRs.

To independently validate the BN-PAGE experiments using a different analytical method, we performed gel filtration assays on protein extracts from leaves of *nrc2/3/4 N. benthamiana* CRISPR lines expressing Rx and NRC2^{EEE} in the absence or presence of PVX CP. Proteins of interest were detected in the resulting fractions by SDS–PAGE analysis and immunoblotting with corresponding antibodies. Inactive NRC2^{EEE} migrated mostly as a small molecular mass complex, eluting at around 15 mL, while Rx migrated at a slightly higher molecular weight, with most signal eluting at 13 mL (Fig EV2). Consistent with the BN-PAGE assays, upon activation of the system by co-expressing CP-GFP, almost all NRC2^{EEE} exhibited a shift in elution volume, migrating as a large complex. This complex eluted at a size between ~ 440 kDa and ~ 669 kDa, which would be consistent with a pentameric NRC2^{EEE} resistosome complex (~ 550 kDa) (Fig EV2). Rx did not exhibit a shift in elution volume upon activation. We also did not observe an increase in signal for Rx in any of the fractions corresponding to the activated NRC2^{EEE} complex. Our results in both BN-PAGE and gel filtration assays suggest that upon effector perception, Rx can mediate oligomerization of NRC2 into a higher order complex of ~ 550 kDa without stably associating with this complex. As both techniques yielded similar results and given that gel filtration required long sample handling times, we decided to use BN-PAGE as a readout for the remainder of the study.

Rx does not form a stable complex with NRC2

To further test the hypothesis that Rx does not form part of the activated NRC2 complex, we decided to use different molecular weight tags to study Rx-NRC2 interactions with size shifts. In previous experiments, we employed C-terminally tagged Rx-6xHA and NRC2^{EEE}-4xMyc (from here onwards termed "light" versions) (Fig 2A). We then generated C-terminal tandem tag constructs to generate Rx-mCherry-6xHA and NRC2^{EEE}-mCherry-4xMyc (termed "heavy" versions) (Fig 2A). We used these higher molecular weight versions to determine whether the addition of a larger molecular weight tag in one of the Rx-NRC2 system components could induce a shift in size of the other in BN-PAGE assays. We first confirmed that these new "heavy" versions retained the ability to correctly mediate hypersensitive cell death (Appendix Fig S2). When complementing leaves of *nrc2/3/4 N. benthamiana* CRISPR mutant lines with light NRC2^{EEE}-4xMyc together with the heavy Rx-mCherry-6xHA, BN-PAGE assays showed no shift in size for NRC2^{EEE}, either in the inactive or activated states, relative to light NRC2^{EEE}-4xMyc co-expressed with light Rx-6xHA (Fig 2B). Heavy Rx-mCherry-6xHA, in contrast, exhibited a shift in size both on BN-PAGE and SDS–PAGE (Fig 2B). Similarly, co-expression of heavy NRC2^{EEE}-mCherry-4xMyc with light Rx-6xHA does not result in a size-shift for Rx in BN-PAGE relative to light NRC2^{EEE}-4xMyc co-expressed with light Rx-6xHA. Again, a clear shift in size was observed in both the inactive and activated states for heavy NRC2^{EEE}-mCherry-4xMyc relative to light NRC2^{EEE}-4xMyc both in BN-PAGE and SDS–PAGE (Fig 2B). All in all, these data suggest that Rx and NRC2 likely do not form stable complexes with each other at resting state, and that following activation by its upstream sensor, NRC2 oligomerizes and forms a higher order complex that does not include Rx.

The activated NRC2 complex is composed of multiple NRC2 proteins

Considering that our findings are in line with the hypothesis that Rx does not form part of the active NRC2 complex, we next sought to further characterize the nature of this high-molecular-weight NRC2 complex. To do so, we leveraged the previously used heavy and light tag approach. We postulated that if NRC2 monomers were indeed forming a complex upon activation, a heterogeneous mixture of differently sized NRC2 molecules would lead to a shift in size of the activated complex, relative to homogeneous heavy or light NRC2 complexes. To test this hypothesis, we co-expressed Rx-6xHA with either light NRC2^{EEE}-3xFLAG (104 kDa), heavy NRC2^{EEE}-mCherry-4xMyc (133 kDa) or a mixture of heavy and light NRC2 variants in leaves of *nrc2/3/4 N. benthamiana* CRISPR mutant lines (Fig 3A). As expected, both the inactive and activated heavy NRC2^{EEE}-mCherry-4xMyc exhibited a higher molecular weight than the inactive and activated light NRC2^{EEE}-3xFLAG complexes, respectively (Fig 3B). However, upon co-expressing both heavy NRC2^{EEE}-mCherry-4xMyc and light NRC2^{EEE}-3xFLAG, the activated NRC2 complex was of intermediate molecular weight, relative to either light NRC2^{EEE}-3xFLAG or heavy NRC2-mCherry-4xMyc complexes (Fig 3B). The observation that combining differently sized NRC2 variants results in an intermediate molecular weight activated complex suggests that upon activation by Rx, both NRC2 versions are entering a complex. Interestingly, we were unable to see any change in size for inactive NRC2^{EEE} when mixing the two molecular weight variants (Fig 3B).

© 2022 The Authors

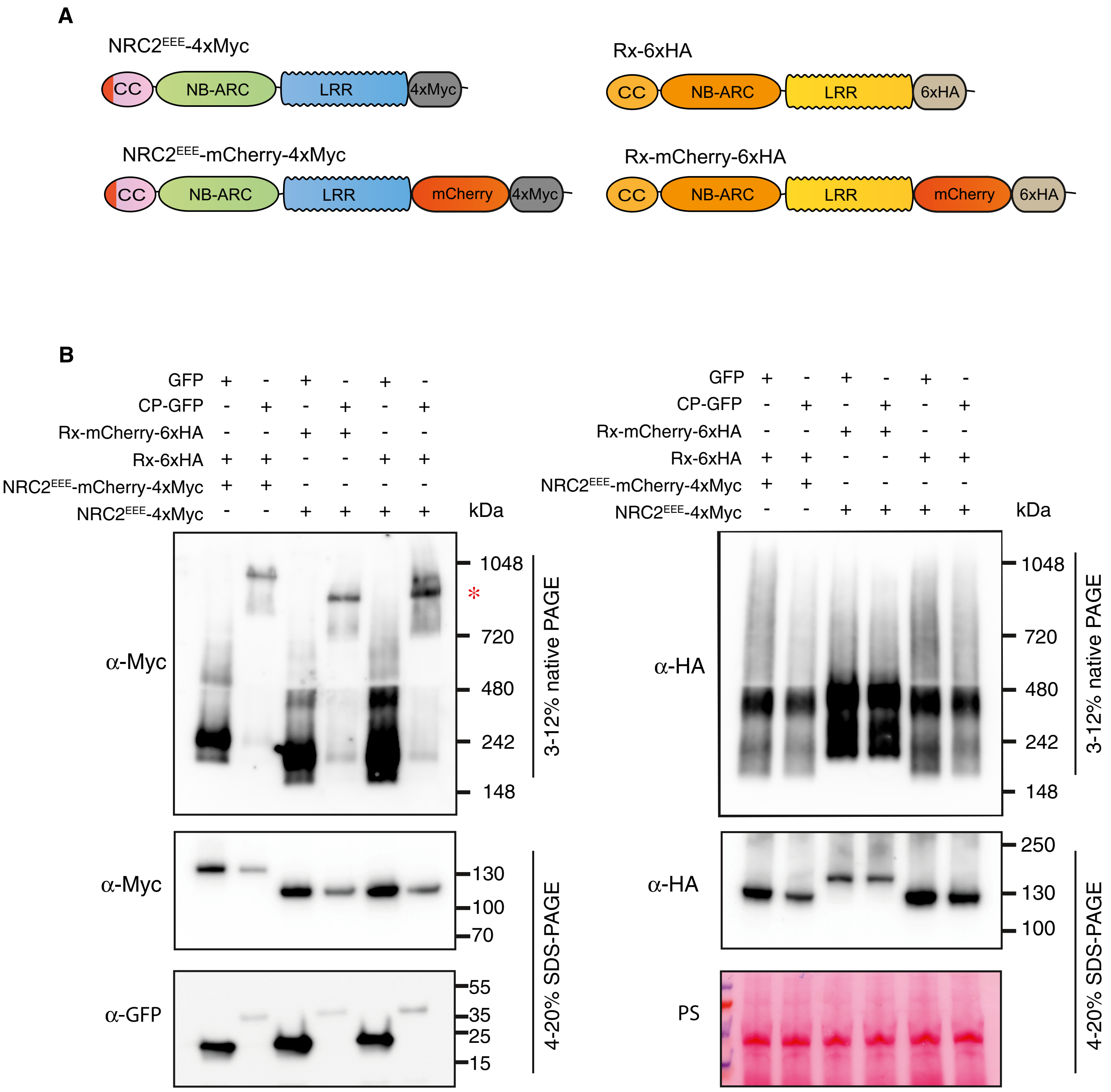

Figure 2. Rx and NRC2 do not form a stable complex upon activation.

A Schematic representation of NRC2^{EEE} and Rx with differently sized tags. The C-terminally 4xMyc-tagged NRC2^{EEE} and C-terminally 6xHA tagged Rx used in previous experiments were termed "light" versions, while the new C-terminally tandem mCherry-4xMyc-tagged NRC2^{EEE} and C-terminally mCherry-6xHA-tagged Rx variants were termed "heavy" versions.

B BN-PAGE and SDS–PAGE assays with inactive and activated Rx-NRC2 using different heavy and light sensor-helper combinations. Total protein was extracted with a Tris–HCl-based buffer, as described in materials and methods. Extracts were run on native and denaturing PAGE assays in parallel and immunoblotted with the appropriate antisera labeled on the left. Approximate molecular weights (kDa) of the proteins are shown on the right. Red asterisk indicates bands corresponding to the activated NRC2 complex. Rubisco loading control was carried out using Ponceau stain (PS). The experiment was repeated three times.

Source data are available online for this figure.

We conclude that the fast-migrating lower molecular weight band observed for inactive NRC2 is likely not a complex of multiple NRC2 molecules. This allows us to draw a model for Rx-NRC2 activation in which, following effector-triggered activation, Rx leads to activation of its downstream helper NRC2, which oligomerizes and forms a resistosome complex that includes multiple NRC2 units.

© 2022 The Authors

Bs2, another NRC2-dependent sensor NLR, also triggers oligomerization of NRC2

Since Rx is able to trigger oligomerization of the downstream helper NRC2, we tested whether this applies to other NRC-dependent sensors. We performed complementation assays co-expressing NRC2^{EEE} in leaves of *nrc2/3/4 N. benthamiana* CRISPR mutants along with the inactive or activated NRC2/3/4-dependent sensor Bs2, or Rpi-blb2, an NRC4-dependent sensor that is unable to signal through NRC2 (Fig 4A) (Wu *et al*, 2017; Duggan *et al*, 2021). In addition, all three C-terminally 6xHA-tagged sensors were tested in cell death assays to ensure that tagging did not interfere with effector-triggered

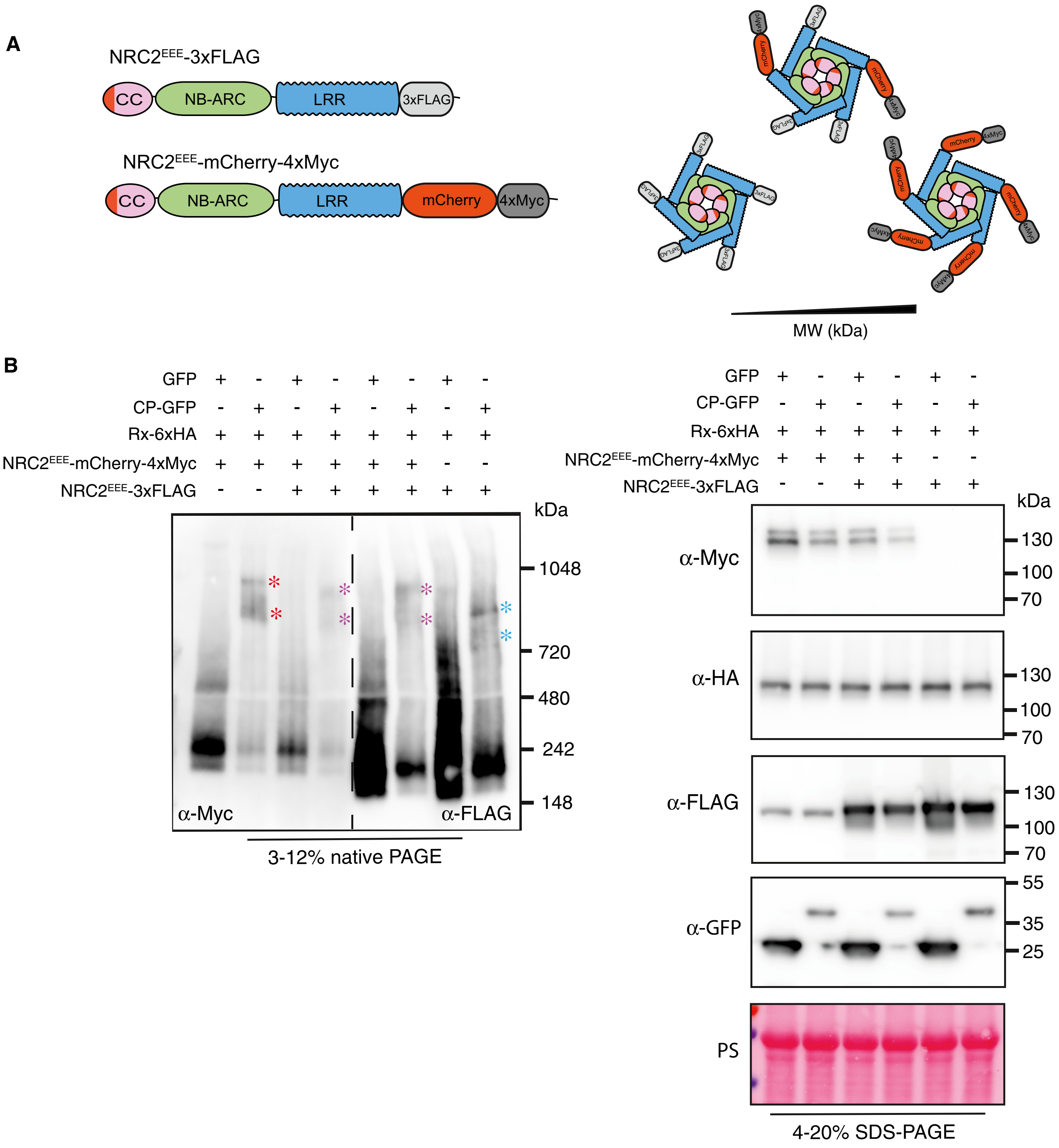

Figure 3.

Figure 3. The activated NRC2^{EEE} complex is composed of multiple NRC2 units.

A Schematic representation of NRC2^{EEE} with differently sized tags. The C-terminally 3xFLAG-tagged NRC2^{EEE} was termed the "light" version and C-terminally mCherry-4xMyc tagged NRC2^{EEE} was termed the "heavy" variant. If NRC2 forms homo-oligomers, active complexes with different proportions of "heavy" and "light" NRC2 monomers should in theory exhibit different molecular weights.

B BN-PAGE and SDS–PAGE assays with inactive and activated Rx-NRC2 using different "heavy" and "light" helper combinations. Total protein was extracted with a Tris–HCl-based buffer, as described in materials and methods. Extracts were run on native and denaturing PAGE assays in parallel and immunoblotted with the appropriate antisera labeled on the left. Approximate molecular weights (kDa) of the proteins are shown on the right. A non-specific band was observed at ~ 110 kDa for the anti-FLAG antibody. Red asterisks indicate bands corresponding to the activated "heavy" NRC2 complexes. Blue asterisks indicate bands corresponding to "light" NRC2 complexes. Purple bands indicate intermediate molecular weight complexes combining "heavy" and "light" NRC2. Rubisco loading control was carried out using Ponceau stain (PS). The experiment was repeated three times.

Source data are available online for this figure.

signaling (Appendix Fig S3). Our BN-PAGE assays revealed that while Bs2 activation with AvrBs2 was able to trigger NRC2^{EEE} resistosome formation in a similar fashion to Rx, Rpi-blb2 activation with AVRblb2 did not lead to the formation of a NRC2^{EEE} resistosome (Fig 4B). This suggests that sensor-mediated NRC2 oligomerization applies to another NRC2 dependent sensor. Moreover, the observation that only sensors that genetically require NRC2 can trigger its oligomerization shows that previously genetically characterized sensor-helper specificities in the NRC network can be recapitulated biochemically by helper oligomerization in BN-PAGE.

Bs2, Rx and Rpi-blb2 can trigger oligomerization of their helper NRC4

We next tested whether the NRC2 activation mechanism also applies to another NRC helper, NRC4, which can support signaling by the three previously tested sensors, Bs2, Rx and Rpi-blb2 (Fig 4A). We leveraged a previously published NRC4 variant with L9A/V10A/L14A mutations in its N-terminal MADA motif (NRC4^{AAA}) (Adachi *et al*, 2019a) that does not cause hypersensitive cell death and enables *in planta* biochemical analyses. We performed complementation assays by co-expressing NRC4^{AAA} in leaves of *nrc2/3/4 N. benthamiana* CRISPR mutants along with the inactive or effector-activated sensors mentioned previously. All three sensor NLRs were able to trigger NRC4^{AAA} oligomerization (Fig 5). This suggests that sensor-mediated NRC helper oligomerization upon effector perception applies to another helper NLR in the NRC network.

Activated NRC2 oligomers accumulate in membrane-associated puncta, whereas Rx remains cytoplasmic

The cellular biology of activated NLRs remains poorly understood. We previously showed that the NRC helper NLR NRC4 exhibits dynamic spatiotemporal changes in subcellular localization following effector-triggered activation of its upstream sensor NLR Rpi-blb2 (Duggan *et al*, 2021). We applied similar methods to study the sub-cellular dynamics of the Rx-NRC2 system by transiently co-expressing fluorescently tagged versions of Rx-RFP and NRC2^{EEE}-GFP in leaves of *nrc2/3/4 N. benthamiana* CRISPR mutants. We activated the Rx-NRC2 system by expressing 4xMyc-tagged CP or a 4xMyc-tag empty vector (EV) control and monitored sensor and helper localization using confocal live-cell imaging (Fig 6). As a plasma membrane (PM) marker, we co-expressed RPW8.2-BFP (Duggan *et al*, 2021). In parallel, protein was extracted from the same leaf tissue used for microscopy to confirm that the tags do not interfere with Rx-mediated NRC2 cell death and oligomerization by BN-PAGE assays (Appendix Fig S4). In their inactive state, both Rx-RFP and NRC2^{EEE}-GFP co-localize to the cytoplasm in 100% of observations ($N = 16$ images) (Fig 6A; Movie EV1). Strikingly, when co-expressing CP, activated NRC2^{EEE}-GFP predominantly localizes to puncta which frequently co-localize with the PM, marked by RPW8.2-BFP. In contrast, Rx-RFP does not exhibit major changes in subcellular localization. The sensor remains in the cytoplasm and does not concentrate in the NRC2 puncta (15/16 images taken). These puncta are uniformly distributed throughout the PM (Fig 6B; Movie EV1). To investigate the membrane association of the activated oligomeric NRC2 complex, we obtained protein extracts from the same tissues used for microscopy and carried out membrane fractionation assays in nondenaturing conditions using the same experimental setup described above and performed SDS–PAGE assays using the different fractions (Fig 6C). In line with what we previously observed in live-cell imaging experiments, we found that in the inactive state, both Rx and NRC2^{EEE} are mainly present in the soluble fraction. Following activation, NRC2^{EEE} is equally distributed between the soluble and membrane fractions, indicating a shift in subcellular localization and increased membrane-association. Rx, however, exhibits no such shift upon activation and remains predominantly in the soluble fraction. We conclude that upon effector-triggered activation of Rx, the sensor subsequently mediates activation of its helper NRC2^{EEE} in the cytoplasm. The activated NRC2 units form oligomeric resistosomes that dynamically relocalize and form membrane-associated puncta that are separate from the sensor.

Figure 4. Bs2, another NRC2-dependent sensor NLR, triggers oligomerization of NRC2^{EEE}.

A Schematic representation of the NRC genetic dependencies of sensors used in this experiment.

B BN-PAGE and SDS–PAGE assays with inactive and activated NRC-dependent sensors and NRC2. Total protein was extracted with a Tris–HCl-based buffer, as described in materials and methods. Extracts were run on native and denaturing PAGE assays in parallel and immunoblotted with the appropriate antisera labeled on the left. Approximate molecular weights (kDa) of the proteins are shown on the right. Red asterisk indicates bands corresponding to the activated NRC2 complexes. Rubisco loading control was carried out using Ponceau stain (PS). The experiment was repeated three times.

Source data are available online for this figure.

 © 2022 The Authors

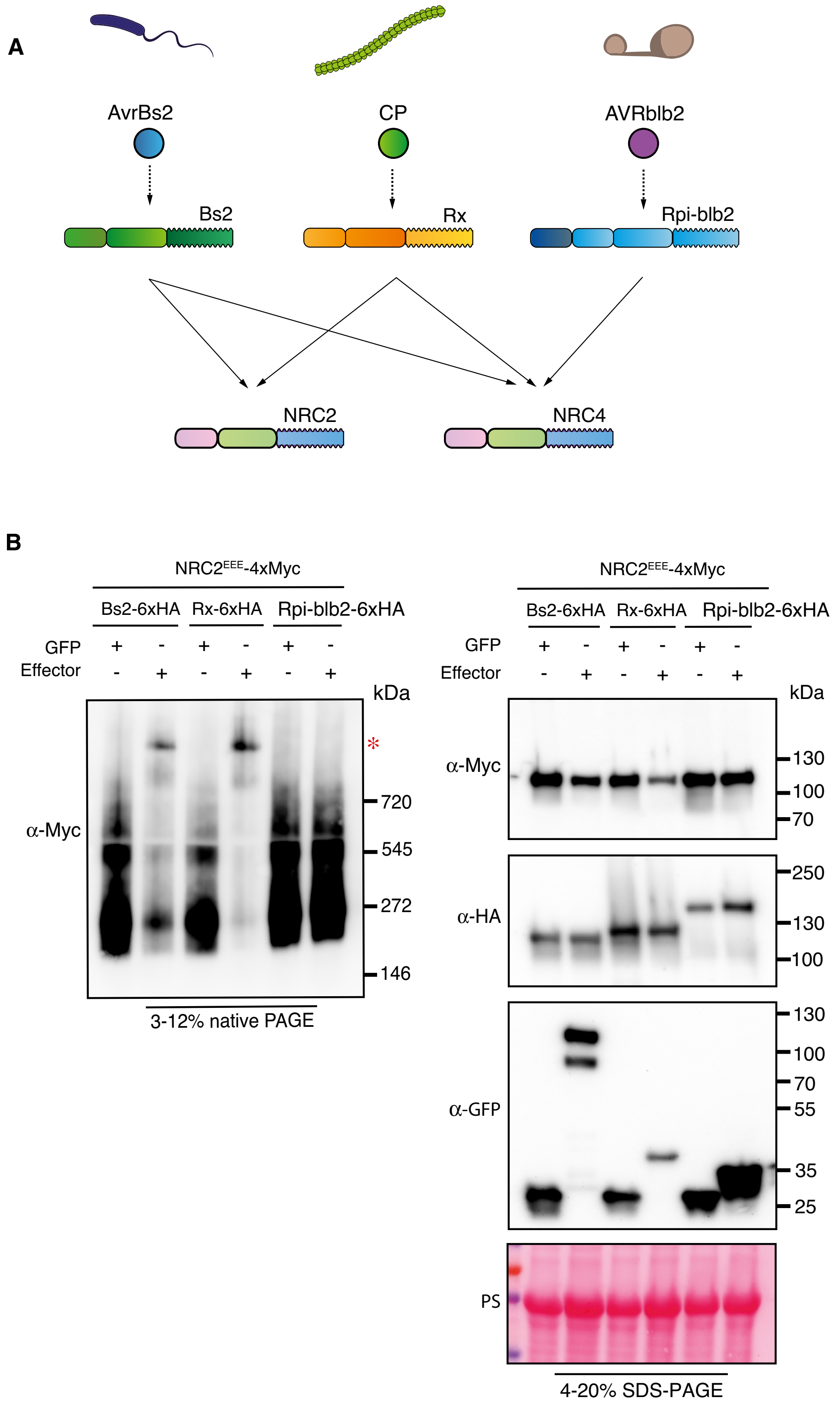
A
AvrBs2
CP
AVRblb2
Bs2
Rx
Rpi-blb2
NRC2
NRC4
B
NRC2EEE-4xMyc
Bs2-6xHA
Rx-6xHA
Rpi-blb2-6xHA
GFP
Effector
kDa
α-Myc
720
545
272
146
3-12% native PAGE
α-Myc
130
100
70
α-HA
250
130
100
α-GFP
130
100
70
55
35
25
PS
4-20% SDS-PAGE

Figure 4.

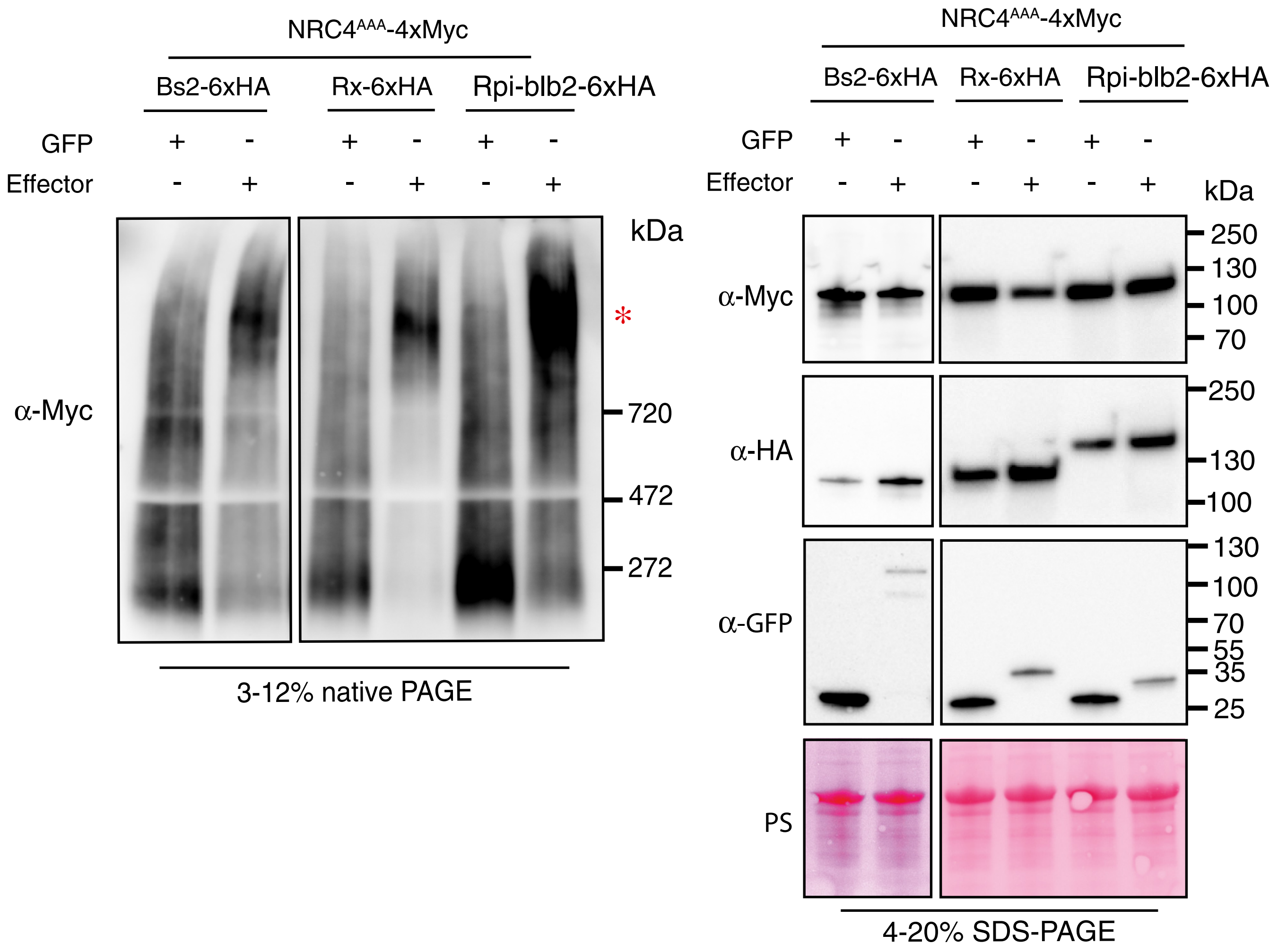

Figure 5. Bs2, Rx and Rpi-blb2 trigger oligomerization of another helper NLR, NRC4.

BN-PAGE and SDS–PAGE assays with inactive and activated NRC-dependent sensors and NRC4^{AAA}. Total protein was extracted with a HEPES-based buffer, as described in materials and methods. Extracts were run on native and denaturing PAGE assays in parallel and immunoblotted with the appropriate antisera labeled on the left. Approximate molecular weights (kDa) of the proteins are shown on the right. Red asterisk indicates bands corresponding to the activated NRC4 complexes. Rubisco loading control was carried out using Ponceau stain (PS). The experiment was repeated three times.
Source data are available online for this figure.

Potato virus X infection triggers Rx-mediated oligomerization of NRC2

To confirm our previous results in the context of pathogen infection rather than with activation using heterologously expressed effectors, we used BN-PAGE to study the activation of NRC2 in the context of viral infection. We transiently co-expressed Rx and NRC2^{EEE} in leaves of *nrc2/3/4 N. benthamiana* CRISPR mutant lines and activated the system by infecting leaf tissues with PVX via co-expressing a GFP tagged PVX variant (pGR106::PVX::GFP), as

Figure 6. *Potato virus X* coat protein activated NRC2 forms plasma membrane-associated puncta, unlike Rx. ▶

C-terminally GFP-tagged NRC2^{EEE} and C-terminally RFP-tagged Rx were co-expressed either with an EV-4xMyc construct or a CP-4xMyc construct in leaves of *nrc2/3/4 N. benthamiana* CRISPR mutant lines.

A, B Single-plane confocal micrographs show the localization of both components of the inactive and active Rx-NRC2 system, together with PM marker RPW8.2-BFP. Scale bars represent 10 μm. (A) NRC2^{EEE}-GFP and Rx-RFP co-localize in the cytoplasm prior to activation. (B) Upon co-expression of CP and activation of the system, NRC2^{EEE} forms puncta associated with the PM while Rx remains in the cytoplasm.

C Membrane enrichment assays are consistent with microscopy, showing that inactive NRC2^{EEE}-GFP is mostly present in the soluble (cytoplasmic) fraction, whereas activated NRC2^{EEE}-GFP exhibits equal distribution across soluble and membrane fractions. Rx is mostly present in the soluble fraction and exhibits no change upon activation of the system with CP. T = total, S = soluble, M = membrane. ATPase was used as a membrane marker. Rubisco was used as a marker for total and soluble fractions and visualized by Ponceau staining (PS). Red asterisks indicate bands matching the expected MW for each protein. The experiment was repeated two times.

Source data are available online for this figure.

 © 2022 The Authors

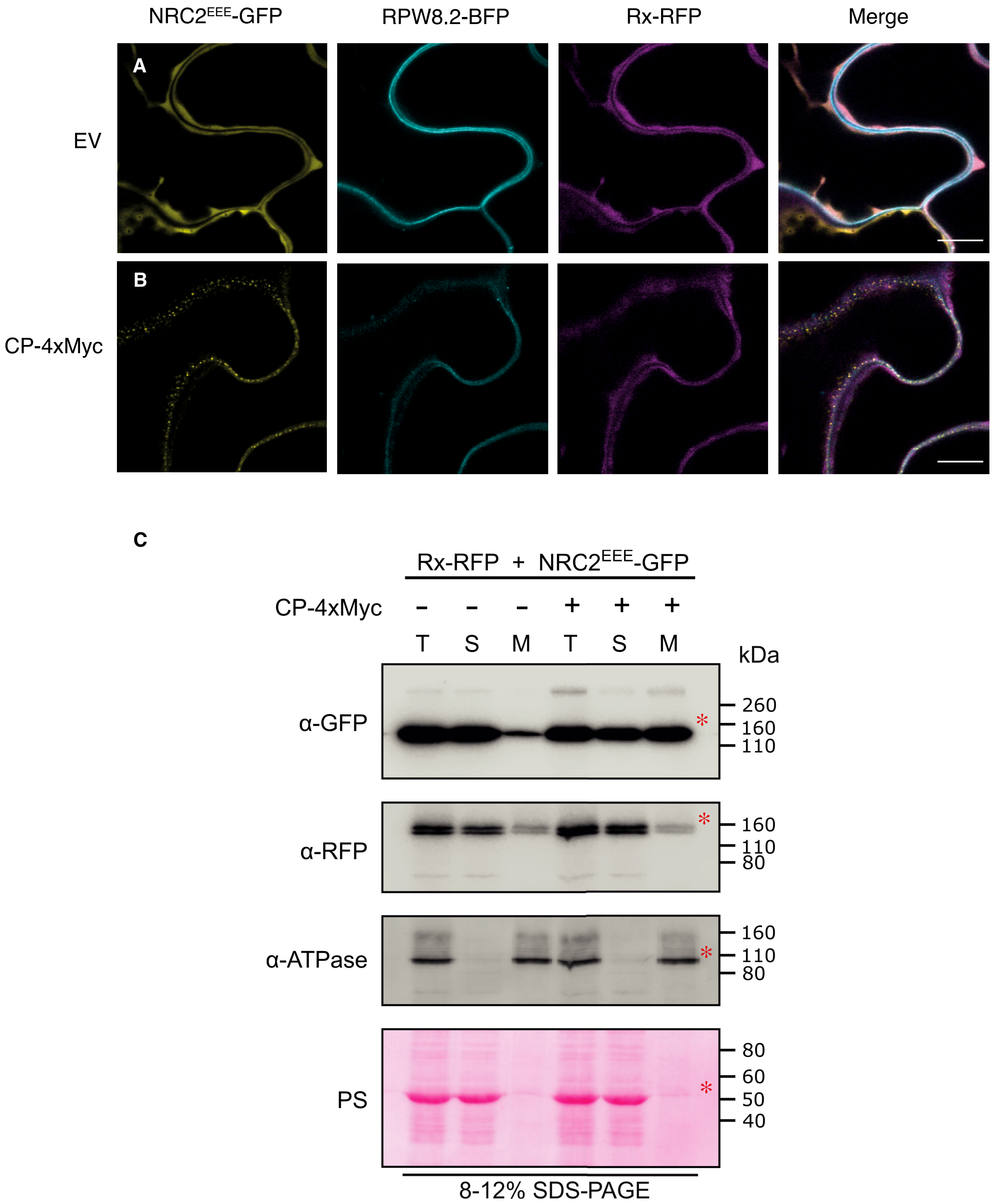
NRC2EEE-GFP
RPW8.2-BFP
Rx-RFP
Merge
A
EV
B
CP-4xMyc
C
Rx-RFP + NRC2EEE-GFP
CP-4xMyc - - - + + +
T S M T S M
kDa
α-GFP
260
160
110
α-RFP
160
110
80
α-ATPase
160
110
80
PS
80
60
50
40
8-12% SDS-PAGE

Figure 6.

described previously (Derevnina *et al*, 2021). Transient expression of free GFP was used as a negative control for infection. After 3 days, protein extracts from infected or uninfected leaf tissues were used for BN-PAGE assays (Fig 7A). Consistent with previous results, infection with PVX led to sensor-dependent oligomerization of NRC2^{EEE} (Fig 7B). Interestingly, in the SDS–PAGE, a very strong GFP signal was observed in all PVX treatments, suggesting that the virus is able to replicate in the presence of Rx and NRC2^{EEE}. This implies that, while the leucine to glutamic acid mutations in the N-terminal MADA motif of NRC2 do not prevent resistosome formation mediated by Rx, the NRC2^{EEE} mutant is both unable to trigger hypersensitive cell death and mediate Rx-dependent PVX resistance (Fig 7B). We conclude that for sensor-helper pairs in the NRC network, the sensor NLR is activated following pathogen recognition and subsequently signals to its downstream NRC helper, leading to its oligomerization and resistosome formation. We also conclude that an NRC helper with a functional MADA-motif is necessary for the Rx-NRC2 system to mediate full PVX resistance.

Potato virus X infection triggers Rx-dependent formation of membrane-associated NRC2 puncta

We next sought out to study the sensor-dependent subcellular reorganization of NRC2 described above in the context of pathogen infection. To this end, we transiently co-expressed NRC2^{EEE} either with Rx-RFP or free RFP in leaves of *nrc2/3/4 N. benthamiana* CRISPR mutant lines and activated the system by infecting leaf tissues with PVX (pGR106::PVX), as described previously (Derevnina *et al*, 2021). After 3 days, we monitored sensor and helper localization using confocal live-cell imaging (Fig 8). Under these infection conditions, the helper NRC2^{EEE}-GFP remains in the cytoplasm in the absence of its upstream sensor Rx (18/18 images taken) (Fig 8A). Consistent with previous results, when co-expressing NRC2^{EEE}-GFP together with Rx-RFP and infecting with PVX, the helper predominantly localizes to PM-associated puncta. In contrast, Rx remains in the cytoplasm during PVX infection and does not exhibit co-localization with NRC2 (18/18 images taken) (Fig 8B). Our data indicate that the dynamic re-localization and PM-association of NRC2 we observed following treatment with CP (Fig 6) also occurs during pathogen infection.

Discussion

The aim of this study was to develop a better understanding of the molecular mechanisms that underpin activation of paired and networked plant NLR immune receptors. Such *in planta* studies were previously hampered by the cell death response associated with activated NLRs. The capacity to make mutations in the N-terminal MADA motif of CC-NLR helper proteins that abolish elicitation of cell death without affecting activation enabled us to develop readouts for the formation of resistosome-like oligomers and address several questions related to NRC activation. We used biochemical and cellular biology techniques to develop a working model for activation of the NRC network sensors NLRs Rx, Bs2, Rpi-blb2 and their helpers NRC2 and NRC4 (Wu *et al*, 2017; Derevnina *et al*, 2021). Our data demonstrate that these sensor NLRs can mediate NRC oligomerization (Figs 1, 4, 5, and EV1) upon activation with their corresponding effectors and in the case of Rx, during pathogen infection (Fig 7). For NRC2, we show that sensor activation also mediates helper re-localization to PM during pathogen infection (Figs 6 and 8). This activated NRC2 complex appears to be an oligomer containing multiple NRC2 units, which excludes the sensor NLR (Figs 1, 2, 3, and EV2), suggesting a model for sensor-helper activation that differs from mammalian paired NLR systems, such as NAIP/NLRC4 (Vance, 2015; Zhang *et al*, 2015). These findings and those of a co-published study on oligomerization of NRC2 after activation of the oomycete resistance proteins Rpi-amr1 and Rpi-amr3 (preprint: Ahn *et al*, 2022) have led us to develop an activation-and-release working model for NLRs in the NRC network (Fig 9).

The N-terminal α1-helices of ZAR1, Sr35 and the NRCs are defined by a molecular signature called the “MADA motif” (Adachi *et al*, 2019a; Kourelis *et al*, 2021a). This motif can be found in ~ 20% of angiosperm CC-NLRs and is essential for immune signaling and disease resistance. For TIR-NLR biology, multiple essential downstream components for immune signaling, such as the EDS1 signaling hub and the CC_R NLRs NRG1 and ADR1 have been identified, which enabled the generation of genetic backgrounds with which to study TIR-NLR activities in the absence of immune signaling (preprint: Gantner *et al*, 2019; Lapin *et al*, 2019; Martin *et al*, 2020; Saile *et al*, 2020; Sun *et al*, 2021). Unlike TIR-NLRs, approaches to study activated CC-NLRs in the absence of a plant cell death response were not available until recently (Adachi *et al*, 2019a; Duggan *et al*, 2021). The availability of MADA mutant variants that no longer trigger cell death, but retain the capacity to activate, oligomerize and re-localize enabled us to study the cellular and biochemical mechanisms of CC-NLR activation.

MADA motif mutants of NRCs are unable to mediate cell death yet they can still oligomerize and relocalize to the PM upon activation by their upstream sensors. How does this fit with current models of CC-NLR execution of the hypersensitive cell death? Our work complements existing studies that show how MADA mutants can be leveraged to shed light on the diversity of molecular mechanisms that underpin CC-NLR activation (Adachi *et al*, 2019a; Hu *et al*, 2020; preprint: Kourelis *et al*, 2021a; preprint: Förderer *et al*, 2022). Two studies on NRC4 and ZAR1, respectively, showed that mutations in the N-terminal MADA-motif containing α1-helices abolished cell death triggered by these NLRs but had no effect on PM localization (Hu *et al*, 2020; Duggan *et al*, 2021). Similar to NRC2^{EEE} and NRC4^{AAA}, ZAR1 and Sr35 with mutated N-termini were still able to form resistosomes (Hu *et al*, 2020; preprint: Förderer *et al*, 2022). Resistosome assembly and PM localization are, therefore, required but not sufficient to mediate cell death. In the case of Sr35, mutations in the MADA motif even abolished the capacity of this NLR to trigger cell death in insect cells (preprint: Förderer *et al*, 2022), suggesting that no further downstream components are required or that the signaling initiated by these resistosomes can engage with highly conserved pathways present across the plant and animal kingdoms. How do MADA motif mutations interfere with cell death? One possibility is that the MADA mutations interfere with the Ca^{2+} channel activity that was recently assigned to activated CC and CC_R-NLR proteins (Bi *et al*, 2021; preprint: Jacob *et al*, 2021; preprint: Förderer *et al*, 2022). Alternatively, N-terminal α1-helices in CC-NLRs with mutated MADA motifs may result in resistosomes that associate with the plasma membrane but are unable to fully penetrate the lipid bilayer to form a functional pore or channel. Further

 © 2022 The Authors

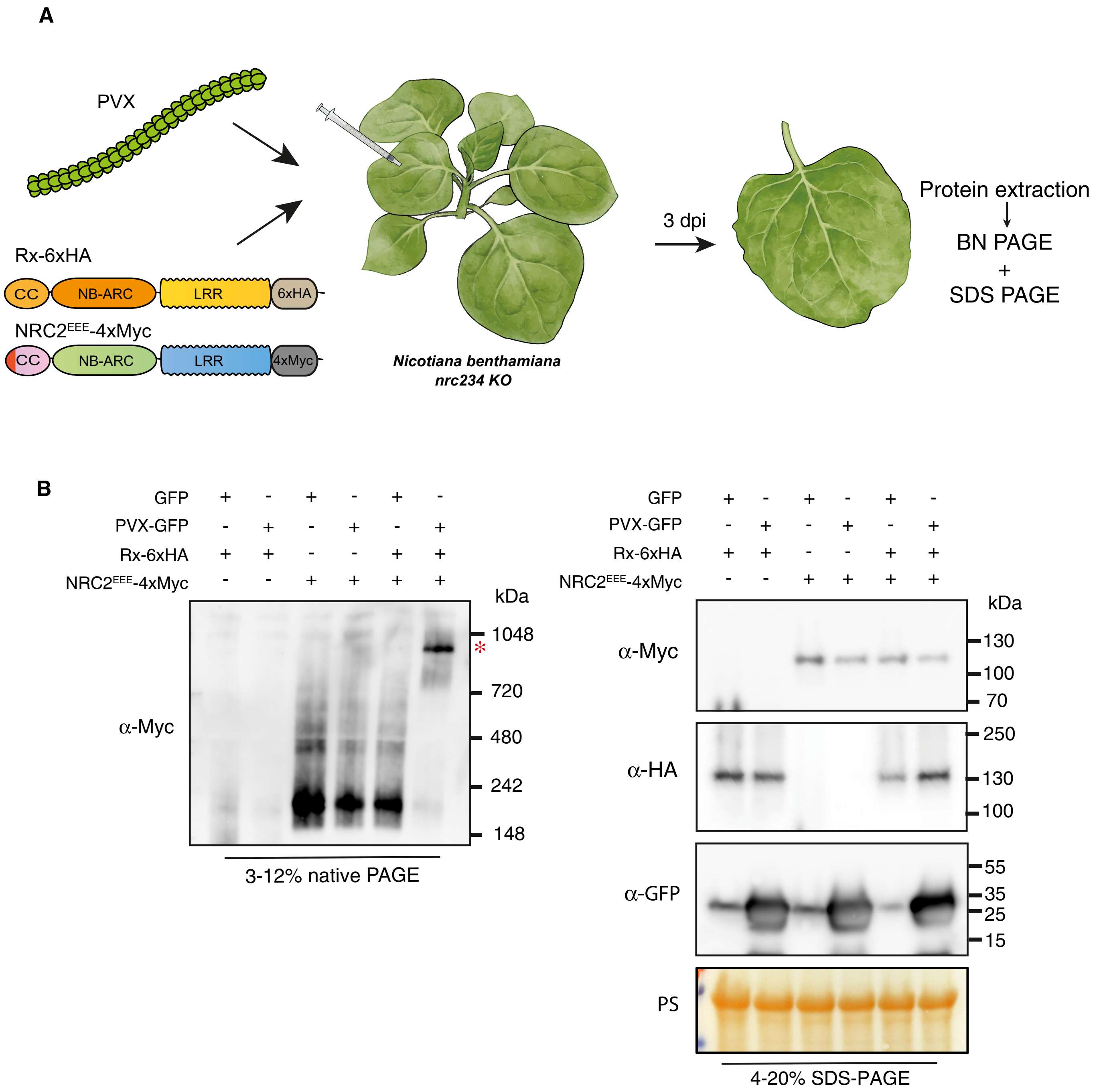

Figure 7. ***Potato virus X*** **infection triggers Rx-mediated oligomerization of NRC2.**

A Schematic representation of the experimental pipeline used. *A. tumefaciens* strains were used to transiently express proteins of interest in leaves of *nrc/2/3/4 N. benthamiana* CRISPR mutant lines by agroinfiltration. Simultaneously, the same leaves were infected with PVX by using an *A. tumefaciens* carrying GFP expressing PVX. Free GFP was used as a negative control for PVX-GFP infection. Free mCherry-4xMyc and mCherry-6xHA fusions were used as controls in the treatments without NRC2 and Rx, respectively. Leaf tissue was harvested 3 days post-infiltration and total protein extracts were used for BN and SDS–PAGE assays.

B BN-PAGE and SDS–PAGE assays with infected and uninfected leaves expressing Rx and NRC2. Total protein was extracted with a Tris–HCl-based buffer, as described in materials and methods. Extracts were run on native and denaturing PAGE assays in parallel and immunoblotted with the appropriate antisera labeled on the left. Approximate molecular weights (kDa) of the proteins are shown on the right. Red asterisks indicate bands corresponding to the activated NRC2 complexes. Rubisco loading control was carried out using Ponceau stain (PS). The experiment was repeated three times.

Source data are available online for this figure.

© 2022 The Authors

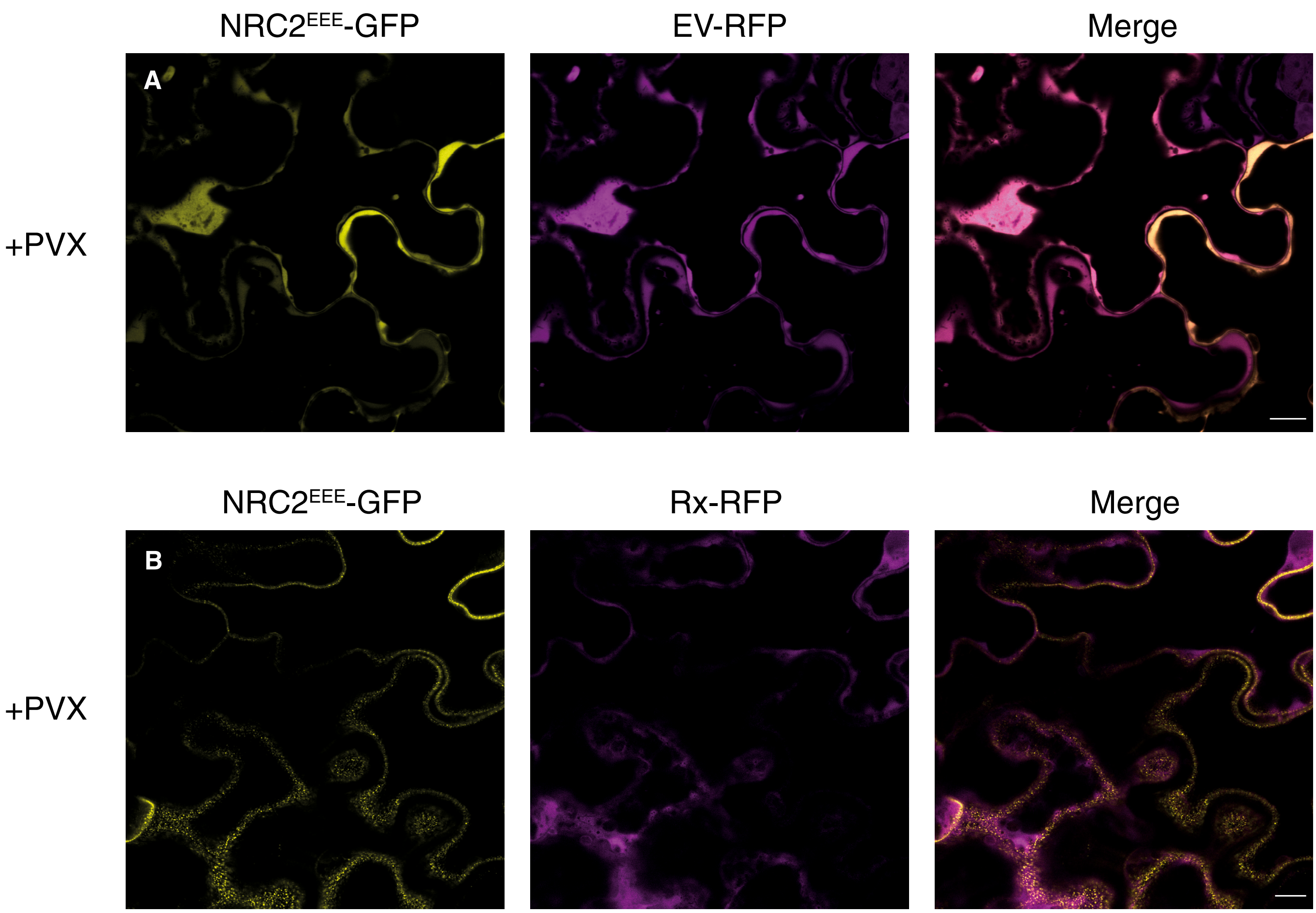

Figure 8. ***Potato virus X*** **infection triggers Rx-dependent NRC2 membrane-associated puncta.**

A, B Single-plane confocal micrographs show the localization of NRC2 together with Rx or free RFP during PVX infection. C-terminally GFP-tagged NRC2^{EEE} was co-infiltrated with either EV-RFP or Rx-RFP in leaves of *nrc2/3/4 N. benthamiana* CRISPR mutant lines and infected with PVX by agroinfection. Scale bars represent 10 μm. (A) In the absence of Rx, NRC2^{EEE}-GFP is localized to the cytoplasm during PVX infection. (B) When Rx is present, NRC2^{EEE} forms puncta associated with the PM during PVX infection.

research will dissect the precise role of this N-terminal motif in NRC and CC-NLR-mediated cell death.

The recent elucidation of multiple plant NLR structures has demonstrated that plant and metazoan NLRs exhibit functional differences despite several commonalities. In this work, we propose an activation-and-release working model for sensor-helper pairs in the NRC network (Fig 9). In this working model, effector-triggered activation of a sensor NLR leads to conformational changes, which relay a signal to downstream helpers, potentially via transient interactions. These helpers subsequently activate, oligomerize and form resistosomes. The activated helper complexes then part ways with their sensors and re-localize to the plasma membrane where they trigger cell death. It is possible that transient intermediates exist where sensors interact with their helpers to trigger their activation. However, BN-PAGE assays with differently sized versions of Rx and NRC2 and confocal microscopy suggest that a stable hetero-complex scenario is unlikely for the mature activated Rx-NRC2 system. This points to a biochemical model for plant paired NLR activation that differs from activation processes previously characterized for metazoan NLR pairs. We conclude that these plant and metazoan paired and networked NLRs exhibit distinct activation mechanisms and biochemical processes. Whether this activation-and-release model applies to other paired plant CC-NLRs or even other paired metazoan NLRs remains to be tested.

Figure 9. An activation-and-release working model for sensor-helper pairs in the NRC network.

Prior to effector-triggered activation, NRC-dependent sensors such as Rx and Bs2 are held in an inactive conformation by intramolecular interactions. Upon perceiving their cognate effectors, the sensors undergo a series of conformational changes that allow them to signal to NRC2 and mediate its homo-oligomerization and resistosome formation. The activated NRC2 resistosome separates from the sensors and accumulates at the PM. The sensors remain in the cytoplasm, separate from the activated helper.

▶

 © 2022 The Authors

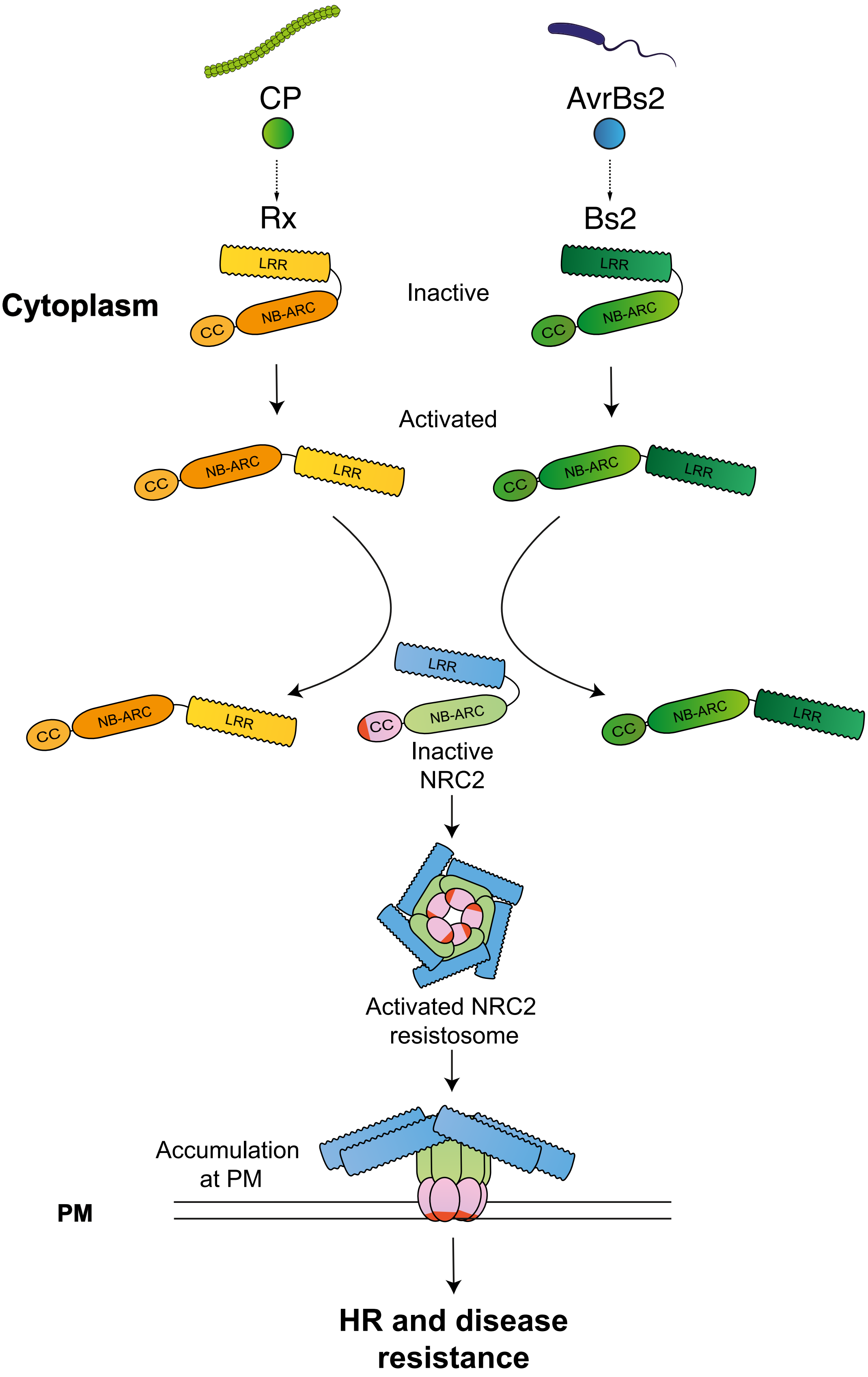

Figure 9.

© 2022 The Authors

Previous work has shown that following CP-triggered activation, Rx undergoes a series of conformational changes that lead to cell death and immune activation (Moffett *et al*, 2002; Rairdan & Moffett, 2006). Nonetheless, how a signal is relayed from sensor to helper remains unknown. While Rx does not oligomerize upon activation, the conformational switch may allow Rx to interact transiently with NRC2 in order to mediate its activation. To date, conclusive evidence that NRC-dependent sensors and their NRC helpers form stable complexes has not been obtained, possibly because the complexes are transient. Regardless of whether a direct or indirect interaction between sensor and helper mediates NRC activation, these findings and those of the companion study (preprint: Ahn *et al*, 2022) indicate that the mature NRC2 resistosome is released from the activated sensor. In this scenario, one Rx or Bs2 could potentially activate multiple NRC2 molecules, possibly triggering an NRC2 oligomerization cascade independent of the sensor. Alternatively, NRCs may form transient sensor-helper heterocomplexes with their sensor, which could act as an intermediate polymerization scaffold for the putative NRC resistosome, reminiscent of the first stages of NAIP/NLRC4 inflammasome maturation. A mechanism in which one sensor molecule can activate multiple NRC resistosomes would be much more efficient in amplifying immune signals as opposed to an activated sensor stably engaging in a sensor-helper heterocomplex. Such an amplification would be analogous to the working model for TIR-NLR/CC_R-NLR sensor-helper pairs, where small molecules produced by activated TIR-NLR sensors lead to downstream helper activation via the EDS1 signaling hub, triggering CC_R-NLR resistosome formation (preprint: Jacob *et al*, 2021; Sun *et al*, 2021; preprint: Huang *et al*, 2022). Further studies will shed more light on the dynamics of NRC resistosome assembly.

The PVX system allowed us to study paired NLR activation during pathogen infection, taking the state-of-the-art beyond activation with effector proteins (Figs 7 and 8). This work complements previous studies on NLR oligomerization upon heterologous expression of cognate effectors (Williams *et al*, 2014; Duxbury *et al*, 2020; Hu *et al*, 2020; Li *et al*, 2020; Ma *et al*, 2020; Martin *et al*, 2020), showing that the same mechanism likely applies during infection by pathogenic organisms. Investigating the oligomeric state and subcellular localization of paired/networked NLRs upon infection will provide further insights into the mechanisms that underpin NLR-mediated immunity. In the case of NRC4, this helper can focally accumulate at the interface between *P. infestans* and the host plant at the site where effectors are delivered before re-localizing and forming discrete puncta at the PM following activation (Duggan *et al*, 2021). Interestingly, the puncta observed for activated NRC2 and NRC4 are distributed throughout the cell PM. What is the exact nature of these puncta, how many resistosomes accumulate in the observed PM micro-domains and whether they form macro-complexes remain open questions.

A multitude of structural, biochemical and cell biology studies have contributed to our mechanistic understanding of NLR activation and signaling (Kourelis & Van Der Hoorn, 2018; Duxbury *et al*, 2021; Ngou *et al*, 2022). Our work expands on the current conceptual mechanistic framework of NLR activation by incorporating the higher order of genetic complexity presented by plant immune receptor networks (Wu *et al*, 2017, 2018). In the case of singleton NLRs, an individual protein can perceive effectors and subsequently form a resistosome to execute cell death. We previously proposed that in plant NLR networks, sensors have specialized over evolutionary time to perceive effectors and have lost the biochemical features required for executing cell death (Adachi *et al*, 2019a, 2019b). Based on our current working model (Fig 9), sensor NLRs have lost the capacity to oligomerize into resistosomes and have specialized in pathogen perception and helper activation. In contrast, activated NRC2 and NRC4 behave similarly to other MADA-CC-NLRs by forming resistosome-like homo-oligomers that translocate to the PM. Nonetheless, the many-to-many sensor-helper connections in the NRC network raise further questions. What is the precise nature of the activation signal relayed from sensor to helper? What are the precise dynamics of NRC resistosome assembly? How do the molecular determinants for sensor-helper specificity translate into resistosome formation? Addressing these questions holds the potential to advance our understanding of the diversity of plant NLR immune activation beyond functional singleton NLRs.

Materials and Methods

Plant growth conditions

Wild-type and *nrc2/3/4* CRISPR mutant *N. benthamiana* lines were grown in a controlled environment growth chamber with a temperature range of 22–25°C, humidity of 45–65% and a 16/8-h light/dark cycle.

Plasmid constructions

The Golden Gate Modular Cloning (MoClo) kit (Weber *et al*, 2011) and the MoClo plant parts kit (Engler *et al*, 2014) were used for cloning, and all vectors are from this kit unless specified otherwise. Effectors, receptors and NRCs were cloned into the binary vector pJK268c, which contains the Tomato bushy stunt virus silencing inhibitor p19 in the backbone (Kourelis *et al*, 2020). Cloning design and sequence analysis were done using Geneious Prime (v2021.2.2; https://www.geneious.com). Plasmid construction is described in Appendix Table S1.

Cell death assays by agroinfiltration

Effectors and NLR immune receptors of interest were transiently expressed according to previously described methods (Bos *et al*, 2006). Briefly, leaves from 4–5-week-old plants were infiltrated with suspensions of *A. tumefaciens* GV3101 pM90 strains transformed with expression vectors coding for different proteins indicated. Final OD_{600} of all *A. tumefaciens* suspensions were adjusted in infiltration buffer (10 mM MES, 10 mM $MgCl_2$, and 150 μM acetosyringone (pH 5.6)). Final OD_{600} used was 0.3 for each NLR immune receptor and 0.2 for all effectors, adding up to a total OD_{600} of 0.8.

Extraction of total proteins for BN-PAGE and SDS–PAGE assays

Four to five-week-old plants were agroinfiltrated as described above with constructs of interest and leaf tissue was collected 3 days post agroinfiltration or 2 days post agroinfiltration in experiments with NRC4 (Fig 5). Final OD_{600} used was 0.3 for each NLR immune

 © 2022 The Authors

receptor used and 0.2 for all effectors for a total OD_{600} of 0.8. BN-PAGE was performed using the Bis-Tris Native PAGE system (Invitrogen) according to the manufacturer's instructions. Leaf tissue was ground using a Geno/Grinder tissue homogenizer and total protein was subsequently extracted and homogenized extraction buffer. For NRC2, GTMN extraction buffer was used (10% glycerol, 50 mM Tris–HCl (pH 7.5), 5 mM $MgCl_2$ and 50 mM NaCl) supplemented with 10 mM DTT, 1x protease inhibitor cocktail (SIGMA) and 0.2% Nonidet P-40 Substitute (SIGMA). For NRC4, GHMN buffer (10% glycerol, 50 mM HEPES (pH 7.4), 5 mM $MgCl_2$ and 50 mM NaCl) buffer supplemented with 10 mM DTT, 1x protease inhibitor cocktail (SIGMA) and 1% Digitonin (SIGMA) was used for extraction. Samples were incubated in extraction buffer on ice for 10 min with short vortex mixing every 2 min. Following incubation, samples were centrifuged at 5,000 *g* for 15 min and the supernatant was used for BN-PAGE and SDS–PAGE assays.

BN-PAGE assays

For BN-PAGE, samples extracted as detailed above were diluted as per the manufacturer's instructions by adding NativePAGE 5% G-250 sample additive, 4x Sample Buffer and water. After dilution, samples were loaded and run on Native PAGE 3–12% Bis-Tris gels alongside either NativeMark unstained protein standard (Invitrogen) or SERVA Native Marker (SERVA). The proteins were then transferred to polyvinylidene difluoride membranes using NuPAGE Transfer Buffer using a Trans-Blot Turbo Transfer System (Bio-Rad) as per the manufacturer's instructions. Proteins were fixed to the membranes by incubating with 8% acetic acid for 15 min, washed with water and left to dry. Membranes were subsequently re-activated with methanol in order to correctly visualize the unstained native protein marker. Membranes were immunoblotted as described below.

SDS–PAGE assays

For SDS–PAGE, samples were diluted in SDS loading dye and denatured at 72 °C for 10 min. Denatured samples were spun down at 5,000 *g* for 3 min and supernatant was run on 4–20% Bio-Rad 4–20% Mini-PROTEAN TGX gels alongside a PageRuler Plus prestained protein ladder (Thermo Scientific). The proteins were then transferred to polyvinylidene difluoride membranes using Trans-Blot Turbo Transfer Buffer using a Trans-Blot Turbo Transfer System (Bio-Rad) as per the manufacturer's instructions. Membranes were immunoblotted as described below.

Immunoblotting and detection of BN-PAGE and SDS–PAGE assays

Blotted membranes were blocked with 5% milk in Tris-buffered saline plus 0.01% Tween 20 (TBS-T) for an hour at room temperature and subsequently incubated with desired antibodies at 4°C overnight. Antibodies used were anti-GFP (B-2) HRP (Santa Cruz Biotechnology), anti-HA (3F10) HRP (Roche), anti-Myc (9E10) HRP (Roche), and anti-FLAG (M2) HRP (Sigma), all used in a 1:5,000 dilution in 5% milk in TBS-T. To visualize proteins, we used Pierce ECL Western (32106, Thermo Fisher Scientific), supplementing with up to 50% SuperSignal West Femto Maximum Sensitivity Substrate (34095, Thermo Fishes Scientific) when necessary. Membrane imaging was carried out with an ImageQuant LAS 4000 or an ImageQuant 800 luminescent imager (GE Healthcare Life Sciences, Piscataway, NJ). Rubisco loading control was stained using Ponceau S (Sigma) or Ponceau 4R (AG Barr).

Gel filtration assays

Protein was extracted as described above. Protein extracts were filtered through a 0.45 μm filter (Sartorius) and 200 μl of filtered extract were analyzed by gel filtration. Extracts were run on a Superdex 200 Increase 10/300 GL column (GE Healthcare) connected to an AKTA Pure system (GE Healthcare), with samples being run at a flow rate of 0.4 ml/min. The buffer used for elution was GTMN extraction buffer (10% glycerol, 50 mM Tris–HCl (pH 7.5), 5 mM $MgCl_2$ and 50 mM NaCl) supplemented with 1 mM DTT, to match the extraction buffer used for NRC2. The eluted fractions were analyzed by SDS–PAGE as described above and immunoblotted with the corresponding antibodies.

BN and SDS–PAGE assays with PVX infection (agroinfection)

Four to five-week-old plants were agroinfiltrated as described above with constructs of interest. Simultaneously, PVX was delivered by agroinfection using an *A. tumefaciens* strain carrying GFP-labeled PVX (pGR106-PVX-GFP). Final OD_{600} used was 0.3 for each NLR immune receptor used and 0.05 for the *A. tumefaciens* strain carrying PVX or free GFP for a total OD_{600} of 0.65. Leaf tissue was collected 3 days post agroinfiltration. BN-PAGE and SDS–PAGE assays were carried out as described above.

Confocal microscopy

Three to four-week-old plants were agroinfiltrated as described above with constructs of interest. PVX was delivered as before, or the coat protein CP-4xMyc or EV control at OD_{600} 0.1; Rx-RFP at OD_{600} of 0.25 and NRC2^{EEE}-GFP at OD_{600} of 0.25. Leaf tissue was prepared for imaging by sectioning of desired area surrounding an infection spot using a cork borer size 4, and were mounted, live, in wells containing dH2O made in Carolina Observation Gel to enable diffusion of gasses. The abaxial of the leaf tissue was imaged using a Leica SP8 with 40x water immersion objective. Laser excitations for fluorescent proteins were used as described previously (Duggan *et al*, 2021), namely 488 nm (Argon) for GFP, 561/594 nm (Diode) for RFP and 405 nm (Diode) for BFP.

Membrane enrichment assays

Membrane enrichment was carried out by slightly modifying a previously described protocol (Abas & Luschnig, 2010). In brief, leaf material was ground to fine powder using liquid nitrogen and 2x volume of extraction buffer was added. Extraction buffer consisted of 0.81 M sucrose, 5% (v/v) glycerol, 10 mM EDTA, 10 mM EGTA, 5 mM KCl, and 100 mM Tris–HCl (pH 7.5) supplemented with 5 mM DTT, 1% Sigma Plant Protease Inhibitor Cocktail, 1 mM PMSF and 0.5% PVPP. After addition of the buffer, the samples were vortexed for a minute and the cell debris was cleared out by two subsequent centrifugation steps at 1,000 *g* for 5 min. The supernatant was diluted 1:1 using distilled water and an aliquot of the

supernatant was separated as the total fraction (T). The remaining supernatant (200–300 μl) was further centrifuged at 21,000 *g* for 90 min at 4°C. This centrifugation yielded the supernatant (soluble fraction, S) and membrane enriched pellet (membrane fraction, M). After separating the soluble fraction, the pellet was resuspended in diluted extraction buffer (without PVPP). All the fractions were diluted with SDS loading dye, and proteins were denatured by incubating at 50°C for 15 min. Western blotting was performed as previously described following SDS–PAGE. Endogenous plasma membrane ATPase was detected using anti-H + ATPase (AS07 260) antibody (Agrisera) as a marker to show the success of membrane enrichment.

Data availability

All relevant data are within the article, in the Appendix and in the Source Data files. This study includes no data that would need to be deposited in external repositories.

Expanded View for this article is available online.

Acknowledgements
We are very thankful to several colleagues for discussions and ideas. We thank Daniel Lüdke (The Sainsbury Laboratory, Norwich, UK) for valuable comments on this article. We thank all members of the TSL Support Services for their invaluable assistance. We thank S. Hrek. M.C. and S.K. dedicate this article to the memory of Diego A. Maradona. A.V.C. was funded by the John Innes Foundation and TSL. The Kamoun Lab is funded primarily from the Gatsby Charitable Foundation, Biotechnology and Biological Sciences Research Council (BBSRC, UK, BB/WW002221/1, BB/V002937/1, BBS/E/J/000PR9795 and BBS/E/J/000PR9796) and the European Research Council (BLASTOFF).

Author contributions
Mauricio P Contreras: Conceptualization; data curation; formal analysis; supervision; validation; investigation; visualization; methodology; writing – original draft; project administration; writing—review and editing. **Hsuan Pai:** Data curation; formal analysis; validation; investigation; methodology. **Yasin Tumtas:** Data curation; formal analysis; validation; investigation; visualization; methodology; writing—review and editing. **Cian Duggan:** Data curation; formal analysis; validation; investigation; visualization; methodology; writing—review and editing. **Enoch Lok Him Yuen:** Data curation; validation; investigation; visualization; writing—review and editing. **Angel Vergara Cruces:** Data curation; formal analysis; validation; investigation; visualization; writing—review and editing. **Jiorgos Kourelis:** Resources; writing—review and editing. **Hee-Kyung Ahn:** Conceptualization; methodology; writing—review and editing. **Kim-Teng Lee:** Investigation. **Chih-Hang Wu:** Conceptualization; methodology; writing—review and editing. **Tolga O Bozkurt:** Resources; supervision; funding acquisition; project administration. **Lida Derevnina:** Conceptualization; resources; supervision; project administration; writing—review and editing. **Sophien Kamoun:** Conceptualization; resources; supervision; funding acquisition; visualization; writing—original draft; project administration; writing—review and editing.

Disclosure and competing interest statement
C.D., T.O.B and S.K. receive funding from industry on NLR biology. S.K. has filed patents on NLR biology.

References

Abas L, Luschnig C (2010) Maximum yields of microsomal-type membranes from small amounts of plant material without requiring ultracentrifugation. *Anal Biochem* 401: 217 – 227

Adachi H, Contreras MP, Harant A, Wu C-h, Derevnina L, Sakai T, Duggan C, Moratto E, Bozkurt TO, Maqbool A *et al* (2019a) An N-terminal motif in NLR immune receptors is functionally conserved across distantly related plant species. *Elife* 8: e49956

Adachi H, Derevnina L, Kamoun S (2019b) NLR singletons, pairs, and networks: evolution, assembly, and regulation of the intracellular immunoreceptor circuitry of plants. *Curr Opin Plant Biol* 50: 121 – 131

Adachi H, Sakai T, Kourelis J, Pai H, Gonzalez Hernandez JL, Maqbool A, Kamoun S (2022) Jurassic NLR: conserved and dynamic evolutionary features of the atypically ancient immune receptor ZAR1. *bioRxiv* https://doi.org/10.1101/2020.10.12.333484 [PREPRINT]

Ahn H-K, Lin X, Olave-Achury AC, Derevnina L, Contreras MP, Kourelis J, Kamoun S, Jones JDG (2022) Effector-dependent activation and oligomerization of NRC helper NLRs by Rpi-amr3 and Rpi-amr1. *bioRxiv* https://doi.org/10.1101/2022.04.25.489359 [PREPRINT]

Bendahmane A, Köhm BA, Dedi C, Baulcombe DC (1995) The coat protein of potato virus X is a strain-specific elicitor of Rx1-mediated virus resistance in potato. *Plant J* 8: 933 – 941

Bendahmane A, Kanyuka K, Baulcombe DC (1999) The Rx gene from potato controls separate virus resistance and cell death responses. *Plant Cell* 11: 781 – 791

Bi G, Su M, Li N, Liang Y, Dang S, Xu J, Hu M, Wang J, Zou M, Deng Y (2021) The ZAR1 resistosome is a calcium-permeable channel triggering plant immune signaling. *Cell* 184: 3528 – 3541.e12

Bos JI, Kanneganti TD, Young C, Cakir C, Huitema E, Win J, Armstrong MR, Birch PR, Kamoun S (2006) The C-terminal half of Phytophthora infestans RXLR effector AVR3a is sufficient to trigger R3a-mediated hypersensitivity and suppress INF1-induced cell death in Nicotiana benthamiana. *Plant J* 48: 165 – 176

Césari S, Kanzaki H, Fujiwara T, Bernoux M, Chalvon V, Kawano Y, Shimamoto K, Dodds P, Terauchi R, Kroj T (2014) The NB-LRR proteins RGA 4 and RGA 5 interact functionally and physically to confer disease resistance. *EMBO J* 33: 1941 – 1959

De la Concepcion JC, Benjumea JV, Bialas A, Terauchi R, Kamoun S, Banfield MJ (2021) Functional diversification gave rise to allelic specialization in a rice NLR immune receptor pair. *eLife* 10: e71662

Derevnina L, Contreras MP, Adachi H, Upson J, Vergara Cruces A, Xie R, Skłenar J, Menke FLH, Mugford ST, MacLean D *et al* (2021) Plant pathogens convergently evolved to counteract redundant nodes of an NLR immune receptor network. *PLoS Biol* 19: e3001136

Duggan C, Moratto E, Savage Z, Hamilton E, Adachi H, Wu C-H, Leary AY, Tumtas Y, Rothery SM, Maqbool A *et al* (2021) Dynamic localization of a helper NLR at the plant–pathogen interface underpins pathogen recognition. *Proc Natl Acad Sci* 118: 118

Duxbury Z, Ma Y, Furzer OJ, Huh SU, Cevik V, Jones JD, Sarris PF (2016) Pathogen perception by NLRs in plants and animals: parallel worlds. *Bioessays* 38: 769 – 781

Duxbury Z, Wang S, MacKenzie CI, Tenthorey JL, Zhang X, Huh SU, Hu L, Hill L, Ngou PM, Ding P *et al* (2020) Induced proximity of a TIR signaling domain on a plant-mammalian NLR chimera activates defense in plants. *Proc Natl Acad Sci* 117: 18832 – 18839

© 2022 The Authors

Duxbury Z, Wu C-h, Ding P (2021) A comparative overview of the intracellular guardians of plants and animals: NLRs in innate immunity and beyond. *Annu Rev Plant Biol* 72: 155 – 184

Engler C, Youles M, Gruetzner R, Ehnert T-M, Werner S, Jones JD, Patron NJ, Marillonnet S (2014) A golden gate modular cloning toolbox for plants. *ACS Synth Biol* 3: 839 – 843

Feehan JM, Castel B, Bentham AR, Jones JDG (2020) Plant NLRs get by with a little help from their friends. *Curr Opin Plant Biol* 56: 99 – 108

Förderer A, Li E, Lawson A, Deng Y-n, Sun Y, Logemann E, Zhang X, Wen J, Han Z, Chang J *et al* (2022) A wheat resistosome defines common principles of immune receptor channels. *bioRxiv* https://doi.org/10.1101/2022.03.23.485489 [PREPRINT]

Gantner J, Ordon J, Kretschmer C, Guerois R, Stuttmann J (2019) An EDS1-SAG101 complex functions in TNL-mediated immunity in Solanaceae. *bioRxiv* https://doi.org/10.1101/511956 [PREPRINT]

Grinzato A, Kandiah E, Lico C, Betti C, Baschieri S, Zanotti G (2020) Atomic structure of potato virus X, the prototype of the Alphaflexiviridae family. *Nat Chem Biol* 16: 564 – 569

Hu Z, Zhou Q, Zhang C, Fan S, Cheng W, Zhao Y, Shao F, Wang H-W, Sui S-F, Chai J (2015) Structural and biochemical basis for induced self-propagation of NLRC4. *Science* 350: 399 – 404

Hu M, Qi J, Bi G, Zhou J-M (2020) Bacterial effectors induce oligomerization of immune receptor ZAR1 in vivo. *Mol Plant* 13: 793 – 801

Huang S, Jia A, Song W, Hessler G, Meng Y, Sun Y, Xu L, Laessle H, Jirschitzka J, Ma S *et al* (2022) Identification and receptor mechanism of TIR-catalyzed small molecules in plant immunity. *bioRxiv* https://doi.org/10.1101/2022.04.01.486681 [PREPRINT]

Jacob P, Kim NH, Wu F, El-Kasmi F, Walton WG, Furzer OJ, Lietzan AD, Sunil S, Kempthorn K, Redinbo MR *et al* (2021) The plant immune receptors NRG1. 1 and ADR1 are calcium influx channels. *bioRxiv* https://doi.org/10.1101/2021.02.25.431980 [PREPRINT]

Jones JD, Dangl JL (2006) The plant immune system. *Nature* 444: 323 – 329

Jones JD, Vance RE, Dangl JL (2016) Intracellular innate immune surveillance devices in plants and animals. *Science* 354: aaf6395

Kim YK, Shin J-S, Nahm MH (2016) NOD-like receptors in infection, immunity, and diseases. *Yonsei Med J* 57: 5 – 14

Kofoed EM, Vance RE (2011) Innate immune recognition of bacterial ligands by NAIPs determines inflammasome specificity. *Nature* 477: 592 – 595

Kourelis J, Van Der Hoorn RA (2018) Defended to the nines: 25 years of resistance gene cloning identifies nine mechanisms for R protein function. *Plant Cell* 30: 285 – 299

Kourelis J, Malik S, Mattinson O, Krauter S, Kahlon PS, Paulus JK, van der Hoorn RA (2020) Evolution of a guarded decoy protease and its receptor in solanaceous plants. *Nat Commun* 11: 1 – 15

Kourelis J, Contreras MP, Harant A, Adachi H, Derevnina L, Wu C-H, Kamoun S (2021a) The helper NLR immune protein NRC3 mediates the hypersensitive cell death caused by the cell-surface receptor Cf-4. *bioRxiv* https://doi.org/10.1101/2021.09.28.461843 [PREPRINT]

Kourelis J, Sakai T, Adachi H, Kamoun S (2021b) RefPlantNLR is a comprehensive collection of experimentally validated plant disease resistance proteins from the NLR family. *PLoS Biol* 19: e3001124

Lapin D, Kovacova V, Sun X, Dongus JA, Bhandari D, von Born P, Bautor J, Guarneri N, Rzemieniewski J, Stuttmann J (2019) A coevolved EDS1-SAG101-NRG1 module mediates cell death signaling by TIR-domain immune receptors. *Plant Cell* 31: 2430 – 2455

Lechtenberg BC, Mace PD, Riedl SJ (2014) Structural mechanisms in NLR inflammasome signaling. *Curr Opin Struct Biol* 29: 17 – 25

Lee HY, Mang H, Choi E, Seo YE, Kim MS, Oh S, Kim SB, Choi D (2021) Genome-wide functional analysis of hot pepper immune receptors reveals an autonomous NLR clade in seed plants. *New Phytol* 229: 532 – 547

Li L, Habring A, Wang K, Weigel D (2020) Atypical resistance protein RPW8/HR triggers Oligomerization of the NLR immune receptor RPP7 and autoimmunity. *Cell Host Microbe* 27: 405 – 417.e6

Ma S, Lapin D, Liu L, Sun Y, Song W, Zhang X, Logemann E, Yu D, Wang J, Jirschitzka J *et al* (2020) Direct pathogen-induced assembly of an NLR immune receptor complex to form a holoenzyme. *Science* 370: eabe3069

Maekawa T, Kufer TA, Schulze-Lefert P (2011) NLR functions in plant and animal immune systems: so far and yet so close. *Nat Immunol* 12: 817 – 826

Martin R, Qi T, Zhang H, Liu F, King M, Toth C, Nogales E, Staskawicz BJ (2020) Structure of the activated ROQ1 resistosome directly recognizing the pathogen effector XopQ. *Science* 370: eabd9993

Moffett P, Farnham G, Peart J, Baulcombe DC (2002) Interaction between domains of a plant NBS–LRR protein in disease resistance-related cell death. *EMBO J* 21: 4511 – 4519

Ngou BPM, Ding P, Jones JD (2021) Channeling plant immunity. *Cell* 184: 3358 – 3360

Ngou BPM, Ding P, Jones JDG (2022) Thirty years of resistance: zig-zag through the plant immune system. *Plant Cell* 34: 1447 – 1478

Qu Y, Misaghi S, Izrael-Tomasevic A, Newton K, Gilmour LL, Lamkanfi M, Louie S, Kayagaki N, Liu J, Kömüves L *et al* (2012) Phosphorylation of NLRC4 is critical for inflammasome activation. *Nature* 490: 539 – 542

Rairdan GJ, Moffett P (2006) Distinct domains in the ARC region of the potato resistance protein Rx mediate LRR binding and inhibition of activation. *Plant Cell* 18: 2082 – 2093

Saile SC, Jacob P, Castel B, Jubic LM, Salas-Gonzáles I, Bäcker M, Jones JD, Dangl JL, El Kasmi F (2020) Two unequally redundant “helper” immune receptor families mediate *Arabidopsis thaliana* intracellular “sensor” immune receptor functions. *PLoS Biol* 18: e3000783

Sun X, Lapin D, Feehan JM, Stolze SC, Kramer K, Dongus JA, Rzemieniewski J, Blanvillain-Baufumé S, Harzen A, Bautor J *et al* (2021) Pathogen effector recognition-dependent association of NRG1 with EDS1 and SAG101 in TNL receptor immunity. *Nat Commun* 12: 1 – 15

Tameling WI, Baulcombe DC (2007) Physical association of the NB-LRR resistance protein Rx with a ran GTPase–activating protein is required for extreme resistance to potato virus X. *Plant Cell* 19: 1682 – 1694

Vance RE (2015) The NAIP/NLRC4 inflammasomes. *Curr Opin Immunol* 32: 84 – 89

Wang J, Hu M, Wang J, Qi J, Han Z, Wang G, Qi Y, Wang H-W, Zhou J-M, Chai J (2019a) Reconstitution and structure of a plant NLR resistosome conferring immunity. *Science* 364: eaav5870

Wang J, Wang J, Hu M, Wu S, Qi J, Wang G, Han Z, Qi Y, Gao N, Wang H-W *et al* (2019b) Ligand-triggered allosteric ADP release primes a plant NLR complex. *Science* 364: eaav5868

Weber E, Engler C, Gruetzner R, Werner S, Marillonnet S (2011) A modular cloning system for standardized assembly of multigene constructs. *PloS One* 6: e16765

Williams SJ, Sohn KH, Wan L, Bernoux M, Sarris PF, Segonzac C, Ve T, Ma Y, Saucet SB, Ericsson DJ *et al* (2014) Structural basis for assembly and function of a heterodimeric plant immune receptor. *Science* 344: 299 – 303

Wu C-H, Abd-El-Haliem A, Bozkurt TO, Belhaj K, Terauchi R, Vossen JH, Kamoun S (2017) NLR network mediates immunity to diverse plant pathogens. *Proc Natl Acad Sci USA* 114: 8113 – 8118

© 2022 The Authors

Wu C-H, Derevnina L, Kamoun S (2018) Receptor networks underpin plant immunity. *Science* 360: 1300 – 1301

Zdrzałek R, Kamoun S, Terauchi R, Saitoh H, Banfield MJ (2020) The rice NLR pair Pikp-1/Pikp-2 initiates cell death through receptor cooperation rather than negative regulation. *PloS One* 15: e0238616

Zhang L, Chen S, Ruan J, Wu J, Tong AB, Yin Q, Li Y, David L, Lu A, Wang WL *et al* (2015) Cryo-EM structure of the activated NAIP2-NLRC4 inflammasome reveals nucleated polymerization. *Science* 350: 404 – 409

Zhao Y, Yang J, Shi J, Gong Y-N, Lu Q, Xu H, Liu L, Shao F (2011) The NLRC4 inflammasome receptors for bacterial flagellin and type III secretion apparatus. *Nature* 477: 596 – 600

License: This is an open access article under the terms of the Creative Commons Attribution License, which permits use, distribution and reproduction in any medium, provided the original work is properly cited.

 © 2022 The Authors

Expanded View Figures

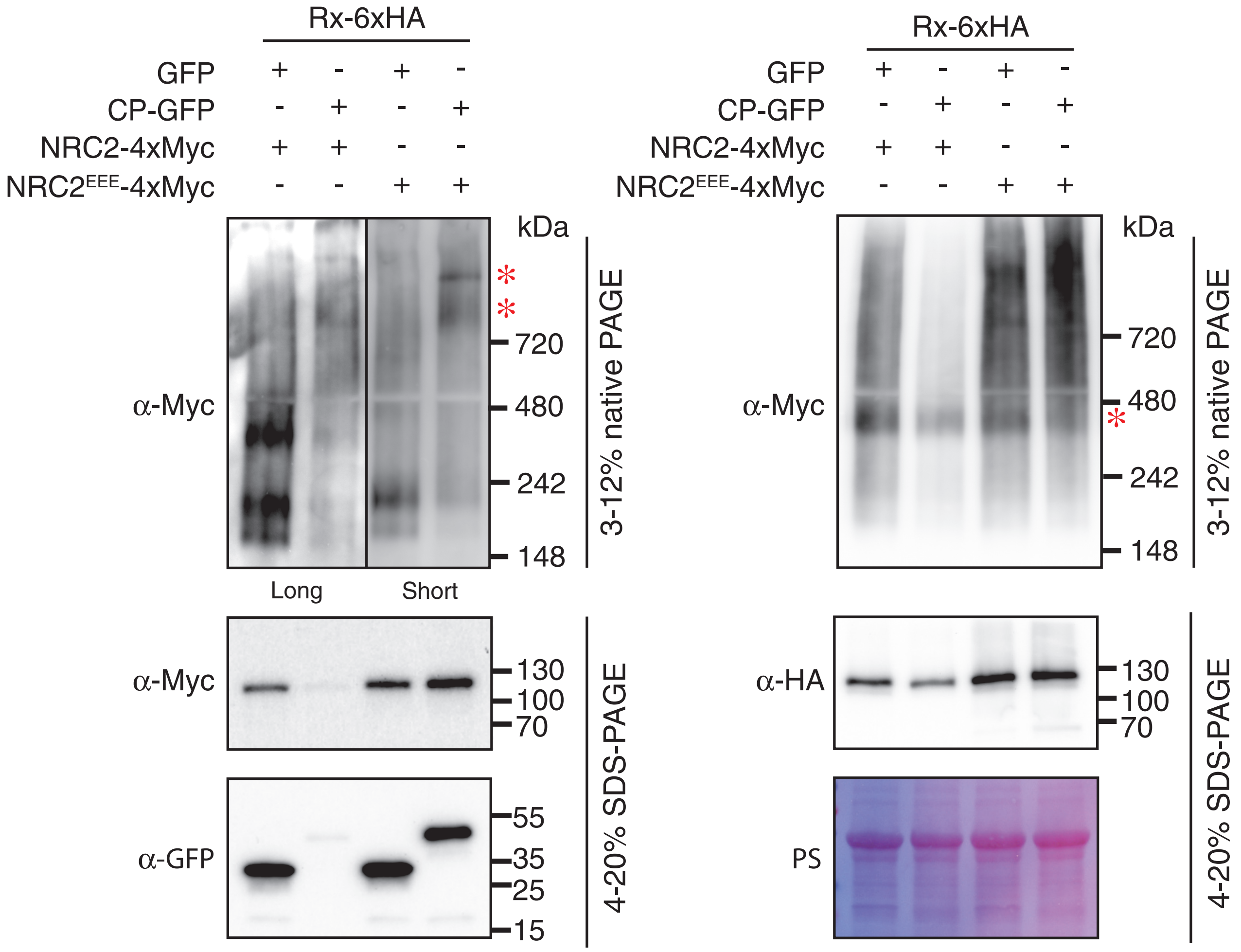

Figure EV1. NRC2 with an intact N-terminal MADA motif also oligomerizes upon Rx-mediated activation.

BN-PAGE and SDS–PAGE assays with inactive and activated Rx-NRC2. C-terminally 6xHA tagged Rx and C-terminally 4xMyc-tagged NRC2 or NRC2^{EEE} were co-expressed with either free GFP or C-terminally GFP-tagged CP. Total protein was extracted with a Tris–HCl-based buffer, as described in materials and methods. Extracts were run on native and denaturing PAGE assays in parallel and immunoblotted with the appropriate antisera labeled on the left. Approximate molecular weights (kDa) of the proteins are shown on the right. Red asterisks indicate bands corresponding to the activated NRC2 complex (α-Myc blot) and Rx (α-HA blot). Given that the ongoing cell death triggered by NRC2 activation resulted in lower protein accumulation, we showed different exposures (long and short) as indicated by the black line. Rubisco loading control was carried out using Ponceau stain (PS). The experiment was repeated 2 times.

Source data are available online for this figure.

© 2022 The Authors

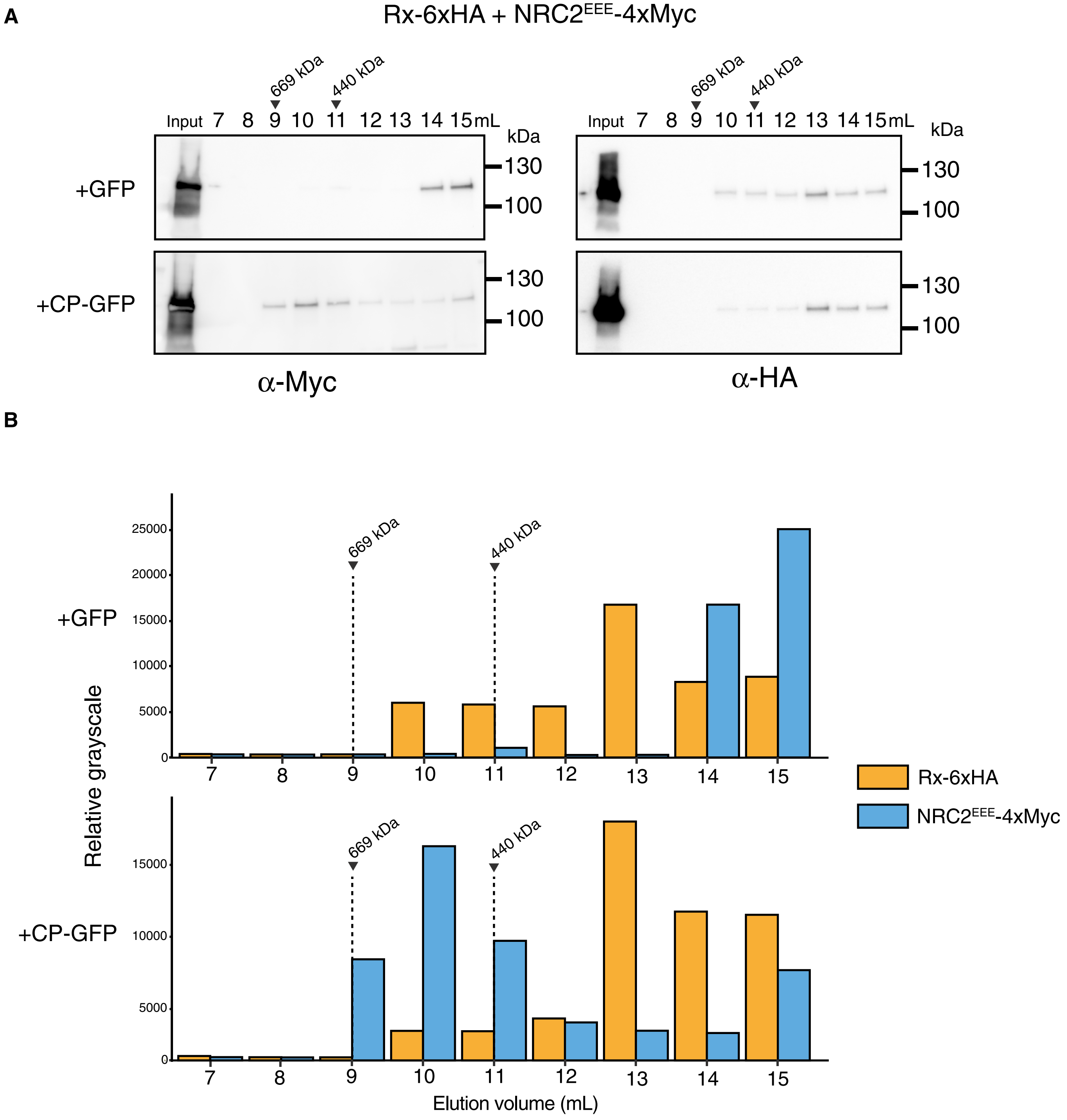

Figure EV2. Rx-mediated NRC2 oligomerization can be visualized using gel filtration assays.

A Gel filtration assays with inactive and activated Rx-NRC2^{EEE}. C-terminally 6xHA tagged Rx and C-terminally 4xMyc-tagged NRC2^{EEE} were co-expressed with either free GFP or C-terminally GFP-tagged CP. Total protein extracts were run on an S200 10/300 analytical column. A range of fractions were ran on SDS–PAGE and immunoblotted with the appropriate antisera labeled below. Approximate molecular weights (kDa) of the proteins are shown on the right.

B Relative gray scales indicate the arbitrary densitometry units of different proteins as visualized by immunoblotting. Elution volume of standard molecular masses (669 kDa and 440 kDa) is shown above. The experiment was repeated 2 times and a representative image is shown.

Source data are available online for this figure.

 © 2022 The Authors

Sensor NLR immune proteins activate oligomerization of their NRC helpers

Mauricio P. Contreras[1], Hsuan Pai[1], Yasin Tumtas[2], Cian Duggan[2], Enoch Lok Him Yuen[2], Angel Vergara Cruces[1,‡], Jiorgos Kourelis[1], Hee-Kyung Ahn[1], Kim-Teng Lee[3], Chih-Hang Wu[3], Tolga O. Bozkurt[2], Lida Derevnina[1,†,*] and Sophien Kamoun[1*]

1: The Sainsbury Laboratory, University of East Anglia, Norwich, United Kingdom.
2: Department of Life Sciences, Imperial College, London, United Kingdom.
3: Institute of Plant and Microbial Biology, Academia Sinica, Taipei, Taiwan.

‡Current address: John Innes Centre, University of East Anglia, Norwich, United Kingdom.
†Current address: Crop Science Centre, Department of Plant Sciences, University of Cambridge, Cambridge, United Kingdom.

*Correspondence to: ld645@cam.ac.uk (LD); sophien.kamoun@tsl.ac.uk (SK)

Appendix

Table of contents

Appendix Figure S1: MADA motif mutants of NRC2 are unable to trigger cell death……………………………………………………………………………2

Appendix Figure S2: C-terminally tagged sensor and helper NLRs retain the capacity to trigger hypersensitive cell death……………………………………....3

Appendix Figure S3: C-terminally 6xHA tagged sensor NLRs retain the capacity to mediate cell death…………………………………………………………..4

Appendix Figure S4: Fluorescent protein-tagged Rx and NRC2 retain cell death-mediating capacity and can oligomerize upon activation……………………….....5

Appendix Table S1: List of primers and constructs used in this study…………6

Appendix Figure S1:

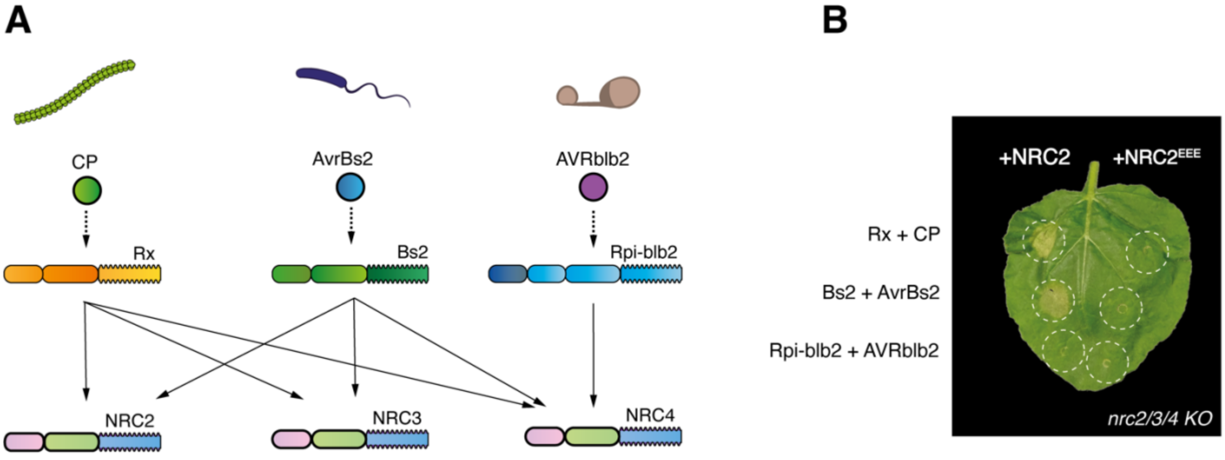

Appendix Figure S1: MADA motif mutants of NRC2 are unable to trigger cell death
(**A**) The Solanaceous NRC immune receptor network. Sensor NLRs such as Rx, Bs2 and Rpi-blb2 have specialized in detecting effectors from pathogens as diverse as viruses, bacteria and oomycetes. Sensors in the NRC network signal, sometimes redundantly, through their downstream helper NLRs, the NRCs. Different sensors exhibit different helper specificities. For example, Rx and Bs2 which recognize CP and AvrBs2 respectively, can signal through NRC2, NRC3, and NRC4. Rpi-blb2, which recognizes AVRblb2, can only signal through NRC4. Some sensor NLRs exhibit N-terminal extensions, represented in dark blue. (**B**) Unlike NRC2, $NRC2^{EEE}$ does not complement Rx/CP and Bs2/AvrBs2-triggered hypersensitive cell death in leaves of *nrc2/3/4 N. benthamiana* CRISPR mutant lines. Representative leaves infiltrated with the appropriate constructs were photographed 5-7 days after infiltration. NRC2 and $NRC2^{EEE}$ constructs are C-terminally 4xMyc-tagged. All effectors used are C-terminally GFP-tagged. All sensors used are C-terminally 6xHA tagged. One representative leaf is shown.

Appendix Figure S2:

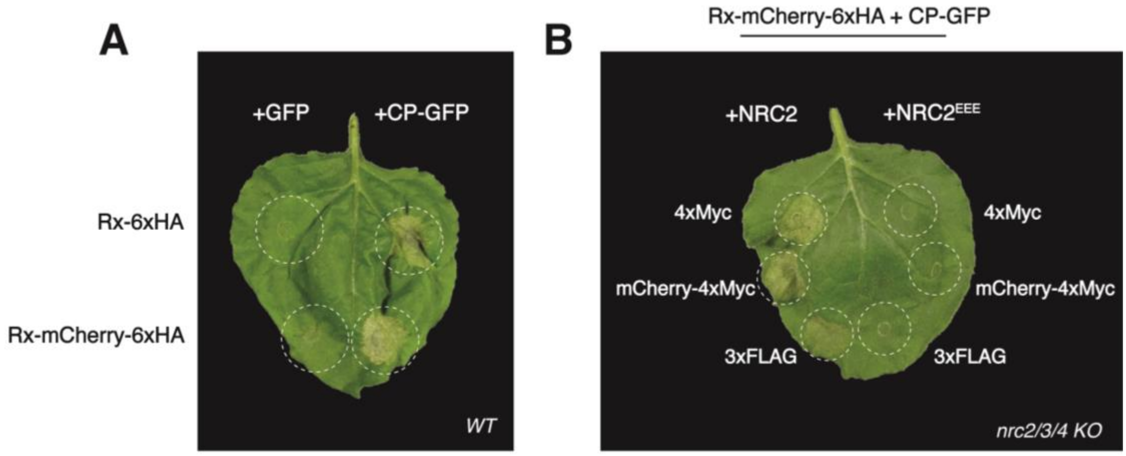

Appendix Figure S2: C-terminally tagged sensor and helper NLRs retain the capacity to trigger hypersensitive cell death.
(**A**) Much like C-terminally 6xHA tagged Rx, C-terminally mCherry-6xHA tagged Rx can mediate hypersensitive cell death when activated by CP. Representative leaves of *WT N. benthamiana* were infiltrated with the appropriate constructs and photographed 5-7 days after infiltration. C-terminal tags are indicated. Free GFP (+GFP) was used as a negative control for C-terminally GFP-tagged CP (+CP-GFP). One representative leaf is shown. (**B**) Rx-mCherry-6xHA is compatible with all C-terminally tagged versions of NRC2 tested. Rx/CP-triggered hypersensitive cell death was complemented by C-terminally 4xMyc, mCherry-4xMyc and 3xFLAG variants of NRC2 respectively in leaves of *nrc2/3/4 N. benthamiana* CRISPR mutant lines when Rx was C-terminally tagged with mCherry-6xHA. The corresponding $NRC2^{EEE}$ variants with the same C-terminal tag were no longer able to complement hypersensitive cell death. Representative leaves were infiltrated with the appropriate constructs and photographed 5-7 days after infiltration. One representative leaf is shown.

Appendix Figure S3:

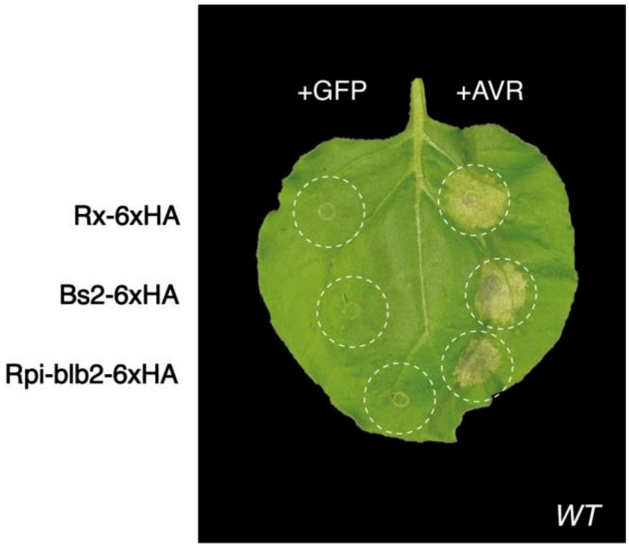

Appendix Figure S3: C-terminally 6xHA tagged sensor NLRs retain the capacity to mediate cell death.

C-terminally 6xHA tagged Rx, Bs2 and Rpi-blb2 can mediate hypersensitive cell death when activated by CP, AvrBs2 and AVRblb2, respectively. Representative leaves of *WT N. benthamiana* were infiltrated with the appropriate constructs and photographed 5-7 days after infiltration. Free GFP was used as a negative control for C-terminally GFP-tagged effectors (AVR). One representative leaf is shown.

Appendix Figure S4:

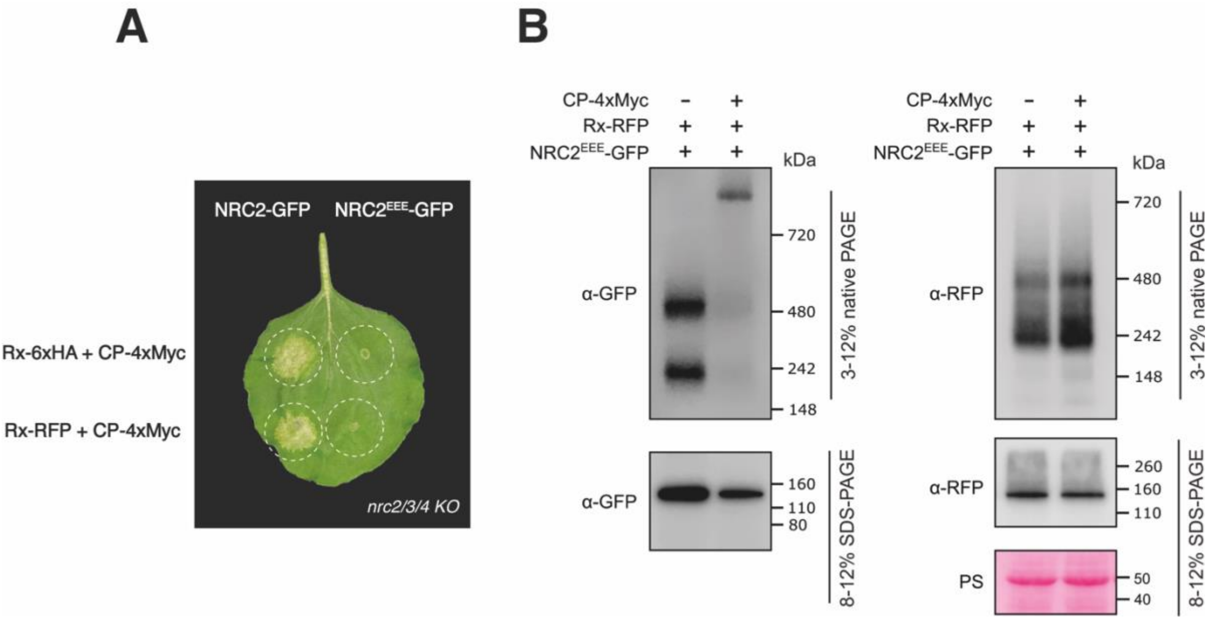

Appendix Figure S4: Fluorescent protein-tagged Rx and NRC2 retain cell death-mediating capacity and can oligomerize upon activation.

(**A**) C-terminally GFP-tagged NRC2 complements Rx/CP cell death in leaves of *nrc2/3/4 N. benthamiana* CRISPR mutant lines when Rx is C-terminally tagged with 6xHA or RFP. This cell death is not complemented with C-terminally GFP-tagged NRC2^{EEE}. Representative leaves were infiltrated with the appropriate constructs and photographed 5-7 days after infiltration. One representative leaf is shown. (**B**) BN-PAGE and SDS-PAGE assays performed in parallel on protein extracts used for membrane enrichment assays with inactive and activated C-terminally RFP-tagged Rx and C-terminally GFP-tagged NRC2^{EEE}. Total protein was extracted with a Tris-HCl-based buffer, as described in materials and methods. Extracts were run on native and denaturing PAGE assays in parallel and immunoblotted with the appropriate antisera labelled on the left. Approximate molecular weights (kDa) of the proteins are shown on the right. Rubisco loading control was carried out using Ponceau stain (PS). The experiment was repeated 2 times.

Appendix Table S1:

Appendix Table S1: List of primers and constructs used in this study.

Product	Antibiotic	Construct
pL2M-P19-2x35S::NbNRC2a-4xMyc	Kan	pJK-B2-090p-Myc
pL2M-P19-2x35S::Rx-6xHA	Kan	pJK-B2-178p-HA
pL2M-P19-2x35S::Bs2-6xHA	Kan	pJK-B2-219p-HA
pL2M-P19-2x35S::NbNRC2a^L9E/L13E/L17E-3xFLAG	Kan	pJK-B2-252p-FLAG
pL2M-P19-2x35S::NbNRC2a^L9E/L13E/L17E-4xMyc	Kan	pJK-B2-252p-Myc
pL2M-P19-2x35S::NbNRC2a-mEGFP	Kan	pMC-B2-203p-mEGFP
pL2M-P19-2x35S::NbNRC4^L9A/V10A/L14A-4xMyc	Kan	pMC-B2-179-4xMyc
pL2M-P19-2x35S::NbNRC2a^L9E/L13E/L17E-mEGFP	Kan	pMC-B2-204p-mEGFP
pL2M-P19-2x35S::Rpiblb2-6xHA	Kan	pMC-B2-164p-HA
pL2M-P19-2x35S::EGFP	Kan	pJK-B2-022p
pL2M-P19-2x35S::NbNRC2a-mCherry-4xMYC	Kan	pJK-B2-618p
pL2M-P19-2x35S::NbNRC2a^L9E/L13E/L17E-mCherry-4xMYC	Kan	pJK-B2-619p
pL2M-P19-2x35S::Rx-mCherry-6xHA	Kan	pJK-B2-620p
pL2M-P19-2x35S::AvrBs2-mEGFP	Kan	pJK-B2-800p
pL0M-CDS1ns-AvrBs2	Spec	pJK-0-364
pL2M-P19-2x35S::PVX^CP4_CP_30-142-4xMyc	Kan	pJK-B2-259p
pL2M-P19-2x35S::PVX^CP4_CP_30-142-EGFP	Kan	pMC-B2-142p-mEGFP
pL0M-CDS1ns-NbNRC2a	Spec	pJK-0-116
pL0M-CDS1ns-NbNRC2a^L9E/L13E/L17E	Spec	pJK-0-117
pL0M-CT-EGFP	Spec	pICSL50044
pL0M-CDS1ns-mCherry	Spec	pJK-0-071
pL0M-CDS1-RpiBlb2	Spec	pICSL80031
pCR8-CDS1ns-Rx	Spec	pCR8-Rx
pL0M-CT-3xFLAG	Spec	pICSL50007
pL0M-CT-6xHA	Spec	pICSL50009A
pL0M-CT-4xMyc	Spec	pICSL50010
pL1V2-P19/MCS	Kan/mRFP1	pJK268c_mRFP1
pL0M-P5U-2x35S-TMV	Spec	pICH51288
pL0M-3UT-t35S	Spec	pICH41414
pICH41155-DraIII	Spec	pICH41155

Product	Purpose	RE	Plasmids
pL2M-P19-2x35S::NbNRC2a-4xMyc	Binary Agro L2M	BsaI	pJK268c_mRFP1, pICH51288, pICH41414, pICSL50010, pJK-0-116
pL2M-P19-2x35S::Rx-6xHA	Binary Agro L2M	BsaI	pJK268c_mRFP1, pICH51288, pICH41414, pICSL50009A, pCR8-Rx
pL2M-P19-2x35S::Bs2-6xHA	Binary Agro L2M	BsaI	pJK268c_mRFP1, pICH51288, pICH41414, pICSL50009A
pL2M-P19-2x35S::NbNRC2a^L9E/L13E/L17E-3xFLAG	Binary Agro L2M	BsaI	pJK268c_mRFP1, pICH51288, pICH41414, pICSL50007, pJK-0-117
pL2M-P19-2x35S::NbNRC2a^L9E/L13E/L17E-4xMyc	Binary Agro L2M	BsaI	pJK268c_mRFP1, pICH51288, pICH41414, pICSL50010, pJK-0-117
pL2M-P19-2x35S::NbNRC2a-mEGFP	Binary Agro L2M	BsaI	pJK268c_mRFP1, pICH51288, pICH41414, pICSL50034, pJK-0-116
pL2M-P19-2x35S::NbNRC4^L9A/V10A/L14A-4xMyc	Binary Agro L2M	BsaI	pICH86988, pICSL50010, pCR8-NRC4AAA
pL2M-P19-2x35S::NbNRC2a^L9E/L13E/L17E-mEGFP	Binary Agro L2M	BsaI	pJK268c_mRFP1, pICH51288, pICH41414, pICSL50010, pJK-0-117
pL2M-P19-2x35S::Rpiblb2-6xHA	Binary Agro L2M	BsaI	pJK268c_mRFP1, pICH51288, pICH41414, pICSL50009A, pICSL80031
pL2M-P19-2x35S::EGFP	Binary Agro L2M	BsaI	pJK268c_mRFP1, pICH51288, pICH41414, pJK635
pL2M-P19-2x35S::NbNRC2a-mCherry-4xMYC	Binary Agro L2M	BsaI	pJK268c_mRFP1, pICH51288, pICH41414, pJK-0-071, pJK-0-116
pL2M-P19-2x35S::NbNRC2a^L9E/L13E/L17E-mCherry-4xMYC	Binary Agro L2M	BsaI	pJK-0-071, pICSL50010
pL2M-P19-2x35S::Rx-mCherry-6xHA	Binary Agro L2M	BsaI	pJK-0-071, pICSL50009A
pL2M-P19-2x35S::AvrBs2-mEGFP	Binary Agro L2M	BsaI	pJK268c_mRFP1, pICH51288, pICH41414, pICSL50044, pJK-0-364
pL0M-CDS1ns-AvrBs2	General L0M	DraIII	pICH41155
pL2M-P19-2x35S::PVX^CP4_CP_30-142-4xMyc	Binary Agro L2M	BsaI	pJK268c_mRFP1, pICH51288, pICH41414, pICSL50010
pL2M-P19-2x35S::PVX^CP4_CP_30-142-EGFP	Binary Agro L2M	BsaI	pJK268c_mRFP1, pICH51288, pICH41414, pICSL50044
pL0M-CDS1ns-NbNRC2a	General L0M	-	-
pL0M-CDS1ns-NbNRC2a^L9E/L13E/L17E	General L0M	BsaI	pICSL01005
pL0M-CT-EGFP	General L0M	-	-
pL0M-CDS1ns-mCherry	General L0M	-	-
pL0M-CDS1-RpiBlb2	General L0M	-	-
pCR8-CDS1ns-Rx	General L0M	-	-
pL0M-CT-3xFLAG	General L0M	-	-
pL0M-CT-6xHA	General L0M	-	-
pL0M-CT-4xMyc	General L0M	-	-
pL1V2-P19/MCS	Cloning	-	pICSL4723, pJK263, pICH47742_mRFP1, pICH41744
pL0M-P5U-2x35S-TMV	General L0M	-	-
pL0M-3UT-t35S	General L0M	-	-
pICH41155-DraIII	Cloning	-	-

Product	Template	Primers/Synthesis
pL2M-P19-2x35S::NbNRC2a-4xMyc	-	-
pL2M-P19-2x35S::Rx-6xHA	-	-
pL2M-P19-2x35S::Bs2-6xHA	pMD1-Bs2-HA	
pL2M-P19-2x35S::NbNRC2a^L9E/L13E/L17E-3xFLAG	-	-
pL2M-P19-2x35S::NbNRC2a^L9E/L13E/L17E-4xMyc		
pL2M-P19-2x35S::NbNRC2a-mEGFP		
pL2M-P19-2x35S::NbNRC4^L9A/V10A/L14A-4xMyc		
pL2M-P19-2x35S::NbNRC2a^L9E/L13E/L17E-mEGFP	-	-
pL2M-P19-2x35S::Rpiblb2-6xHA	-	-
pL2M-P19-2x35S::EGFP	-	-
pL2M-P19-2x35S::NbNRC2a-mCherry-4xMYC		
pL2M-P19-2x35S::NbNRC2a^L9E/L13E/L17E-mCherry-4xMYC	pJK-B2-252p	TTGGTCTCAGCTTCTCTAGCTAGAGTCGATC/TTGGTCTCACATTCCACCGAGATCGGGAGGGAATATAGAG
pL2M-P19-2x35S::Rx-mCherry-6xHA	pJK-B2-178p-HA	TTGGTCTCAGCTTCTCTAGCTAGAGTCGATC/TTGGTCTCACCATTCCACCCTCGACATTATTGCG
pL2M-P19-2x35S::AvrBs2-mEGFP	-	-
pL0M-CDS1ns-AvrBs2	-	
pL2M-P19-2x35S::PVX^CP4_CP_30-142-4xMyc	-	
pL2M-P19-2x35S::PVX^CP4_CP_30-142-EGFP	-	
pL0M-CDS1ns-NbNRC2a	-	-
pL0M-CDS1ns-NbNRC2a^L9E/L13E/L17E	pJK-0-116	
pL0M-CT-EGFP	-	-
pL0M-CDS1ns-mCherry	pJK636	TTGAAGACAATTCGTGAGACCACGAAGTGGCTC/TTGAAGACAACGAACCCTTGTACAGCTCGTCCATGC
pL0M-CDS1-RpiBlb2	-	-
pCR8-CDS1ns-Rx	-	-
pL0M-CT-3xFLAG	-	-
pL0M-CT-6xHA	-	-
pL0M-CT-4xMyc	-	-
pL1V2-P19/MCS	-	-
pL0M-P5U-2x35S-TMV	-	-
pL0M-3UT-t35S	-	-
pICH41155-DraIII	-	-

Product	Comment	Publication	OD
pL2M-P19-2x35S::NbNRC2a-4xMyc	NRC2-Myc	This work	0.3
pL2M-P19-2x35S::Rx-6xHA	Rx-HA	This work	0.3
pL2M-P19-2x35S::Bs2-6xHA	Bs2-HA	This work	0.3
pL2M-P19-2x35S::NbNRC2a^L9E/L13E/L17E-3xFLAG	NRC2EEE-FLAG	This work	0.3
pL2M-P19-2x35S::NbNRC2a^L9E/L13E/L17E-4xMyc	NRC2EEE-Myc	This work	0.3
pL2M-P19-2x35S::NbNRC2a-mEGFP	NRC2-mEGFP	This work	0.3
pL2M-P19-2x35S::NbNRC4^L9A/V10A/L14A-4xMyc	NRC4AAA-4xMyc	This work	0.3
pL2M-P19-2x35S::NbNRC2a^L9E/L13E/L17E-mEGFP	NRC2EEE-GFP	This work	0.3
pL2M-P19-2x35S::Rpiblb2-6xHA	Rpiblb2-HA	This work	0.3
pL2M-P19-2x35S::EGFP	EGFP	Kourelis, Marchal et al., 2021	0.2
pL2M-P19-2x35S::NbNRC2a-mCherry-4xMYC	NRC2-mCherry-Myc	This work	0.3
pL2M-P19-2x35S::NbNRC2a^L9E/L13E/L17E-mCherry-4xMYC	NRC2EEE-mCherry-Myc	This work	0.3
pL2M-P19-2x35S::Rx-mCherry-6xHA	Rx-mCherry-HA	This work	0.3
pL2M-P19-2x35S::AvrBs2-mEGFP	AvrBs2-EGFP; GeneArt codon-optimized for *Nicotiana benthamiana*	This work	0.2
pL0M-CDS1ns-AvrBs2	GeneArt codon-optimized for *Nicotiana benthamiana*	This work	-
pL2M-P19-2x35S::PVX^CP4_CP_30-142-4xMyc	CP-Myc; GeneArt codon-optimized for *Nicotiana benthamiana*	This work	0.2
pL2M-P19-2x35S::PVX^CP4_CP_30-142-EGFP	CP-EGFP; GeneArt codon-optimized for *Nicotiana benthamiana*	This work	0.2
pL0M-CDS1ns-NbNRC2a	-	Kourelis, Contreras et al., 2021	-
pL0M-CDS1ns-NbNRC2a^L9E/L13E/L17E	-	This work	-
pL0M-CT-EGFP	-	TSL SynBio	-
pL0M-CDS1ns-mCherry	-	This work	-
pL0M-CDS1-RpiBlb2	-	TSL SynBio	-
pCR8-CDS1ns-Rx	-	Adachi et al., 2019	-
pL0M-CT-3xFLAG	-	Engler et a., 2014; Addgene #50308	-
pL0M-CT-6xHA	-	TSL SynBio	-
pL0M-CT-4xMyc	-	Engler et a., 2014; Addgene #50310	-
pL1V2-P19/MCS	pICSL4723 acceptor backbone with p19 module fixed in R1 position, clone into F2 using Bsa	Kourelis et al., 2020	-
pL0M-P5U-2x35S-TMV	-	Engler et a., 2014; Addgene #50269	-
pL0M-3UT-t35S	-	Engler et a., 2014; Addgene #50337	-
pICH41155-DraIII	General L0M acceptor, DraIII	TSL SynBio	-
