# Supplementary figures and images for: Sensor NLR immune proteins activate oligomerization of their NRC helpers in response to plant pathogens
Source: EMBO J. 2022 Dec 29;42(5):e111519. doi: 10.15252/embj.2022111519 (PMC9975940; doi:10.15252/embj.2022111519)

Figure 1 Source Data

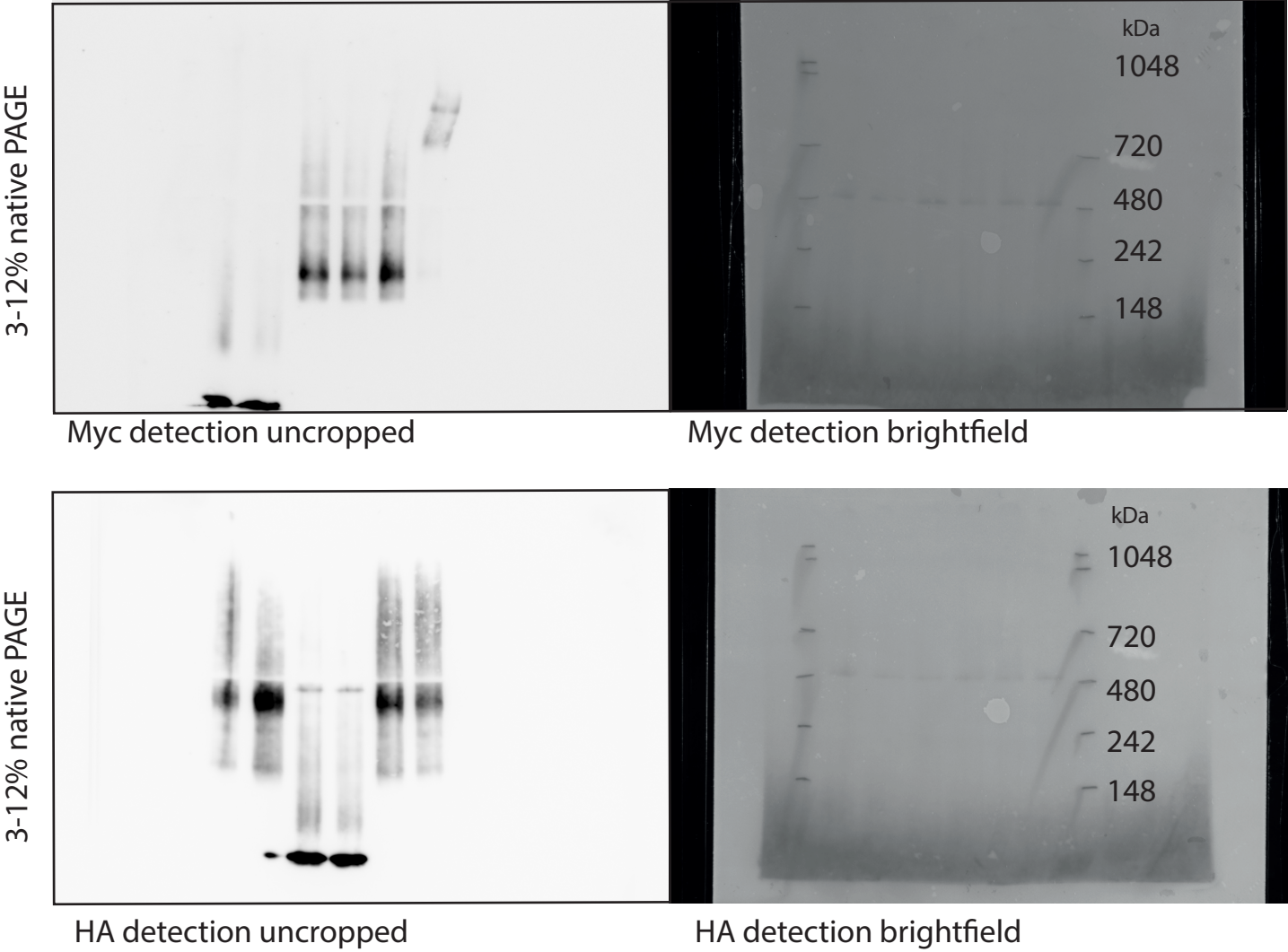

Figure 1 Source Data

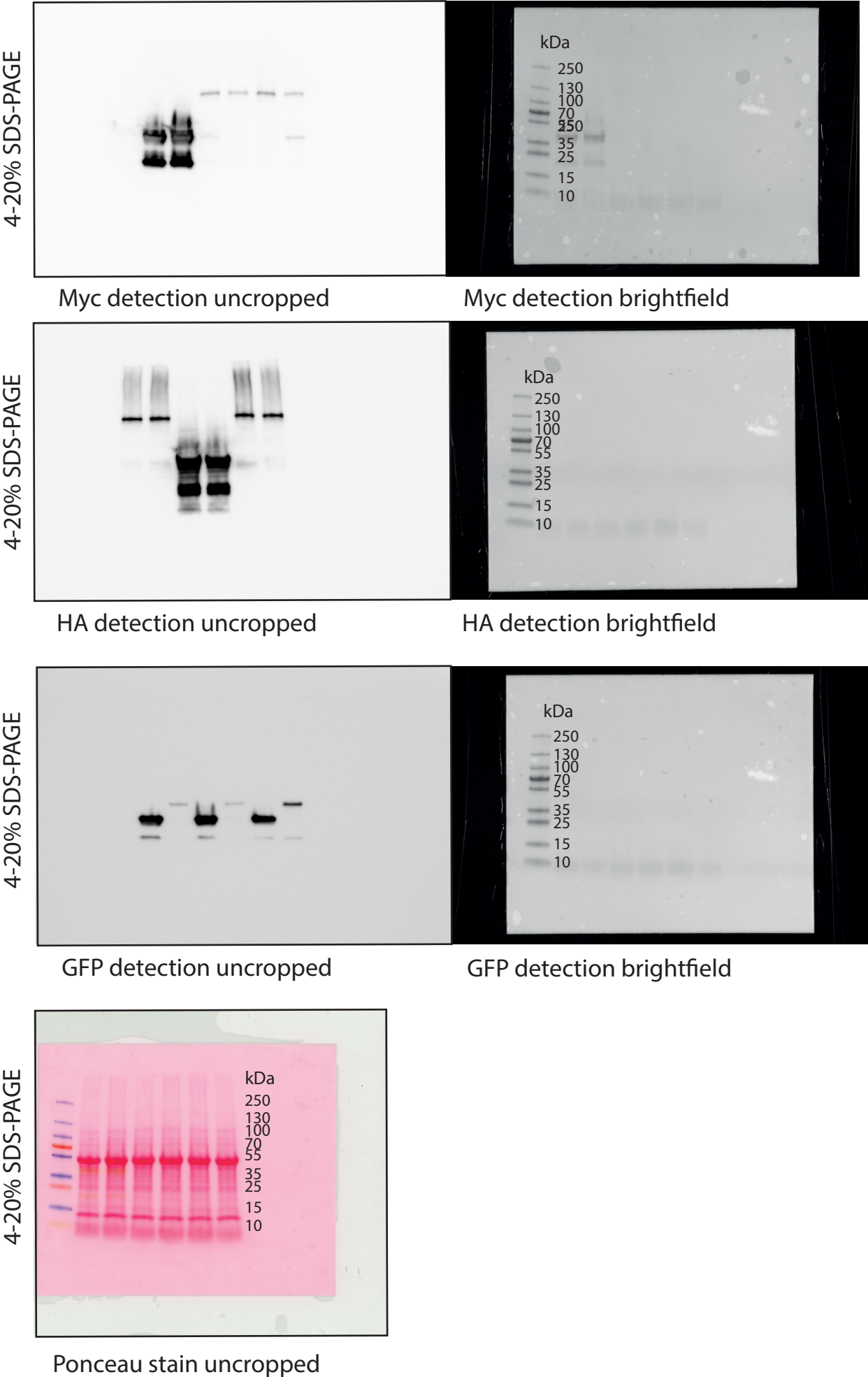

Supplement: Supplementary file 6 — Source Data for Figure 1 [file EMBJ-42-e111519-s001.zip › SD-Fig1.pdf]

Figure 6 Source Data

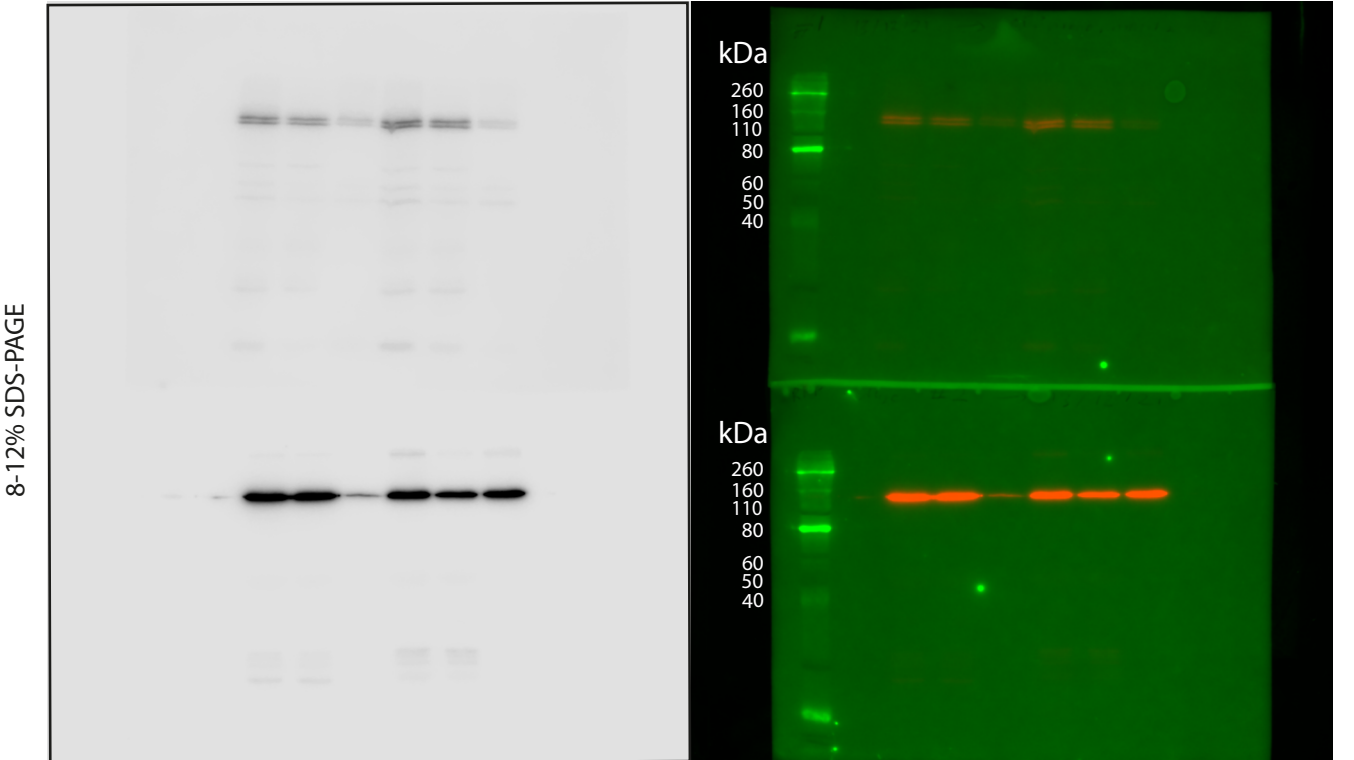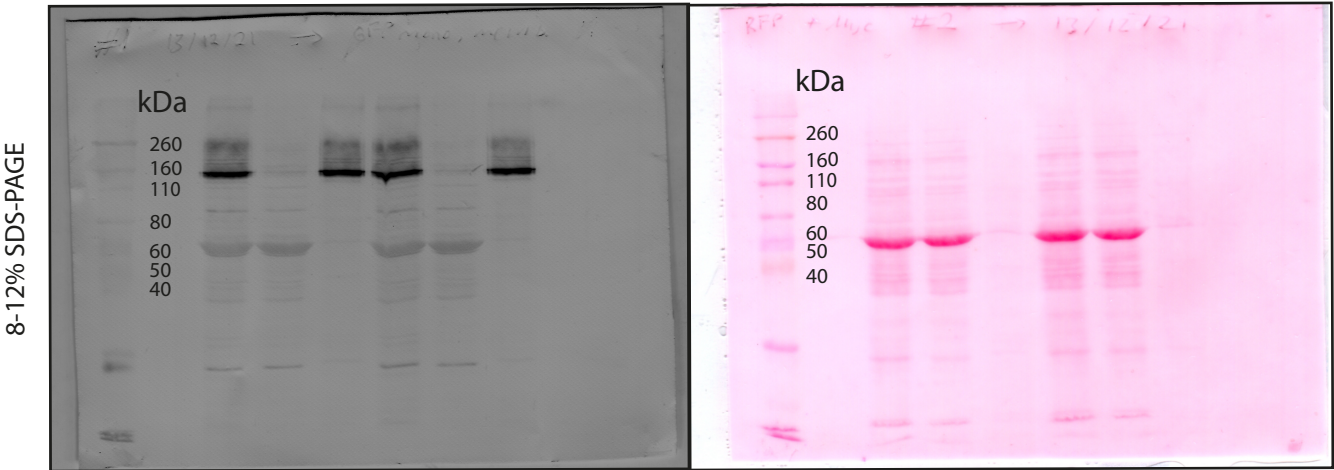

Supplement: Supplementary file 11 — Source Data for Figure 6 [file EMBJ-42-e111519-s005.zip › SD-Fig6.pdf]

Figure 7 Source Data

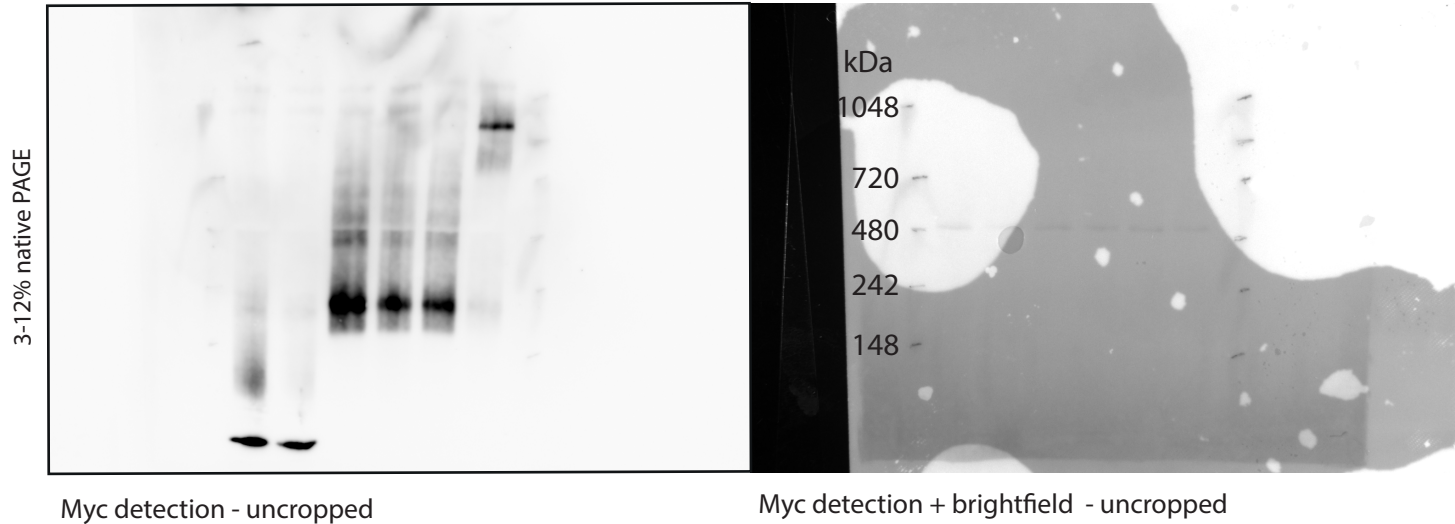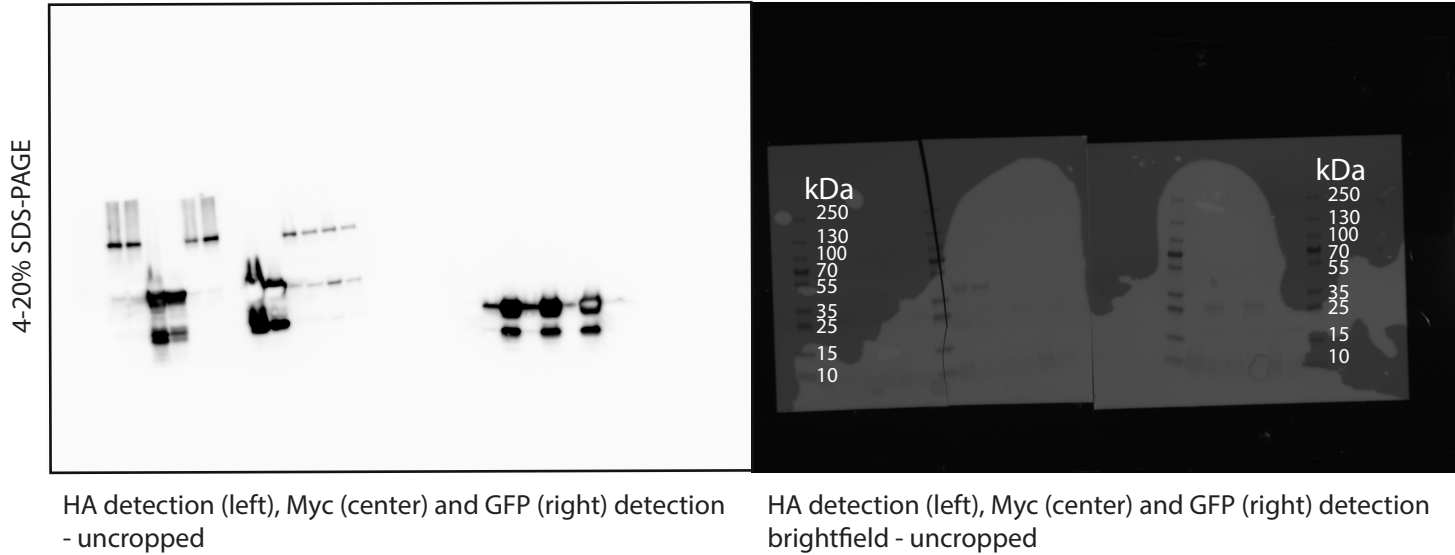

Supplement: Supplementary file 12 — Source Data for Figure 7 [file EMBJ-42-e111519-s006.zip › SD-Fig7.pdf]
