# Supplementary material for: Sensor NLR immune proteins activate oligomerization of their NRC helpers in response to plant pathogens
Source: EMBO J. 2022 Dec 29;42(5):e111519. doi: 10.15252/embj.2022111519 (PMC9975940; doi:10.15252/embj.2022111519)
Supplement: Supplementary file 7 — Source Data for Figure 2 [file EMBJ-42-e111519-s013.zip › SD-Fig2.pdf]

Figure 2 Source Data

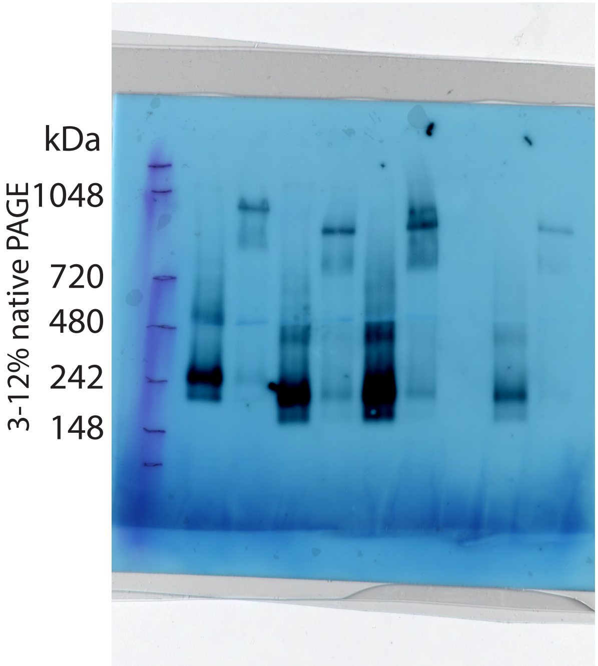

Myc detection + brightfield merge uncropped

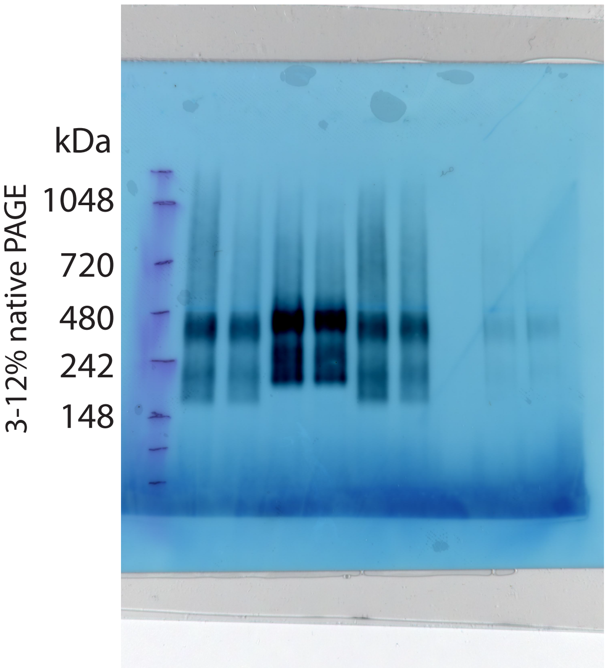

HA detection + brightfield merge uncropped

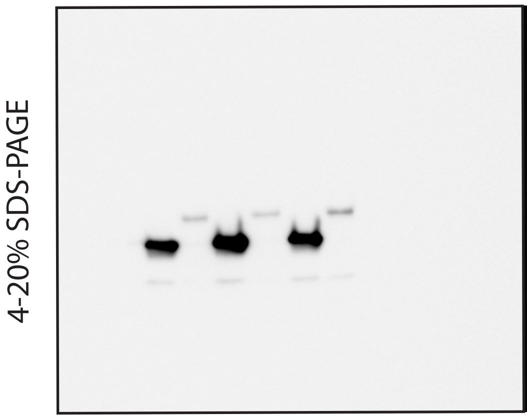

GFP detection uncropped

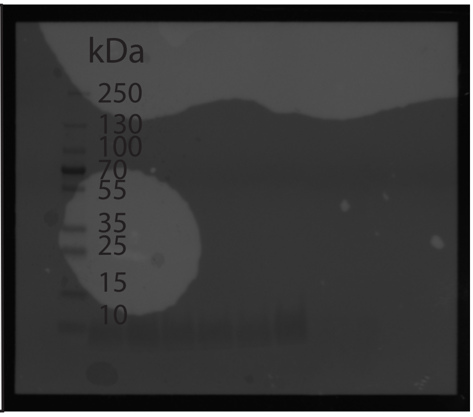

GFP detection brightfield

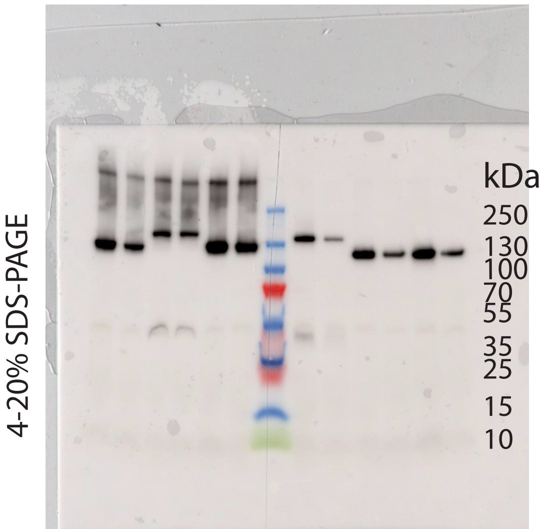

HA (left) and myc (right) detection + brightfield merge uncropped

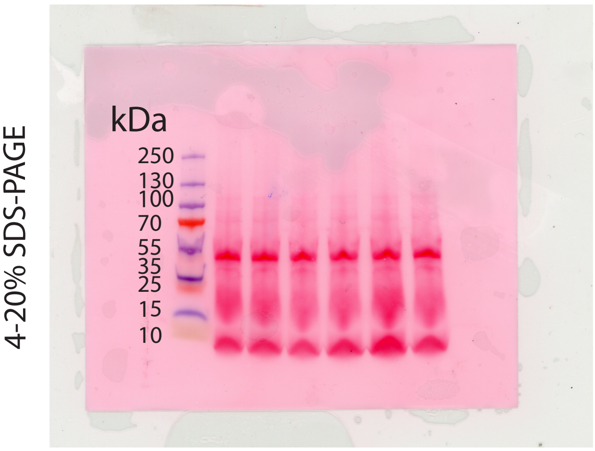

Ponceau stain uncropped
