# Supplementary material for: Sensor NLR immune proteins activate oligomerization of their NRC helpers in response to plant pathogens
Source: EMBO J. 2022 Dec 29;42(5):e111519. doi: 10.15252/embj.2022111519 (PMC9975940; doi:10.15252/embj.2022111519)
Supplement: Supplementary file 8 — Source Data for Figure 3 [file EMBJ-42-e111519-s007.zip › SD-Fig3.pdf]

Figure 3 Source Data

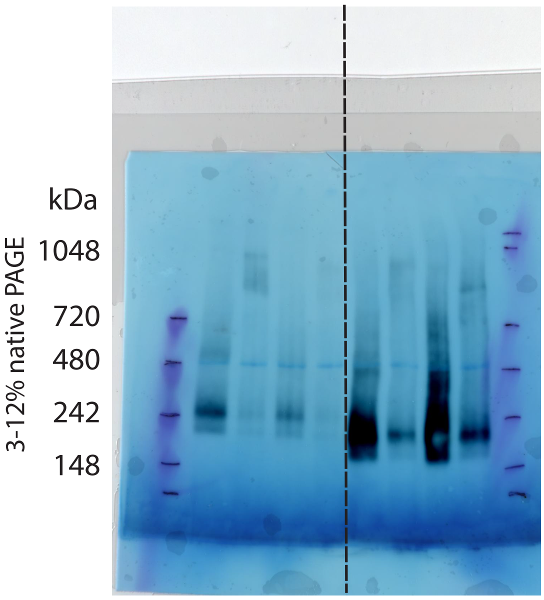

Myc detection (left) and FLAG detection (right)
+ brightfield merge - uncropped (low exposure)

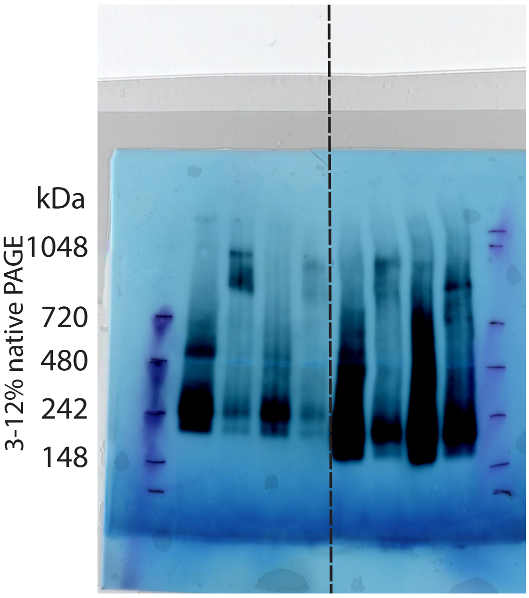

Myc detection (left) and FLAG detection (right)
+ brightfield merge - uncropped (high exposure)

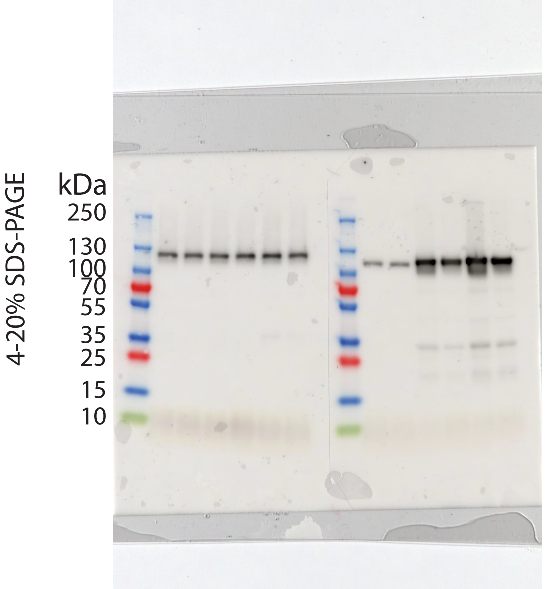

HA (left) and FLAG (right) detection
+ brightfield merge uncropped

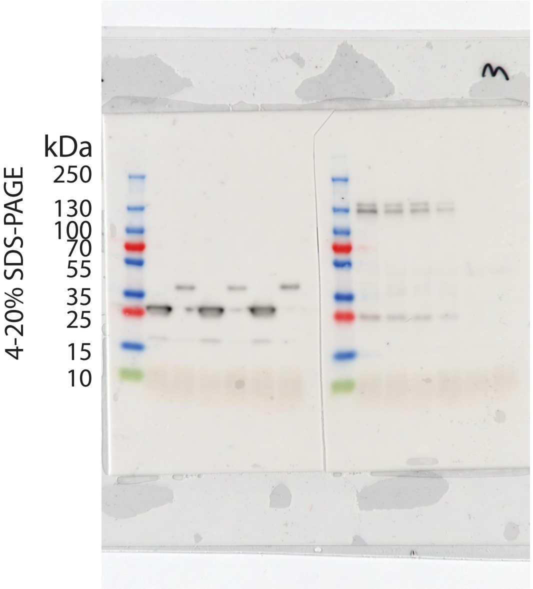

GFP (left) and Myc (right) detection
+ brightfield merge uncropped

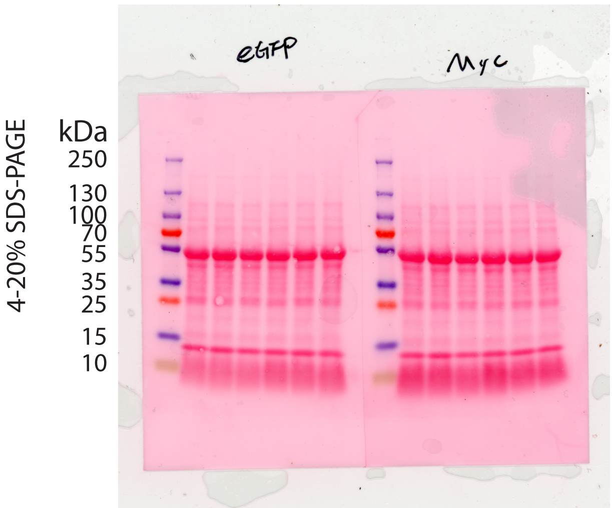

Ponceau Stain
