# Supplementary material for: Sensor NLR immune proteins activate oligomerization of their NRC helpers in response to plant pathogens
Source: EMBO J. 2022 Dec 29;42(5):e111519. doi: 10.15252/embj.2022111519 (PMC9975940; doi:10.15252/embj.2022111519)
Supplement: Supplementary file 9 — Source Data for Figure 4 [file EMBJ-42-e111519-s012.zip › SD-Fig4.pdf]

Figure 4 Source Data

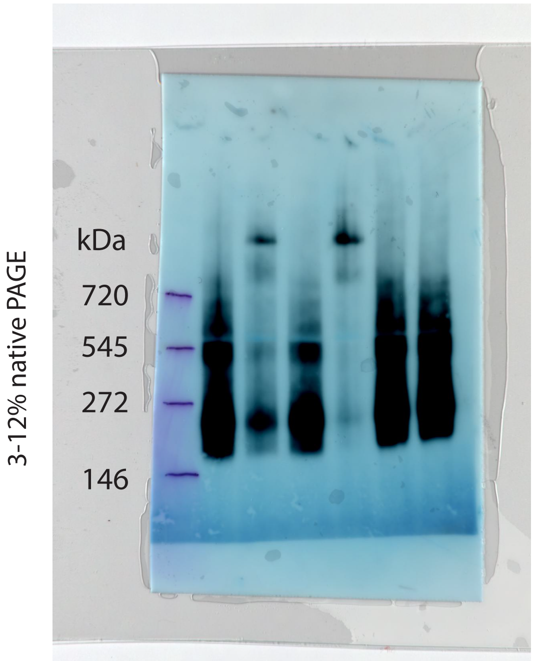

Myc detection + brightfield merge - uncropped

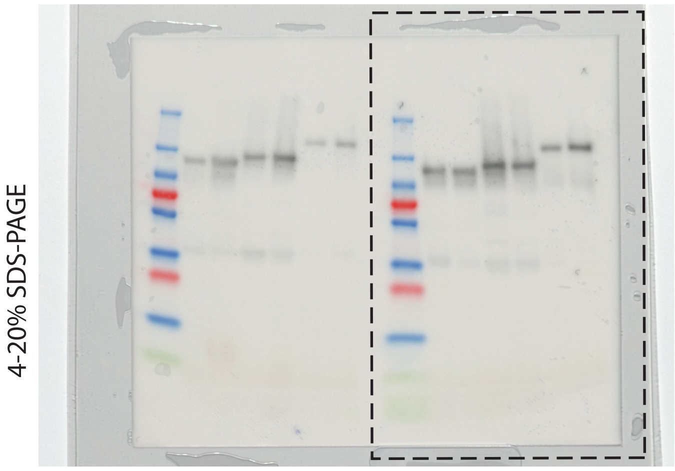

HA detection + brightfield merge - uncropped

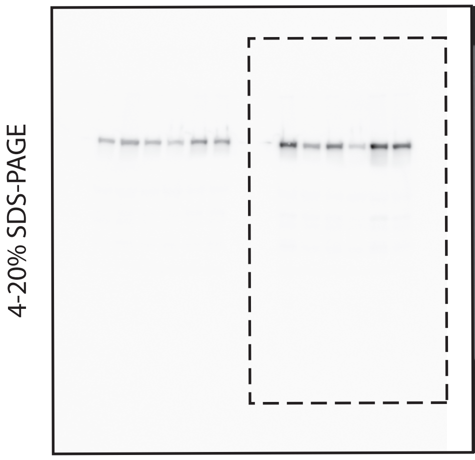

Myc detection - uncropped

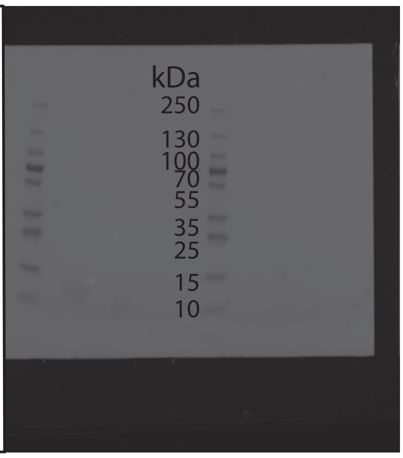

Myc detection - brightfield

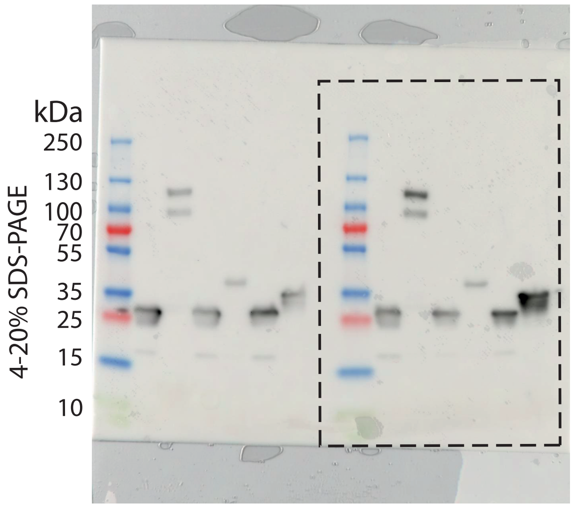

GFP detection + brightfield merge - uncropped

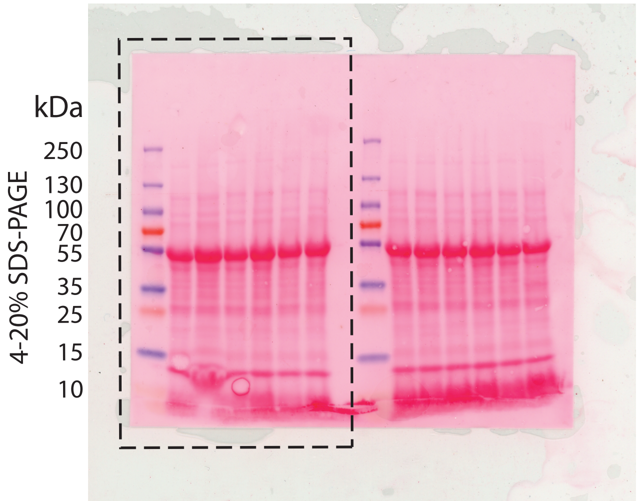

Ponceau stain
