# Supplementary material for: Sensor NLR immune proteins activate oligomerization of their NRC helpers in response to plant pathogens
Source: EMBO J. 2022 Dec 29;42(5):e111519. doi: 10.15252/embj.2022111519 (PMC9975940; doi:10.15252/embj.2022111519)
Supplement: Supplementary file 10 — Source Data for Figure 5 [file EMBJ-42-e111519-s010.zip › SD-Fig5.pdf]

Figure 5 Source Data

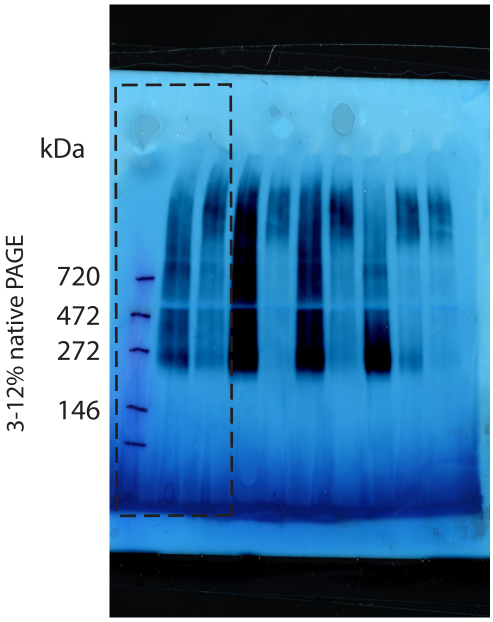

Myc detection + brightfield merge - uncropped

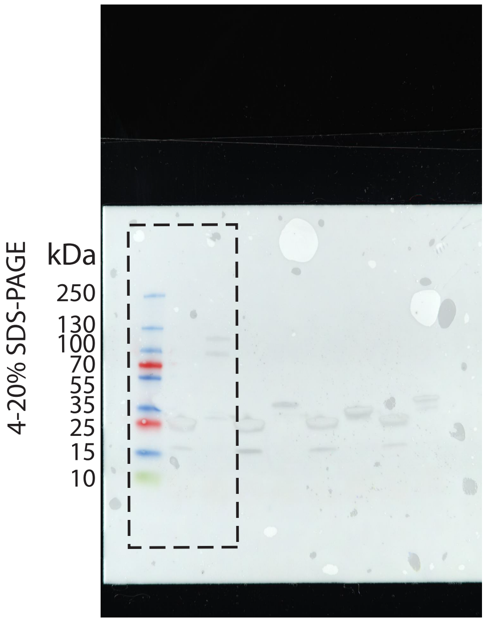

GFP detection + brightfield merge - uncropped

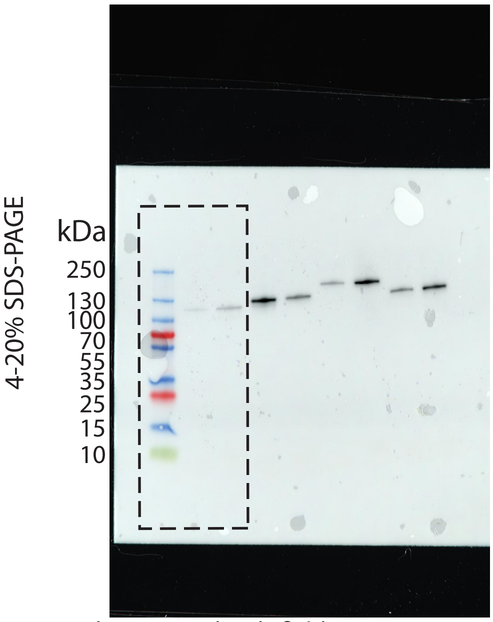

HA detection + brightfield merge - uncropped

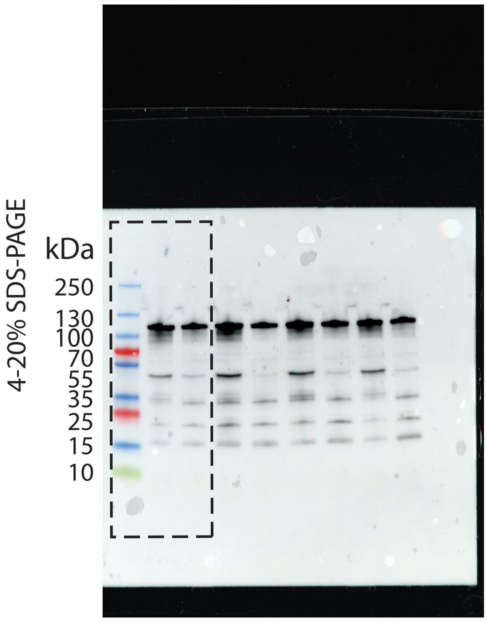

Myc detection + brightfield merge - uncropped

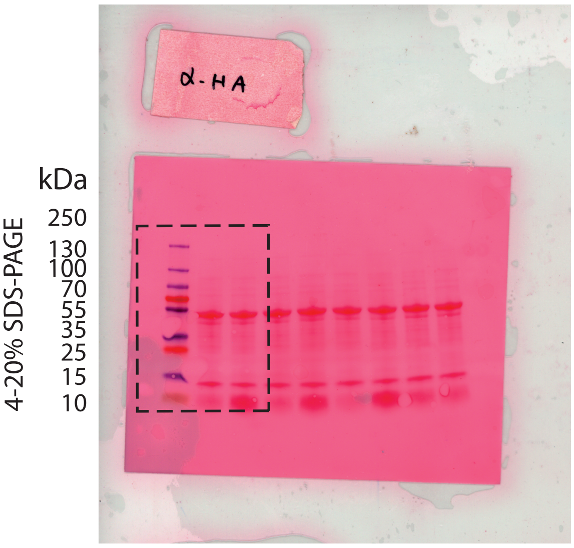

Ponceau Stain

Figure 5 Source Data

3-12% native PAGE

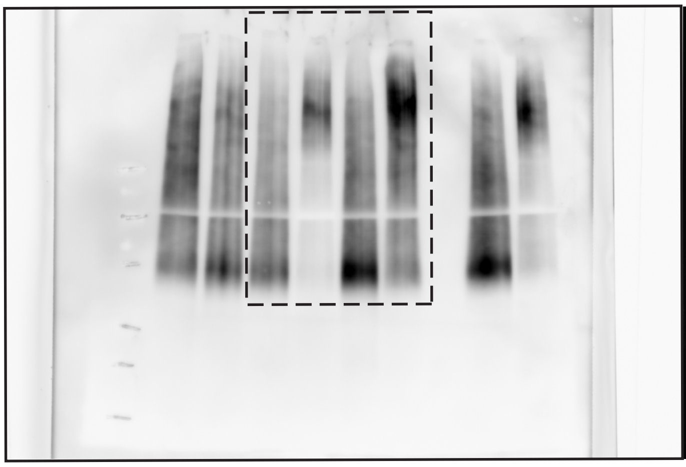

Myc detection - uncropped

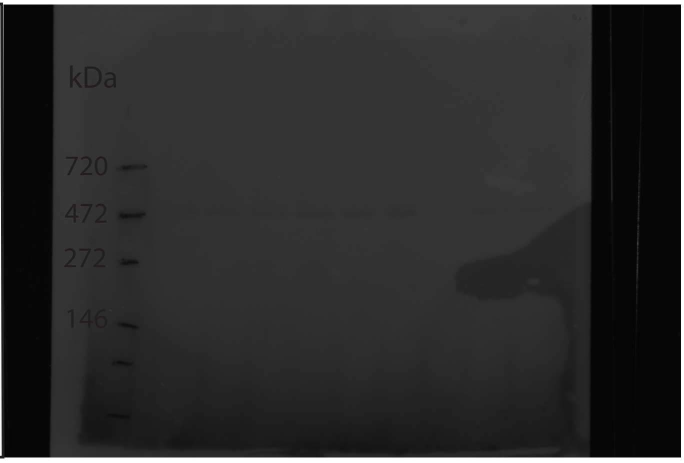

Myc detection - brightfield

4-20% SDS-PAGE

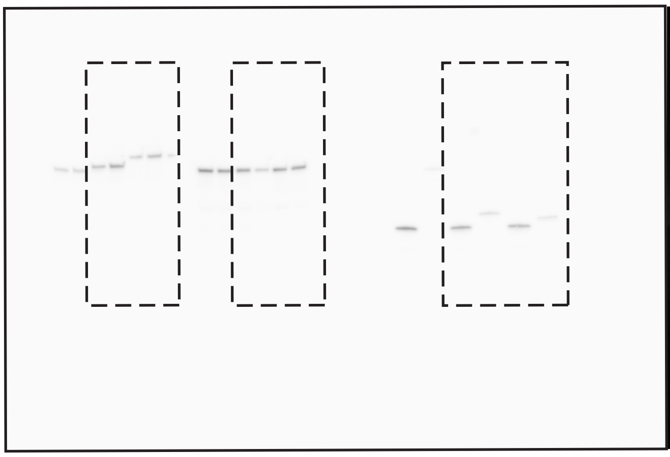

HA (left) Myc (center) and GFP (right) detection - uncropped

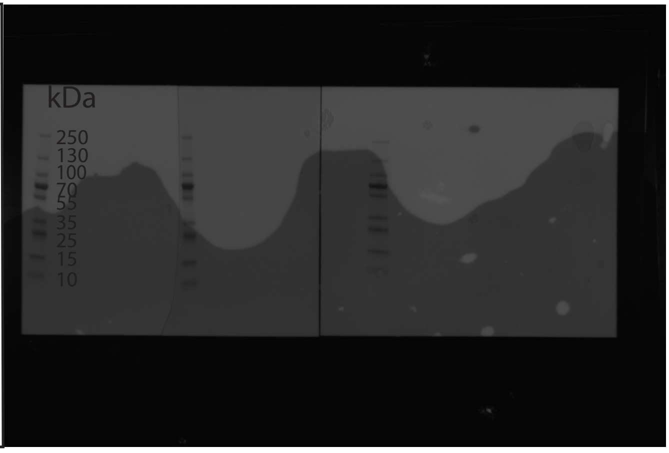

HA (left) Myc (center) and GFP (right) brightfield

4-20% SDS-PAGE

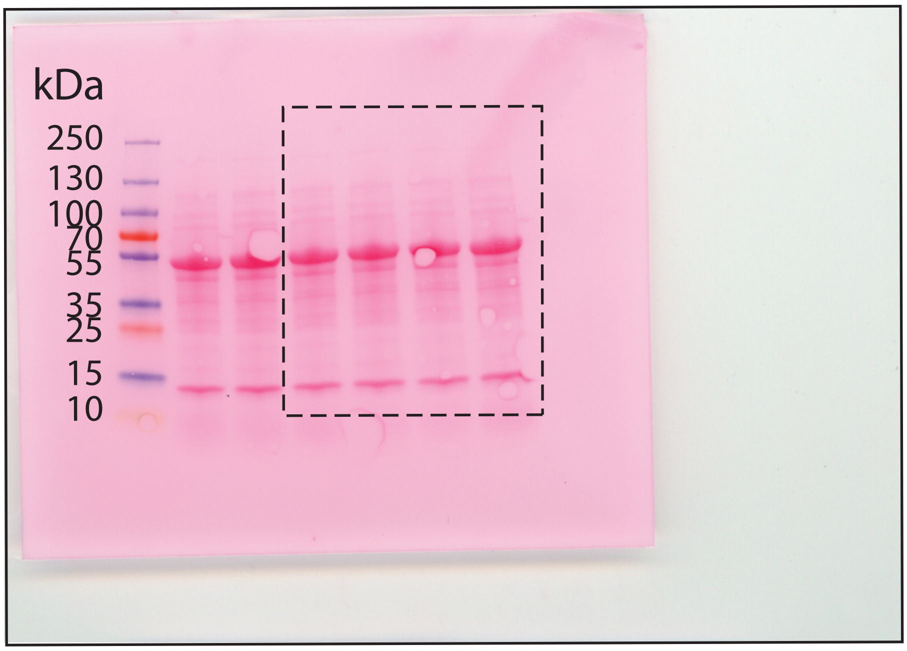

Ponceau Stain
