# Supplementary material for: Sensor NLR immune proteins activate oligomerization of their NRC helpers in response to plant pathogens
Source: EMBO J. 2022 Dec 29;42(5):e111519. doi: 10.15252/embj.2022111519 (PMC9975940; doi:10.15252/embj.2022111519)
Supplement: Supplementary file 13 [file EMBJ-42-e111519-s002.pdf]

THE EMBO JOURNAL

Sensor NLR immune proteins activate oligomerization of their NRC helpers in response to plant pathogens

Mauricio Contreras, Hsuan Pai, Yasin Tumtas, Cian Duggan, Enoch Lok Him Yuen, Angel Vergara Cruces, Jiorgos Kourelis, Hee-Kyung Ahn, Kim-Teng Lee, Chih-Hang Wu, Tolga Bozkurt, Lida Derevnina, and Sophien Kamoun
DOI: 10.15252/embj.2022111519

Corresponding author(s): Sophien Kamoun (sophien.kamoun@tsl.ac.uk) , Lida Derevnina (ld645@cam.ac.uk)

Review Timeline:

Submission Date:	26th Apr 22
Editorial Decision:	21st Jun 22
Revision Received:	30th Sep 22
Editorial Decision:	2nd Nov 22
Revision Received:	21st Nov 22
Editorial Decision:	24th Nov 22
Revision Received:	25th Nov 22
Accepted:	2nd Dec 22

Editor: William Teale

Transaction Report:

(Note: With the exception of the correction of typographical or spelling errors that could be a source of ambiguity, letters and reports are not edited. Depending on transfer agreements, referee reports obtained elsewhere may or may not be included in this compilation. Referee reports are anonymous unless the Referee chooses to sign their reports.)

1st Editorial Decision 21st Jun 2022

Dear Prof. Kamoun,

Thank you for submitting your manuscript for consideration at the EMBO Journal. It has now been seen by three referees whose comments are shown below.

Before deciding whether to moving forward towards publication of your manuscript, I would very much like to discuss the referee reports with you over Zoom. This will help me judge whether the referees are asking for an achievable set of revisions. I am away at a conference next week, but will be available from the following Monday. Alternatively, I can offer a meeting on Thursday morning (23rd June). I understand this gives you a very limited amount of time to digest the reports; however, I am mindful that you might want to accelerate the editorial process after the longer time taken by a back-to-back submission of this kind.

I have sent a duplicate of this email to Dr Jones (but including the referee reports for his manuscript).

Yours sincerely,

William Teale

William Teale, PhD
Editor
The EMBO Journal
w.teale@embojournal.org

Referee #1:

General comments
This study describes the mechanism of helper NLR activation by two different sensor NLRs. A detailed understanding of this mechanism is required to ultimately engineer disease resistance. Authors show that sensor NLRs Rx and Bs2 can recognize a specific effector and, in turn, trigger the oligomerization and plasma membrane localization of the helper NLR NRC2. These are particularly important information because these events have been shown to be key features of NLR activation. This suggests that NRC2 function similarly to ZAR1, Sr33 or NRG1.1, ie by forming a resistosome which may act as a cation channel. This study is well written, provides high quality data and increases our comprehension of the mechanisms underlying NLR activation. However, although it is clearly demonstrated that the NRC2 oligomer is composed of mostly NRC2 molecules, it is still possible that Rx forms a stable complex with NLRC4 with a stoichiometry of 1 Rx for 4 or more NLRC4. Since this information is a major part of the study, it is important to confirm whether Rx is part of the resistosome or not.

Major comments
I am a not fully convinced by the experiment in figure 2. If RX/NRC2 complex has a stoichiometry of 1 RX molecule and 4 (or more) NRC2 molecules, the shift in size on the blue native gel would be extremely small at sizes over 900kDa. The HA native PAGE shows that most of RX protein migrate at around 480 but there is a smear above. We can't be sure that RX is not part of a higher molecular weight complex with this experiment. A 2D page (native then SDS) should be done, like in the Ahn et al companion paper. Alternatively, NAIP2/NLRC4 could be shown as control. If you can see a size shift in NLRC4 in blue native page upon changing the tag from light to heavy in NAIP2, it would significantly strengthen your argument.

Minor comments
Figure 1B and following. GFP alone is used instead of CP-GFP throughout the study but it is not indicated on the figure. Please replace CP-GFP by GFP (-) or CP-GFP (+) on the figure to facilitate reading of the figures.

Additional suggestions:
I have one suggestion to improve the manuscript although it should not delay the publication. Authors show that NRC2 localize to the plasma membrane upon activation, further suggesting it can act as an ion channel like ZAR1, Sr33 or NRG1.1. Observing cytosolic calcium levels during infection is not very complex and would indicate if NRC2 regulates (directly or indirectly) calcium levels. In other organisms, NLR resistosomes can function through a different mechanism (as scaffolds for instance in mammals). I feel that it would be important to test if resistosome formation and calcium influx are conserved mechanisms in plant immunity.

Referee #2:

The manuscript by Mauricio P. Contreras et al., describes the oligomerization of the NRC2 helper by Rx and Bs2 sensor NLRs upon interaction with matching pathogen effectors. The authors show that Rx and Bs2, which function as sensor NLRs, can each trigger oligomerization of the NRC2 helper NLR but are not themselves part of the activated resistosome. Transient gene expression experiments in N. benthamiana leaves suggest PVX-triggered and Rx-dependent relocation of NRC2 to the plasma membrane. On the basis of these results, the authors present an activation-and-release model for the studied sensor-helper pairs.

General Comments and major points of concern

Extensive prior work has already been conducted on 'extreme resistance' (without cell death) mediated by the potato Rx NLR to PVX virus. How Rx and Bs2 NLRs transmit effector-dependent signals to the NRC2 helper NLR remains unresolved in the present study, and the stoichiometry of the postulated 'NRC2 resistosome' (Fig. 8) remains undetermined. Another limitation of the present work is the exclusive use of BN-PAGE to characterize NLR complexes, which is known to suffer from inherent limitations (e.g., estimation of complex size) and the lack of independent assays for complex characterization, such as routine size exclusion chromatography (SEC).

1. Formation and detection of the 'NRC2 resistosome' (>720 kDa) relies exclusively on the transient gene OE of a single tested inactive variant, designated NRC2EEE, in N. benthamiana leaves (Fig. 1 to 3). Whether WT NRC2 forms the same or a similar complex remains unclear. How can the authors exclude the possibility that the NRC2EEE complex accumulates in planta without associated Rx because NRC2EEE is not functional? In other words, the unexpected absence of Rx in the NRC2 complex could be due to the use of the non-functional NRC2EEE variant, which calls into question the main conclusion of the present work.

2. Rx is known to signal redundantly through NRC2/3/4 helper NLRs (Wu et al., 2017). If one assumes that the Rx-induced NRC2EEE complex is a proxy for a signalling NRC2 oligomer, then one would expect that co-expression of CP-GFP and Rx together with NRC3EEE or NRC4EEE would lead to the formation of similar high-order NRC complexes in planta. This is an explicit prediction of the genetic data and will test whether sensor NLR-triggered NRC complex formation is a shared biochemical feature of functionally redundant NRC2, NRC3 and NRC4.

3. Figure 1B The size of the monomeric C-terminally Rx-6xHA is expected to be ~115 kDa. In the BN-PAGE assays, Rx constitutively migrates as a band of ~400 kDa, regardless of its activation state. The authors propose that this ~400 kDa band could correspond to a preformed Rx complex with additional host proteins or a larger complex consisting of multiple Rx proteins. Does this complex contain RanGAP2, a previously identified interacting protein of Rx, which is required for extreme resistance to PVX and is also needed for Rx nucleocytoplasmic partitioning (Tameling et al., 2007 Plant Cell; Tameling et al., 2010 Plant Cell)? Unfortunately, this prior work is not mentioned in the current manuscript. In the companion paper by Hee-Kyung Ahn et al., Fig 1B, D, Rpi-amr1 and Rpi-amr3 when fused with HA migrates at a size ~400 kDa, while Flag-tagged versions of Rpi-amr1 or Rpi-amr3 migrate with a size of ~270 kDa when immunoprecipitated with Flag tag. Can the authors confirm that the slower migrating band observed for Rx-6xHA is not due to an artifact of the HA tag used in the above tests, but is an inherent property of Rx?

4. No evidence is provided that Rx and CP-GFP associate in planta. BN-PAGE (IB-GFP)/Co-immunoprecipitation data should be provided to test whether CP-GFP co-migrates with Rx-6xHA in vivo.

5. Figure 2B - In the BN-PAGE assays, in the absence of CP, when NRC2EEE is co-expressed with Rx, NRC2EEE (both -mCherry-4xMyc and -4xMyc tagged) migrates as a band of ~200 kDa and ~400 kDa. In the same figure, Rx also migrates as a band of ~400 kDa, coinciding with the slower migrating band of NRC2EEE. A similar migration pattern was observed in Figure S4 when Rx and NRC2EEE was tagged with C-terminal RFP and GFP tags, respectively. To address if Rx forms a complex with NRC2 before Rx activation, the authors performed the BN-PAGE assay with the "heavy" and "light" versions of the NRC2EEE and Rx and conclude that Rx and NRC2 do not form stable complexes with each other at resting state. Due to inherent limitations of BN-PAGE analysis, it is important to independently validate these results by co-immunoprecipitation assays. In addition, I strongly recommend that the authors independently validate the size of pre-activation Rx and activated NRC2EEE complexes by routine SEC experiments.

6. Figure 4. The authors nicely show that Bs2 activation with AvrBs2 is able to trigger NRC2EEE complex formation in a similar manner to Rx, whereas Rpi-blb2 activation with AVRblb2 did not lead to NRC2EEE oligomerization. To support the claim of the authors that "that sensor-mediated NRC oligomerization is a general principle for NRC dependent sensors" (lines 279 - 280), it is important to show that Rpi-blb2 activation results in NRC4 complex formation (e.g. NRC4EEE oligomer) as Rpi-blb2 is an NRC4-dependent sensor.

7. Figure 7. Both Rx and NRC2EEE display cytoplasmic localization in the pre-activated state. Previous studies by Slootweg et al., 2010, Tameling et al., 2010, found that nucleocytoplasmic partitioning of Rx is essential for effective defence signalling. Unfortunately, this prior work is not mentioned in the current manuscript. No nuclear localization of Rx in the activated state was described by the authors. If NRC2EEE oligomerization depends on transient interaction of activated Rx with NRC2EEE in the cytoplasm, no oligomerization of NRC2EEE is expected when localization of Rx in the nucleus is enforced. It would be very informative if the author could provide such evidence. In addition, can the authors exclude the possibility that Agrobacterium-mediated transient gene OE of the respective gene constructs in N. benthamiana leaves confounds meaningful interpretation of the observed subcellular localization of NRC2? Conclusive evidence could be provided by generating and testing transgenic NRC2EEE-GFP plants driven by native 5' regulatory sequences in the nrc2/3/4 mutant background.

8. The authors show that the induced NRC2EEE oligomers accumulate in membrane-associated puncta, but it remains unclear whether the mutant oligomer and/or WT-NRC2 oligomer have ion channel activity. This could be determined by electrophysiological experiments in oocytes, although this request appears to be beyond the scope of the present study. Nevertheless, because of the lack of direct evidence for NRC2 ion channel activity and lack of information on the stoichiometry of the NRC2EEE complex in the present work, it is inappropriate to refer to the NRC2EEE oligomer as 'the NRC2 resistosome'. Similarly, Fig. 8 should be removed because, as explained above, the activation-and-release model lacks compelling experimental evidence.

9. Rx-mediated immunity to PVX was originally described as "extreme resistance," i.e., without concomitant host cell death. It was then shown that Rx drives separate virus resistance and cell death responses (Bendahmane et al., 1999). In the present work, the biochemical basis of Rx-mediated immunity to PVX without cell death remains unclear. In Arabidopsis, it is now clear that two NLR signalling nodes act in parallel (EDS1-PAD4-ADR1 and EDS1-SAG101-NRG1), each mediating a potent immune response, but only the former is associated with host cell death. The use of transient gene OE experiments and their combination with cell death assays in N. benthamiana leaves throughout the present study carries the risk of overlooking one/several NRC2/Rx heterocomplex(es) without host cell death activity that are physiologically relevant and different from the NRC2EEE oligomer described.

Minor points

1. Line 184-186 - To achieve this, we transiently expressed these proteins by agroinfiltration in the absence or presence of PVX CP in leaves of nrc2/3/4 N.benthamiana CRISPR lines using agroinfiltration and subjected total protein extracts to BN-PAGE assays - Agroinfiltration occurs twice.

2. Line 146-148 - How activation of sensor NLRs translates into helper activation, immune signaling and disease resistance remains a fundamental question in plant immunology. Why this hyperbole? Please replace 'fundamental' by 'important'.

3. The introduction is very long for an original research article and should be drastically shortened.

4. I am not convinced by the terminology of sensor-helper NLR "networks" in the present work. From a biochemical point of view, the genetic data only point to different functional redundancies of NRC2/3/4 with the respective sensor NLRs. The term 'networked NLR immune receptors' should be removed.

Referee #3:

The work uses BN-PAGE to study the role of Rx and NRC activation by showing restistosome formation in Nicotiana benthamiana leaves. Induction of an effector PVX CP (GFP-tagged) induced oligomerisation of NRC2 to a size between 700-900 kDa in presence of sensor RX. Sensor RX and effector form also a complex, independent of NRC2 oligomer. Please indicate in an extra panel (Fig. 1) the composition of each complex and discuss the appearance of the complexes in the gel compared to the mass ladder (eg. using semilog plots). How many molecules can be included (stoichiometry).

Blots from native gels using size shift variants of RX and NRC show that NRC2 resistosome that does not include Rx. In addition size shifts were used to estimate the composition of oligomers. Please show in additional panel, how did you estimated this and how does this fit to the appearance in the gel.

Confocal live cell microscopy showed that upon effector activation tagged NRC2 proteins change their location from cytoplasm to the plasma membrane and organize in an ordered puncta distribution. This membrane associated complexes are separate from the sensor. Additional experiments using potato virus X infection as effector confirmed the NRC oligomerizatization and ordered membrane association.

The manuscript is clearly written and understandable, even for me being not in the field. I inspected blue native electrophoresis deeper and I am impressed about the excellent quality to show the dynamic resistosome formation.

Dear Dr. Teale,

Thank you very much for your evaluation of our manuscript "Sensor NLR immune proteins activate oligomerization of their NRC helpers" for consideration as a research article at The EMBO Journal.

We appreciate the overall positive feedback of the independent reviewers and have made the suggested textual changes. Please see the point-by-point 'Response to Reviewers' below.

We hope that we have addressed many of the issues that were raised, and we look forward to your response.

Best regards,

Sophien Kamoun
Group Leader and Professor

Referee #1:

General comments
This study describes the mechanism of helper NLR activation by two different sensor NLRs. A detailed understanding of this mechanism is required to ultimately engineer disease resistance. Authors show that sensor NLRs Rx and Bs2 can recognize a specific effector and, in turn, trigger the oligomerization and plasma membrane localization of the helper NLR NRC2. These are particularly important information because these events have been shown to be key features of NLR activation. This suggests that NRC2 function similarly to ZAR1, Sr33 or NRG1.1, ie by forming a resistosome which may act as a cation channel. This study is well written, provides high quality data and increases our comprehension of the mechanisms underlying NLR activation. However, although it is clearly demonstrated that the NRC2 oligomer is composed of mostly NRC2 molecules, it is still possible that Rx forms a stable complex with NLRC4 with a stoichiometry of 1 Rx for 4 or more NLRC4. Since this information is a major part of the study, it is important to confirm whether Rx is part of the resistosome or not.

Reviewer 1 major comments:

I am a not fully convinced by the experiment in figure 2. If RX/NRC2 complex has a stoichiometry of 1 RX molecule and 4 (or more) NRC2 molecules, the shift in size on the blue native gel would be extremely small at sizes over 900kDa. The HA native PAGE shows that most of RX protein migrate at around 480 but there is a smear above. **We can't be sure that Rx is not part of a higher molecular weight complex with this experiment. A 2D page (native then SDS) should be done, like in the Ahn et al companion paper.** Alternatively, NAIP2/NLRC4 could be shown as control. If you can see a size shift in NLRC4 in blue native page upon changing the tag from light to heavy in NAIP2, it would significantly strengthen your argument.

Response to major comments from reviewer 1:

To further test the hypothesis that Rx is absent in the high molecular weight NRC2 complex we now also include SEC gel filtration analyses with Rx and NRC2^{EEE} (**Figure EV2**) in the absence or presence of PVX CP. These assays described in lines 229-244 provide orthogonal validation that, upon activation, NRC2 exhibits a shift in elution volume indicative of complex formation while Rx exhibits no such shift and does not exhibit an elution pattern that overlaps with the high molecular weight NRC2 complex. This is in addition to the the previous orthogonal evidence provided in **Figures 2, 6** and **8**, e.g. the BN-PAGE results, lack of co-localization between activated Rx and activated NRC2^{EEE} in cell biology experiments and the differential membrane enrichment shown by activated Rx and activated NRC2^{EEE}. We believe that these data combined make a compelling case for the absence of Rx in the activated NRC2 complex.

Reviewer 1 minor comments:
Figure 1B and following. GFP alone is used instead of CP-GFP throughout the study but it is not indicated on the figure. Please replace CP-GFP by GFP (-) or CP-GFP (+) on the figure to facilitate reading of the figures.

Response to minor comments from reviewer 1:

Thank you for pointing this out. We have now added GFP on all relevant figures (**Figure 1, Figure 2, Figure 3, Figure 4, Figure 5 and Figure 7**) to facilitate reading.

Reviewer 1 additional suggestions:
I have one suggestion to improve the manuscript although it should not delay the publication. Authors show that NRC2 localize to the plasma membrane upon activation, further suggesting it can act as an ion channel like ZAR1, Sr33 or NRG1.1. **Observing cytosolic calcium levels during infection is not very complex and would indicate if NRC2 regulates (directly or indirectly) calcium levels.** In other organisms, NLR resistosomes can function through a different mechanism (as scaffolds for instance in mammals). I feel that it would be important to test if resistosome formation and calcium influx are conserved mechanisms in plant immunity.

Response to additional suggestions from reviewer 1:

We thank the reviewer for these suggestions. They would indeed strengthen the hypothesis that CC-NLR resistosomes function as calcium channels. However, these experiments aren't as straightforward as one might think because the activated NRC2 resistosome causes cell death in planta, and the EEE triple mutant might be unable to act as an ion channel (thus the lack of cell death). We are currently working on developing methods to test any ion channel activity of NRC2 using our experimental system, although it will not be possible to generate these data in the timeframe of this publication.

Referee #2:

The manuscript by Mauricio P. Contreras et al., describes the oligomerization of the NRC2 helper by Rx and Bs2 sensor NLRs upon interaction with matching pathogen effectors. The authors show that Rx and Bs2, which function as sensor NLRs, can each trigger oligomerization of the NRC2 helper NLR but are not themselves part of the activated resistosome. Transient gene expression experiments in *N. benthamiana* leaves suggest PVX-triggered and Rx-dependent relocation of NRC2 to the plasma membrane. On the basis of these results, the authors present an activation-and-release model for the studied sensor-helper pairs.

General Comments and major points of concern

Extensive prior work has already been conducted on 'extreme resistance' (without cell death) mediated by the potato Rx NLR to PVX virus. **How Rx and Bs2 NLRs transmit effector-dependent signals to the NRC2 helper NLR remains unresolved in the present study, and the stoichiometry of the postulated 'NRC2 resistosome' (Fig. 8) remains undetermined.** Another limitation of the present work is the exclusive use of BN-PAGE to characterize NLR complexes, **which is known to suffer from inherent limitations (e.g., estimation of complex size) and the lack of independent assays for complex characterization, such as routine size exclusion chromatography (SEC)**.

Reviewer 2 major comment 1

1. Formation and detection of the 'NRC2 resistosome' (>720 kDa) relies exclusively on the transient gene OE of a single tested inactive variant, designated NRC2EEE, in *N. benthamiana* leaves (Fig. 1 to 3). Whether WT NRC2 forms the same or a similar complex remains unclear. **How can the authors exclude the possibility that the NRC2EEE complex accumulates in planta without associated Rx because NRC2EEE is not functional?** In other words, the unexpected absence of Rx in the NRC2 complex could be due to the use of the non-functional NRC2EEE variant, which calls into question the main conclusion of the present work.

Response to reviewer 2 major comment 1:

We appreciate the reviewers' concern. Although NLRs have been identified in the early 1990s, the biochemical characterization of the activated state of these proteins has remained elusive until recently, in large part because of the cell death phenotype they cause *in planta* and in other experimental systems, such as insect cells and HeLa cells (Förderer et al. 2022, Jacob et al. 2021). Therefore, a major advance in this work is the use of the EEE mutant of NRC2 in the previously discovered MADA motif (Adachi et al. 2019) which abolishes the cell death without interfering with the oligomerization.

Nonetheless, we have now included BN-PAGE data with wild-type NRC2 carrying an intact N-terminus (**Figure EV1**) which we describe in lines 201-208. This experiment is a good

example of the issues that accompany working with wild-type activated NLRs, as the accumulation of all proteins was reduced in treatments with NRC2 when compared to treatments with NRC2^{EEE}, likely due to the induction of cell death. Nonetheless, we were able to visualize both Rx and activated NRC2, observing migration patterns similar to those seen for NRC2^{EEE}. This suggests that the shifts in size observed for NRC2^{EEE} and the lack of a band shift observed for Rx are not artifacts induced by the MADA-motif mutations.

In addition, the use of the NRC2^{EEE} protein is also justified because several previous studies on CC-type NLRs have successfully leveraged N-terminal mutations and shown that they are dispensable for oligomerization and subcellular re-localization upon activation (Wang et al. 2019, Förderer et al. 2022, Duggan et al. 2021, Hu et al., 2020) which is in line with our findings with NRC2^{EEE}. Recent papers (Hu et al., 2022, Zhao et al., 2022) show that truncated immune receptor variants in which the N-terminal a1-helix containing the MADA motif is completely removed retain the capacity to re-localize and oligomerize. In the last year, two structures of the activated Sr35 complex were solved both with and without N-terminal MADA motif mutations (Förderer et al. 2022 and Zhao et al. 2022), showing that these mutations have no effect on the overall structure or assembly of the pentameric Sr35 resistosome complex We believe that using N-terminal MADA-motif mutants for biochemical and cell biology assays is the state of the art and indeed our data suggest that NRC2^{EEE} does oligomerize and re-localizes to the plasma membrane after activation.

Reviewer 2 major comment 2

2. Rx is known to signal redundantly through NRC2/3/4 helper NLRs (Wu et al., 2017). If one assumes that the Rx-induced NRC2EEE complex is a proxy for a signalling NRC2 oligomer, then **one would expect that co-expression of CP-GFP and Rx together with NRC3EEE or NRC4EEE would lead to the formation of similar high-order NRC complexes in planta.** This is an explicit prediction of the genetic data and will test whether sensor NLR-triggered NRC complex formation is a shared biochemical feature of functionally redundant NRC2, NRC3 and NRC4.

Response to reviewer 2 major comment 2:

Thank you for this suggestion. We have now included new data in **Figure 5** showing that Bs2, Rx and Rpi-blb2 are able to mediate oligomerization of another helper NLR, NRC4 (Lines 316-325). To carry out these experiments, we leveraged a previously published N-terminal MADA motif mutant of NRC4, NRC4^{AAA}, to enable biochemical analyses (Adachi et al., 2019). These BN-PAGE assays reveal that oligomerization is a shared biochemical feature of NRC2 and NRC4 and that the genetic data can be recapitulated in our biochemical assays.

Reviewer 2 major comment 3.1

3. Figure 1B The size of the monomeric C-terminally Rx-6xHA is expected to be ~115 kDa. In the BN-PAGE assays, Rx constitutively migrates as a band of ~400 kDa, regardless of its activation state. The authors propose that this ~400 kDa band could correspond to a preformed Rx complex with additional host proteins or a larger complex consisting of multiple Rx proteins. **Does this complex contain RanGAP2, a previously identified**

interacting protein of Rx, which is required for extreme resistance to PVX and is also needed for Rx nucleocytoplasmic partitioning (Tameling et al., 2007 Plant Cell; Tameling et al., 2010 Plant Cell)? Unfortunately, this prior work is not mentioned in the current manuscript.

Response to reviewer 2 major comment 3.1:

We thank the reviewer for pointing out the missed citations, we have now included them in our manuscript (Line 219). We would like to point out that the focus of this paper is not to studying Rx-RanGAP2 interactions. Nonetheless, we have attempted to determine whether the Rx complex visualized in our BN-PAGE assays includes RanGAP2 (as described in: Contreras, M. P., & Kamoun, S. (2022). The high molecular weight complex formed by the plant immune receptor Rx does not contain host protein RanGAP2. Zenodo. https://doi.org/10.5281/zenodo.7398800). To this end, we co-expressed Rx and NRC2^{EEE} together with a C-terminally V5-tagged version of RanGAP2 and performed BN-PAGE assays. While RanGAP2 accumulated to high levels, we were unable to detect any obvious signal for RanGAP2 that co-migrates with Rx. It could be that the Rx-RanGAP2 association identified by co-immunoprecipitation by Tameling et al. (2007) exists in very low abundance and is therefore not visualized in our in our assays, or that only a small pool of the total RanGAP interacts with Rx which is masked by the abundance at other MWs. It is also possible that the V5 epitope is hidden when RanGAP2 is in complex with Rx. Nonetheless, studying the dynamics of Rx-RanGAP2 complex formation, is outside of the scope of this paper so we have decided not to include these data in the manuscript.

Reviewer 2 major comment 3.2:

In the companion paper by Hee-Kyung Ahn et al., Fig 1B, D, Rpi-amr1 and Rpi-amr3 when fused with HA migrates at a size ~400 kDa, while Flag-tagged versions of Rpi-amr1 or Rpi-amr3 migrate with a size of ~270 kDa when immunoprecipitated with Flag tag. **Can the authors confirm that the slower migrating band observed for Rx-6xHA is not due to an artifact of the HA tag used in the above tests, but is an inherent property of Rx?**

Response to reviewer 2 major comment 3.2:

Thank you for sharing your concern. In **Appendix Figure S4,** Rx is C-terminally RFP tagged, and we still observe a slower migrating form of around 400 kDa.

Reviewer 2 major comment 4:

4. No evidence is provided that Rx and CP-GFP associate in planta. **BN-PAGE (IB-GFP)/Co-immunoprecipitation data should be provided to test whether CP-GFP co-migrates with Rx-6xHA in vivo.**

Response to reviewer 2 major comment 4:

We thank the reviewer for this suggestion. We do not observe a shift in size for Rx when CP is absent or present (**Figure 1, Figure 2, Figure EV1, Figure EV2, Appendix Figure S4, Contreras & Kamoun, Zenodo**), suggesting that an in vivo complex is not being formed. This is in line with previous literature (Tameling et al. 2007), in which CP was shown not to form a stable complex with Rx, pointing towards an indirect mechanism of recognition. We have added a sentence on this in the manuscript (Lines 222-225).

Furthermore, we have now probed for CP in the same BN-PAGE assays mentioned in response to Reviewer 2 major comment 3.1 (**Contreras & Kamoun, Zenodo**). While we can visualize CP as a slow migrating band of approximately 500 kDa, we cannot see any CP signal at a size matching that of Rx or RanGAP2. This is consistent with literature mentioned above which concludes that CP does not form a stable complex with Rx or RanGAP2 (Tameling et al. 2007). Since the focus of this study is not to determine the mechanism by which Rx perceives CP, we have decided not to include these data in our manuscript.

Reviewer 2 major comment 5:

5. Figure 2B - In the BN-PAGE assays, in the absence of CP, when NRC2EEE is co-expressed with Rx, NRC2EEE (both -mCherry-4xMyc and -4xMyc tagged) migrates as a band of ~200 kDa and ~400 kDa. In the same figure, Rx also migrates as a band of ~400 kDa, coinciding with the slower migrating band of NRC2EEE. A similar migration pattern was observed in Figure S4 when Rx and NRC2EEE was tagged with C-terminal RFP and GFP tags, respectively. **To address if Rx forms a complex with NRC2 before Rx activation, the authors performed the BN-PAGE assay with the "heavy" and "light" versions of the NRC2EEE and Rx and conclude that Rx and NRC2 do not form stable complexes with each other at resting state. Due to inherent limitations of BN-PAGE analysis, it is important to independently validate these results by co-immunoprecipitation assays. In addition, I strongly recommend that the authors independently validate the size of pre-activation Rx and activated NRC2EEE complexes by routine SEC experiments.**

Response to reviewer 2 major comment 5:

We appreciate the reviewer's concern. Regarding the formation of stable sensor-helper complexes at resting state, the working model presented in our study does not rule out protein interactions at resting state. It is true that, while we are unable to visualize a stable pre-activation complex in our BN-PAGE assays, we cannot rule out that a transient or very

low abundance complex exists. Indeed, we postulate that it is possible that the formation of transient sensor-helper complexes could underpin the signaling mechanism between these two components that lead to NRC oligomerization (Lines 457-463).

As for the lack of independent validation, we would like to point out that we consider the membrane fractionation assays and the cell biology experiments provided in **Figures 6 and 8** as orthogonal replication of the Rx/NRC2 working model we propose in **Figure 9**. In addition, in this revised version of the manuscript and as discussed above, we have now included gel filtration analyses to independently validate the Rx-mediated oligomerization of NRC2^{EEE} (Lines 229-244, **Figure EV2**). In these assays we can see a shift in NRC2 elution volume upon CP/Rx activation, indicative of complex formation. The elution volume for this complex suggests that it is between 440 and 669 kDa, which would be consistent with an NRC2 pentamer (550 kDa). We are unable to see a shift in the elution volume for Rx or an increase in Rx signal in the fractions corresponding to activated NRC2^{EEE}, which further supports that Rx is not included in the mature NRC2 complex.

Reviewer 2 major comment 6:

6. Figure 4. The authors nicely show that Bs2 activation with AvrBs2 can trigger NRC2EEE complex formation in a similar manner to Rx, whereas Rpi-blb2 activation with AVRblb2 did not lead to NRC2EEE oligomerization. **To support the claim of the authors that "that sensor-mediated NRC oligomerization is a general principle for NRC dependent sensors" (lines 279 - 280), it is important to show that Rpi-blb2 activation results in NRC4 complex formation (e.g. NRC4EEE oligomer) as Rpi-blb2 is an NRC4-dependent sensor**.

Response to reviewer 2 major comment 6:

We thank the reviewer for this suggestion. As mentioned above (response to major comment 2), we have performed these experiments and shown that, indeed, effector triggered activation of Rpi-blb2 results in NRC4 oligomerization (Lines 316-325, **Figure 5**). We have also made the claim more specific, changing it to "that sensor-mediated NRC2 oligomerization applies to another NRC2 dependent sensor" (Lines 308-309).

Reviewer 2 major comment 7.1:

7. Figure 7. Both Rx and NRC2EEE display cytoplasmic localization in the pre-activated state. Previous studies by Slootweg et al., 2010, Tameling et al., 2010, found that nucleocytoplasmic partitioning of Rx is essential for effective defence signalling. Unfortunately, this prior work is not mentioned in the current manuscript. No nuclear localization of Rx in the activated state was described by the authors. **If NRC2EEE oligomerization depends on transient interaction of activated Rx with NRC2EEE in the cytoplasm, no oligomerization of NRC2EEE is expected when localization of Rx in the nucleus is enforced. It would be very informative if the author could provide such evidence.**

Response to reviewer 2 major comment 7.1:

We appreciate the reviewer's concern. So far, we have ambiguous and quantitative results with nuclear localization signal (NLS) tagging despite our extensive expertise on the topic (Kanneganti et al. Plant Journal 2007; Schornack et al. Plant Journal 2009).

Reviewer 2 major comment 7.2:

In addition, can the authors exclude the possibility that *Agrobacterium*-mediated transient gene OE of the respective gene constructs in *N. benthamiana* leaves confounds meaningful interpretation of the observed subcellular localization of NRC2? **Conclusive evidence could be provided by generating and testing transgenic NRC2EEE-GFP plants driven by native 5' regulatory sequences in the nrc2/3/4 mutant background.**

Response to reviewer 2 major comment 7.2:

We appreciate the reviewer's concern. The *Agrobacterium* assay is routinely used in the study of plant immunity and plant pathogen interactions and yields robust results both in terms of the Hypersensitive Response readout and resistance to the virus. If the proper controls are used and the different readouts (immunity, resistance) and subcellular localizations are correlated then the conclusions should be justified. Note also that the shift in localization we observed for NRC2 is massive as the entire pool of NRC2-GFP is associated with the membrane puncta, which is consistent with the biochemistry. The proposed experiments using transgenic lines with native promoter driven NRC2^{EEE} in *nrc234* background are unrealistic in the context of the time frame of this project but will be considered for follow-up studies.

Reviewer 2 major comment 8:

8. The authors show that the induced NRC2EEE oligomers accumulate in membrane-associated puncta, but **it remains unclear whether the mutant oligomer and/or WT-NRC2 oligomer have ion channel activity**. This could be determined by electrophysiological experiments in oocytes, although this request appears to be beyond the scope of the present study. Nevertheless, **because of the lack of direct evidence for NRC2 ion channel activity and lack of information on the stoichiometry of the NRC2EEE complex in the present work, it is inappropriate to refer to the NRC2EEE oligomer as 'the NRC2 resistosome'. Similarly, Fig. 8 should be removed because, as explained above, the activation-and-release model lacks compelling experimental evidence**.

Response to reviewer 2 major comment 8:

Thank you for these suggestions. As mentioned above (response to comment 5), the SEC assays (**Figure EV2**) included in the revised manuscript suggest that the activated NRC2^{EEE} complex has an elution volume consistent with an NRC2 pentamer (550 kDa), which would be in line with both CC-type NLR resistosomes characterized to date (Wang et al., 2019, Förderer et al., 2022, Zhao et al., 2022). As for the calcium channel activity, we would like to point out, however, that not all resistosomes necessarily exhibit calcium-channel activity (e.g. TIR-NLRs), and as such calcium channel activity (or lack thereof) is not a pre-requisite

for an activated NLR complex to be a resistosome. Furthermore, we consistently refer to the activated NRC2 complex as a putative resistosome or a resistosome-like complex.

As for **Figure 8** (now **Figure 9**, in the revised version of this manuscript), we explicitly mention in the figure legend that this is a working model (essentially a visual abstract) which we find reasonable based on the obtained data. We believe that keeping figure 9 in the manuscript will help readers achieve a better conceptualization of the data shown.

Reviewer 2 major comment 9:

9. Rx-mediated immunity to PVX was originally described as "extreme resistance," i.e., without concomitant host cell death. It was then shown that Rx drives separate virus resistance and cell death responses (Bendahmane et al., 1999). In the present work, the biochemical basis of Rx-mediated immunity to PVX without cell death remains unclear. In Arabidopsis, it is now clear that two NLR signalling nodes act in parallel (EDS1-PAD4-ADR1 and EDS1-SAG101-NRG1), each mediating a potent immune response, but only the former is associated with host cell death. **The use of transient gene OE experiments and their combination with cell death assays in *N. benthamiana* leaves throughout the present study carries the risk of overlooking one/several NRC2/Rx heterocomplex(es) without host cell death activity that are physiologically relevant and different from the NRC2EEE oligomer described.**

Response to major comment 9:

Thank you for this comment. We agree that, indeed, unlike other sensors tested in this and our companion study such as Bs2, Rpi-blb2, Rpi-amr1 and Rpi-amr3, it is possible that Rx is performing additional functions to mediate viral resistance without hypersensitive cell death. Our model doesn't rule this out. This would be an interesting area of research for follow up studies. Our experiments using the well-established and widely used experimental system are appropriately controlled and the data we observe are robust and reproducible.

Reviewer 2 minor points

Reviewer 2 minor point 1:

1. Line 184-186 – To achieve this, we transiently expressed these proteins by agroinfiltration in the absence or presence of PVX CP in leaves of nrc2/3/4 *N.benthamiana* CRISPR lines using agroinfiltration and subjected total protein extracts to BN-PAGE assays – Agroinfiltration occurs twice.

Response to reviewer 2 minor point 1:

Thank you for pointing this out. We have amended this in our revised manuscript.

Reviewer 2 minor point 2:

2. Line 146-148 – How activation of sensor NLRs translates into helper activation, immune

signaling and disease resistance remains a fundamental question in plant immunology. Why this hyperbole? Please replace 'fundamental' by 'important'.

Response to reviewer 2 minor point 2:

We have replaced fundamental for important in our revised manuscript.

Reviewer 2 minor point 3:

3. The introduction is very long for an original research article and should be drastically shortened.

Response to reviewer 2 minor point 3:

Thank you for your suggestion. The introduction falls within journal guidelines for length, and we believe that it covers the required background to facilitate interpretation of the results.

Reviewer 2 minor point 4:

4. I am not convinced by the terminology of sensor-helper NLR "networks" in the present work. From a biochemical point of view, the genetic data only point to different functional redundancies of NRC2/3/4 with the respective sensor NLRs. The term 'networked NLR immune receptors' should be removed.

Response to reviewer 2 minor point 4:

The terminology of NLR networks is used as we have both redundancy (many helpers to one sensor) and convergence (many sensors to one helper). See the linked tweet and the Wu et al. Science 2018 that spells out the concept. Furthermore, this terminology is accepted by the broader community as is the term "networked NLR immune receptors". Please see Wu et al. 2018, Feehan et al. 2020, Wróblewski et al. 2018, Derevnina et al. 2021, Ngou et al. 2021., Zhou et al. 2020, among others.

Referee 3

The work uses BN-PAGE to study the role of Rx and NRC activation by showing resistosome formation in *Nicotiana benthamiana* leaves. Induction of an effector PVX CP (GFP-tagged) induced oligomerisation of NRC2 to a size between 700-900 kDa in presence of sensor RX. Sensor RX and effector form also a complex, independent of NRC2 oligomer.

Reviewer 3 comment 1:

Please indicate in an extra panel (Fig. 1) the composition of each complex and discuss the appearance of the complexes in the gel compared to the mass ladder (eg. Using semilog plots). How many molecules can be included (stoichiometry).

Response to reviewer 3 comment 1:

Thank you for these suggestions. As mentioned in our response to reviewer 2, we have used gel filtration-based size exclusion chromatography (SEC) assays to orthogonally validate our BN-PAGE experiments (Lines 230-244, **Figure EV2**). In these assays, the activated NRC2 complex is present in fractions consistent with a molecular weight between 440 and 669 kDa, which is consistent with an NRC2 pentamer (550 kDa). Such a pentameric complex would be in line with previous literature on activated plant NLR CC-type resistosomes (Wang et al., 2019, Förderer et al., 2022, Zhao et al., 2022). We do not see an equivalent shift for Rx in these assays nor do we see an increase in Rx signal in the fractions corresponding to the activated NRC2 complex.

Reviewer 3 comment 2

Blots from native gels using size shift variants of RX and NRC show that NRC2 resistosome that does not include Rx. In addition, size shifts were used to estimate the composition of oligomers. Please show in additional panel, how did you estimated this and how does this fit to the appearance in the gel.

Response to reviewer 3 comment 2:

Thank you for pointing this out. As mentioned in the response to the comment above, we have included gel filtration assays to orthogonally validate our observations.

Dear Sophien,

We have now received re-review reports from two referees. As you will see, you have addressed their concerns satisfactorily. Before I can finally accept the manuscript though, there are some remaining editorial points which need to be addressed. In this regard would you please:

- Make sure that the final text file (.doc) contains no figures and no track changes
- Add up to five keywords
- Add a data availability statement
- Rename the conflict of interest statement as the "DISCLOSURE AND COMPETING INTERESTS STATEMENT"
- For references with long author lists, limit to 10 authors + et al. in the reference section
- Remove the AC/CrediT section from the text
- Make sure that relevant grant numbers are added to the manuscript, and that these match those entered on our website
- Add a callout to the movie, referred to as Movie EV1
- Add page numbers to the table of contents
- Reorganize Appendix figure Source data file to one file/folder per figure and zip.

The publication of source data, particularly for electrophoretic gels and blots, makes primary data more accessible and transparent to the reader. Please provide me with a PDF file per figure that contains the original, uncropped and unprocessed scans of all or key gels used in the figures. The PDF files should be labeled with the appropriate figure/panel number, and should have molecular weight markers; further annotation could be useful but is not essential. The PDF files will be published online with the article as supplementary "Source Data" files. Source Data can also include Excel tables to accompany your graphs. We anticipate that their inclusion will make your work more discoverable and usable to scientists in the future.

We include a synopsis of the paper (see http://emboj.embopress.org/). Please provide me with a general summary image, statement and 3-5 bullet points that capture the key findings of the paper.

EMBO Press is an editorially independent publishing platform for the development of EMBO scientific publications.

Best wishes,

William

William Teale, PhD
Editor
The EMBO Journal
w.teale@embojournal.org

Referee #1:

The authors addressed all my concerns.

Referee #2:

I would like to congratulate the authors on this revision as they have been very responsive to my comments and questions. Most of my earlier questions and concerns have been addressed and resolved by additional experimentation. For example, the SEC experiment now provides compelling evidence for a size shift of the NRC2EEE complex, but not for Rx.

Nevertheless, I suggest that the authors emphasize in the discussion section that it remains unclear how Rx and Bs2 NLRs transmit effector-dependent signals to the NRC2 helper NLR. Also, to bring the manuscript more in line with previous work on Rx extreme resistance, it would be important to explicitly mention in the discussion that the biochemical basis of Rx-mediated immunity without cell death remains undefined.

Dear William,

We hereby submit the revised version of the manuscript entitled "**Sensor NLR immune proteins activate oligomerization of their NRC helpers "** by Mauricio P. Contreras, Hsuan Pai, Yasin Tumtas, Cian Duggan, Enoch Lok Him Yuen, Angel Vergara Cruces, Jiorgos Kourelis, Hee-Kyung Ahn, Kim-Teng Lee, Chih-Hang Wu, Tolga O. Bozkurt, Lida Derevnina and Sophien Kamoun.

We thank the reviewers for their constructive comments. Based on the suggestions by the referees, we have included additional experiments to our manuscript.

- We have performed BN-PAGE experiments with an NRC2 protein without any mutations in its N-terminus, added in Figure S2.

-We have performed analytical size exclusion chromatography/gel filtration experiments on the activated NRC2 complex in order to orthogonally validate our BN-PAGE experiments. These are added in Figure S3.

-We have included further BN-PAGE experiments with another helper NLR, NRC4. These are added in Figure 5.

- The manuscript has been modified in order to accompany these changes. We also attempted BN-PAGE assays with RanGAP2 as suggested but one of the reviewers, but obtained inconclusive results. These data have been included in the response to reviewers.

We hope these additions address the concerns of the reviewers sufficiently for the manuscript to be acceptable for publication in EMBO journal, and we look forward to hearing from you

Many thanks for your attention and best regards,

Sophien Kamoun

Dear Sophien,

Thank you for making all of the small editorial changes to your manuscript. I am now almost ready to click 'accept', but (and I am sorry to sound so pedantic) there are some small remaining jobs to be done. Would you please:

- add grant numbers BB/V002937/1, BBS/E/J/000PR9795 and BBS/E/J/000PR9796 to the manuscript, and

- zip the movie with its legend and remove the legend from the manuscript file

The synopsis image (which I really like) should be 550 x 300-600 pixels. When I resized your image, there were issues with text resolution. Could you please send a smaller image? If you were to also include a two sentence summary (background then findings) of your work for the synopsis (before the bullet points), this would shorten the time to publication.

Best wishes,

William

William Teale, PhD
Editor
The EMBO Journal
w.teale@embojournal.org

Use the link below to submit your revision:

https://emboj.msubmit.net/cgi-bin/main.plex

All editorial and formatting issues were resolved by the authors.

Dear Sophien,

I am pleased to inform you that your manuscript has been accepted for publication in the EMBO Journal.

Congratulations! I'm really happy EMBO Journal can help to share this fantastic work.

Please note that it is EMBO Journal policy for the transcript of the editorial process (containing referee reports and your response letter) to be published as an online supplement to each paper. If you do NOT want this, you will need to inform the Editorial Office via email immediately. More information is available here: https://www.embopress.org/page/journal/14602075/authorguide#transparentprocess

Your manuscript will be processed for publication in the journal by EMBO Press. Manuscripts in the PDF and electronic editions of The EMBO Journal will be copy edited, and you will be provided with page proofs prior to publication. Please note that supplementary information is not included in the proofs.

You will be contacted by Wiley Author Services to complete licensing and payment information. The required 'Page Charges Authorization Form' is available here: https://www.embopress.org/pb-assets/embo-site/tej_apc.pdf - please download and complete the form and return to embopressproduction@wiley.com

EMBO Press participates in many Publish and Read agreements that allow authors to publish Open Access with reduced/no publication charges. Check your eligibility: https://authorservices.wiley.com/author-resources/Journal-Authors/open-access/affiliation-policies-payments/index.html

Should you be planning a Press Release on your article, please get in contact with embojournal@wiley.com as early as possible, in order to coordinate publication and release dates.

If you have any questions, please do not hesitate to call or email the Editorial Office. Thank you for your contribution to The EMBO Journal.

Yours sincerely,

William

William Teale, PhD
Editor
The EMBO Journal
w.teale@embojournal.org

** Click here to be directed to your login page: https://emboj.msubmit.net

EMBO Press Author Checklist

Corresponding Author Name:	Sophien Kamoun
Journal Submitted to:	The EMBO Journal
Manuscript Number:	EMBOJ-2022-111519R

Sophien Kamoun
The EMBO Journal
EMBOJ-2022-111519R

USEFUL LINKS FOR COMPLETING THIS FORM
The EMBO Journal - Author Guidelines
EMBO Reports - Author Guidelines
Molecular Systems Biology - Author Guidelines
EMBO Molecular Medicine - Author Guidelines

Reporting Checklist for Life Science Articles (updated January
This checklist is adapted from Materials Design Analysis Reporting (MDAR) Checklist for Authors. MDAR establishes a minimum set of requirements in transparent reporting in the life sciences (see Statement of Task: 10.31222/osf.io/9sm4x). Please follow the journal's guidelines in preparing your
Please note that a copy of this checklist will be published alongside your article.

Abridged guidelines for figures
1. Data
The data shown in figures should satisfy the following conditions:
- the data were obtained and processed according to the field's best practice and are presented to reflect the results of the experiments in an accurate and unbiased manner.
- ideally, figure panels should include only measurements that are directly comparable to each other and obtained with the same assay.
- plots include clearly labeled error bars for independent experiments and sample sizes. Unless justified, error bars should not be shown for technical
- if n<5, the individual data points from each experiment should be plotted. Any statistical test employed should be justified.
- Source Data should be included to report the data underlying figures according to the guidelines set out in the authorship guidelines on Data

2. Captions
Each figure caption should contain the following information, for each panel where they are relevant:
- a specification of the experimental system investigated (eg cell line, species name).
- the assay(s) and method(s) used to carry out the reported observations and measurements.
- an explicit mention of the biological and chemical entity(ies) that are being measured.
- an explicit mention of the biological and chemical entity(ies) that are altered/varied/perturbed in a controlled manner.
- the exact sample size (n) for each experimental group/condition, given as a number, not a range;
- a description of the sample collection allowing the reader to understand whether the samples represent technical or biological replicates (including how many animals, litters, cultures, etc.).
- a statement of how many times the experiment shown was independently replicated in the laboratory.
- definitions of statistical methods and measures:
  - common tests, such as t-test (please specify whether paired vs. unpaired), simple χ2 tests, Wilcoxon and Mann-Whitney tests, can be unambiguously identified by name only, but more complex techniques should be described in the methods section;
  - are tests one-sided or two-sided?
  - are there adjustments for multiple comparisons?
  - exact statistical test results, e.g., P values = x but not P values < x;
  - definition of 'center values' as median or average;
  - definition of error bars as s.d. or s.e.m.

Please complete ALL of the questions below.
Select "Not Applicable" only when the requested information is not relevant for your study.

Materials

**Newly Created Materials**	**Information included in the manuscript?**	**In which section is the information available?** (Reagents and Tools Table, Materials and Methods, Figures, Data Availability Section)
New materials and reagents need to be available; do any restrictions apply?	Yes	Materials and Methods

**Antibodies**	**Information included in the manuscript?**	**In which section is the information available?** (Reagents and Tools Table, Materials and Methods, Figures, Data Availability Section)
For **antibodies** provide the following information:<br>- Commercial antibodies: RRID (if possible) or supplier name, catalogue number and or/clone number<br>- Non-commercial: RRID or citation	Yes	Materials and Methods

**DNA and RNA sequences**	**Information included in the manuscript?**	**In which section is the information available?** (Reagents and Tools Table, Materials and Methods, Figures, Data Availability Section)
**Short novel DNA or RNA including primers, probes**: provide the sequences.	Yes	Materials and Methods

**Cell materials**	**Information included in the manuscript?**	**In which section is the information available?** (Reagents and Tools Table, Materials and Methods, Figures, Data Availability Section)
**Cell lines:** Provide species information, strain. Provide accession number in repository **OR** supplier name, catalog number, clone number, and/**OR** RRID.	Not Applicable	
**Primary cultures:** Provide species, strain, sex of origin, genetic modification status.	Not Applicable	
Report if the cell lines were recently **authenticated** (e.g., by STR profiling) and tested for mycoplasma contamination.	Not Applicable	

**Experimental animals**	**Information included in the manuscript?**	**In which section is the information available?** (Reagents and Tools Table, Materials and Methods, Figures, Data Availability Section)
**Laboratory animals or Model organisms:** Provide species, strain, sex, age, genetic modification status. Provide accession number in repository **OR** supplier name, catalog number, clone number, **OR** RRID.	Not Applicable	
**Animal observed in or captured from the field:** Provide species, sex, and age where possible.	Not Applicable	
Please detail **housing and husbandry conditions**.	Not Applicable	

**Plants and microbes**	**Information included in the manuscript?**	**In which section is the information available?** (Reagents and Tools Table, Materials and Methods, Figures, Data Availability Section)
**Plants:** provide species and strain, ecotype and cultivar where relevant, unique accession number if available, and source (including location for collected wild specimens).	Yes	Materials and Methods
**Microbes:** provide species and strain, unique accession number if available, and source.	Yes	Materials and Methods

**Human research participants**	**Information included in the manuscript?**	**In which section is the information available?** (Reagents and Tools Table, Materials and Methods, Figures, Data Availability Section)
If collected and within the bounds of privacy constraints report on age, sex and gender or ethnicity for all study participants.	Not Applicable	

**Core facilities**	**Information included in the manuscript?**	**In which section is the information available?** (Reagents and Tools Table, Materials and Methods, Figures, Data Availability Section)
If your work benefited from core facilities, was their service mentioned in the acknowledgments section?	Yes	Acknowledgements

Design

Study protocol	Information included in the manuscript?	In which section is the information available? (Reagents and Tools Table, Materials and Methods, Figures, Data Availability Section)
If study protocol has been **pre-registered, provide DOI in the manuscript**. For clinical trials, provide the trial registration number **OR** cite DOI.	Not Applicable	
Report the **clinical trial registration number** (at ClinicalTrials.gov or equivalent), where applicable.	Not Applicable	

Laboratory protocol	Information included in the manuscript?	In which section is the information available? (Reagents and Tools Table, Materials and Methods, Figures, Data Availability Section)
Provide DOI OR other citation details if **external detailed step-by-step protocols** are available.	Not Applicable	

Experimental study design and statistics	Information included in the manuscript?	In which section is the information available? (Reagents and Tools Table, Materials and Methods, Figures, Data Availability Section)
Include a statement about **sample size** estimate even if no statistical methods were used.	Yes	Figures and Materials and Methods
Were any steps taken to minimize the effects of subjective bias when allocating animals/samples to treatment (e.g. **randomization procedure**)? If yes, have they been described?	Not Applicable	
Include a statement about **blinding** even if no blinding was done.	Not Applicable	
Describe **inclusion/exclusion criteria** if samples or animals were excluded from the analysis. Were the criteria pre-established?<br><br>If sample or data points were omitted from analysis, report if this was due to attrition or intentional exclusion and provide justification.	Not Applicable	
For every figure, are **statistical tests** justified as appropriate? Do the data meet the assumptions of the tests (e.g., normal distribution)? Describe any methods used to assess it. Is there an estimate of variation within each group of data? Is the variance similar between the groups that are being statistically compared?	Not Applicable	

Sample definition and in-laboratory replication	Information included in the manuscript?	In which section is the information available? (Reagents and Tools Table, Materials and Methods, Figures, Data Availability Section)
In the figure legends: state number of times the experiment was **replicated** in laboratory.	Yes	Figures
In the figure legends: define whether data describe **technical or biological replicates**.	Yes	Figures

Ethics

Ethics	Information included in the manuscript?	In which section is the information available? (Reagents and Tools Table, Materials and Methods, Figures, Data Availability Section)
Studies involving **human participants**: State details of **authority granting ethics approval** (IRB or equivalent committee(s), provide reference number for approval.	Not Applicable	
Studies involving **human participants**: Include a statement confirming that **informed consent** was obtained from all subjects and that the experiments conformed to the principles set out in the WMA Declaration of Helsinki and the Department of Health and Human Services Belmont Report.	Not Applicable	
Studies involving **human participants:** For publication of **patient photos**, include a statement confirming that consent to publish was obtained.	Not Applicable	
Studies involving experimental **animals**: State details of **authority granting ethics approva**l (IRB or equivalent committee(s), provide reference number for approval. Include a statement of compliance with ethical regulations.	Not Applicable	
Studies involving **specimen and field samples:** State if relevant **permits** obtained, provide details of authority approving study; if none were required, explain why.	Not Applicable	

Dual Use Research of Concern (DURC)	Information included in the manuscript?	In which section is the information available? (Reagents and Tools Table, Materials and Methods, Figures, Data Availability Section)
Could your study fall under dual use research restrictions? Please check biosecurity documents and list of **select agents and toxins** (CDC): https://www.selectagents.gov/sat/list.htm	Not Applicable	
If you used a select agent, is the security level of the lab appropriate and reported in the manuscript?	Not Applicable	
If a study is subject to dual use research of concern regulations, is the name of the **authority granting approval and reference number** for the regulatory approval provided in the manuscript?	Not Applicable	

Reporting

The MDAR framework recommends adoption of discipline-specific guidelines, established and endorsed through community initiatives. Journals have their own policy about requiring specific guidelines and recommendations to complement MDAR.

Adherence to community standards	Information included in the manuscript?	In which section is the information available? (Reagents and Tools Table, Materials and Methods, Figures, Data Availability Section)
State if relevant guidelines or checklists (e.g., **ICMJE, MIBBI, ARRIVE, PRISMA**) have been followed or provided.	Not Applicable	
For **tumor marker prognostic studies,** we recommend that you follow the **REMARK** reporting guidelines (see link list at top right). See author guidelines, under 'Reporting Guidelines'. Please confirm you have followed these guidelines.	Not Applicable	
For **phase II and III randomized controlled trials**, please refer to the **CONSORT** flow diagram (see link list at top right) and submit the CONSORT checklist (see link list at top right) with your submission. See author guidelines, under 'Reporting Guidelines'. Please confirm you have submitted this list.	Not Applicable	

Data Availability

Data availability	Information included in the manuscript?	In which section is the information available? (Reagents and Tools Table, Materials and Methods, Figures, Data Availability Section)
Have **primary datasets** been deposited according to the journal's guidelines (see 'Data Deposition' section) and the respective accession numbers provided in the Data Availability Section?	Not Applicable	
Were **human clinical and genomic datasets** deposited in a public access-controlled repository in accordance to ethical obligations to the patients and to the applicable consent agreement?	Not Applicable	
Are **computational models** that are central and integral to a study available without restrictions in a machine-readable form? Were the relevant accession numbers or links provided?	Not Applicable	
If publicly available data were reused, provide the respective **data citations in the reference list**.	Not Applicable	
